# Distinctive DNA sequence features define epigenetic longevity of inflammatory memory

**DOI:** 10.1126/science.adz6830

**Published:** 2026-03-26

**Authors:** Christopher J. Cowley, Sairaj M. Sajjath, Luis F. Soto-Ugaldi, Mara Steiger, Samantha B. Larsen, Thomas Carroll, Douglas Barrows, Alexandra Mattei, Kevin A. U. Gonzales, Wei Wang, Kevin Li, Alexander Meissner, Helene Kretzmer, Dana Pe’er, Elaine Fuchs

**Affiliations:** 1 Robin Chemers Neustein Laboratory of Mammalian Cell Biology and Development, Howard Hughes Medical Institute, The Rockefeller University, New York, NY, USA.; 2 Computational and Systems Biology Program, Howard Hughes Medical Institute, Memorial Sloan Kettering Cancer Center, New York, NY, USA.; 3 Digital Engineering Faculty, Hasso Plattner Institute for Digital Engineering, University of Potsdam, Potsdam, Germany.; 4 Department of Genome Regulation, Max Planck Institute for Molecular Genetics, Berlin, Germany.; 5 Bioinformatics Resource Center, The Rockefeller University, New York, NY, USA.

## Abstract

Tissues harbor memories of inflammation, which heighten sensitivity to diverse future assaults. Whether and how these adaptations are sustained through time and cell division remain poorly understood. We show that in mice, epidermal stem cells store lifelong, functional epigenetic records of psoriasis-like skin flares. Applying deep learning to investigate these chromatin dynamics, we unearth CpG dinucleotide density as a major driver of memory persistence. Although unnecessary for inflammation-induced transcription factors to open and establish memories, CpG-enriched sequences thereafter become essential, reinforcing accessibility across cellular generations by integrating DNA demethylation, methylation-sensitive transcription factors, sequence-intrinsic nucleosome disaffinity, and the nucleosome-destabilizing histone variant H2A.Z. Thus, once activated by inflammation-induced transcription factors, DNA sequences orchestrate persistent poise, imparting long-lasting memory to stress-sensitive genes and profoundly affecting tissue fitness upon recall.

## Abstract (summary)

**INTRODUCTION:**

Organisms adapt and learn from environmental stress. Plants that survive one pathogen broaden their resistance to others. Skin exposed to acute inflammation heals future wounds faster. Although evolutionarily advantageous, these memories can turn maladaptive, elevating chronic disease risks that, for skin, can mean psoriasis or atopic dermatitis; for lung, asthma and chronic respiratory diseases; and for intestine, inflammatory bowel disease. Increasing evidence suggests that inflammatory memories may also prime and perpetuate cancers. As our tissues confront ever-changing environments, ways to minimize maladaptive memories while bolstering beneficial ones become urgent, necessitating an understanding of the molecular mechanisms involved.

Contrasting with adaptive immunity, “trained immunity” is driven solely by epigenetics and extends beyond immune cells to other cell types and tissues. Our current understanding suggests that during inflammation, stimulus-specific transcription factors (TFs) act in concert with the general-stress TF c-FOS/c-JUN [i.e., activator protein 1 (AP1)] to open chromatin around target stress-response genes, establish enhancer-associated histone modifications, and recruit the transcriptional machinery. Cell type–specific TFs take advantage of this accessible state, and as inflammation subsides, they keep memory chromatin open and transcriptionally poised until future stress recalls c-FOS/c-JUN to jump-start the hyperactivation of associated genes.

**RATIONALE:**

The endurance of inflammatory memory lies at the heart of its physiological relevance. In humans, vaccines that provoke trained immunity confer broad pathogen protection for years and even decades. In chronic disease, inflammatory bouts can be resolved for years before they spring back, often with greater severity. Our current understanding of inflammatory memory falls short of explaining this perdurance. Using epidermal stem cells of murine skin, we sought to uncover how functional inflammatory memories last. Epigenetic landscapes are largely reset with cell division, necessitating their reestablishment to be heritably propagated. Is long-lasting memory thus the sole purview of rarely dividing cells and/or ones that exist within a specialized niche? If not, how is epigenetic memory propagated through cellular generations? Do certain memory-associated genes receive priority for longevity, and, if so, what mechanisms endow them with a license for persistence?

**RESULTS:**

Epidermal stem cells exposed to a psoriasis-like skin flare acquire epigenetic memories that subsequently persist after pathology and transcription return to baseline. By investigating their retention over 2 years, we learned that while most memories wane, ~10% persist with long-term functional consequences. By single-cell sequencing, we showed that these adaptations are not limited to rarely dividing cells or to regional locales.

Seeking mechanisms compatible with cell divisions, we applied deep learning to investigate chromatin dynamics from naïve to inflamed to postinflamed states. We unearthed CpG dinucleotide density as a central driver of memory perdurance. CpG-enriched memory domains became enduringly demethylated upon inflammation, binding DNA-methylation–sensitive TFs, displaying sequence-intrinsic nucleosome disaffinity, and readily gaining and sustaining the nucleosome-destabilizing, methylation-antagonizing histone variant H2A.Z. This collective epigenetic signature enabled stable propagation of inflammatory memory through time and cell division. Mining publicly available data, we suggest that this mechanism may entrench long-lasting memory across multiple cell types and inflammatory contexts.

**CONCLUSION:**

Our findings suggest that, once activated by inflammation-induced TFs, DNA sequences orchestrate persistent poise, imparting long-lasting memory to stress-sensitive genes and profoundly affecting tissue fitness.

## Editor’s summary

Epithelial stem cells retain a memory of prior inflammation that augments the response of the skin to subsequent insults. Cowley *et al*. profiled DNA accessibility in epidermal stem cells to understand how a psoriasis-like inflammation in young adult mice was retained in aged animals (see the Perspective by Blot and Sapieha). A small subset of DNA regions that increased in accessibility after inflammation remained open for more than a year and were thus poised to allow access for transcription during this time. These accessible regions were characterized by DNA sequences known as CpG sites, demethylated DNA, and the acquisition of a nucleosome-destabilizing histone variant. These epigenetic modifications to DNA and chromatincan persist as cells divide, allowing the memory of inflammation to be retained by the skin through the lifetime of a mouse —Sarah H. Ross

## Graphical Abstract

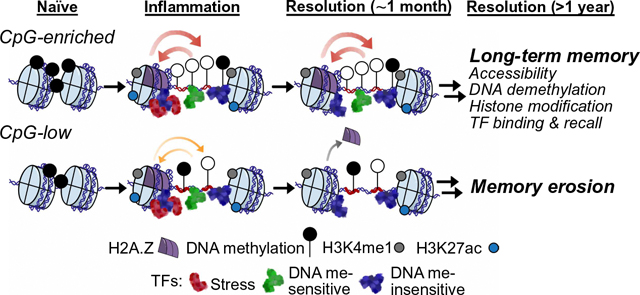

**Model for how longevity of epigenetic memory of stress is determined.** Once memory is established by inflammatory TFs, the specialized sequences of long-term memory domains are exploited by H2A.Z and DNA demethylation. This favors an open-chromatin state and keeps domains accessible for histone modifications and both methylation-sensitive and -insensitive homeostatic TFs.

Organisms have an extraordinary, evolutionarily conserved capacity to adapt to inflammatory stress, which optimizes their reactivity to future encounters—including ones never seen before. Studies on the innate immune system have suggested an epigenetic basis for this “trained immunity,” whereby transient inflammation opens the chromatin of certain stress response genes whose closure is then delayed (1–3). Subsequently, Naik *et al*. (4) found that epithelial stem cells within the innermost (basal) layer of the epidermis can also mount and store epigenetic memories of skin inflammation, which heighten their ability to confront future stresses. This discovery expanded the concept of inflammatory memory beyond the conventional immune system to the tissue itself. Many long-lived cell types are now known to harbor intrinsic epigenetic records of their inflammatory pasts, independent of unresolved stimulation that might elicit persistent changes to transcription (5–13). The consequences to tissue and body fitness can be massive, with both beneficial (e.g., faster wound repair, broader pathogen resistance) (3, 5) and maladaptive outcomes (e.g., chronic inflammation, cancer susceptibility) (11, 14, 15).

Given its epigenetic basis, inflammatory memory was initially speculated to last only on a scale of weeks to months (16), and most mechanistic studies have not looked at timescales relevant to long-term fitness states. In chronic inflammatory disorders such as psoriasis and atopic dermatitis, flares that regress often recur years later (17). Pancreatitis more than doubles the 10-year risk of adenocarcinoma after an episode (18), and the Bacille Calmette-Guérin vaccine can provide years-long protection against antigenically unrelated *Mycobacterium tuberculosis* (19, 20). Similarly, natural killer cells from individuals with latent human cytomegalovirus can sustain epigenetic inflammatory records for more than a year (21), and dermal fibroblasts can archive acute irradiation for more than a decade (11).

How stressful experiences can be epigenetically engrained with such stability remains unanswered yet stands at the crux of tissue and organismal health. Previous studies have shown that during an acute inflammatory experience, stimulus-specific transcription factors (TFs) such as STATs (signal transducers and activators of transcription) and C/EBP (CCAAT/enhancer binding protein) can bind cognate sites in closed chromatin (4, 13). If these sites are near an AP1 (activator protein 1) TF motif, then c-FOS/c-JUN–family heterodimers, induced by general stress, cooperate with the stimulus-specific TFs to remodel this chromatin and establish an inflammation-dependent active state for target genes (22, 23). This state is typified by the enhancer-associated histone modifications H3K4me1 and H3K27ac as well as homeostatic TFs that exploit newfound accessibility and allow “memory domains” to remain open even after stress-induced TFs wane, transcription resolves, and homeostasis restores.

Upon secondary challenge, the chromatin is already poised, and hence stimulus-specific TFs are dispensable for recalling memory (22). By contrast, c-FOS is required. Once activated by any of a diverse array of stresses, c-FOS is rapidly rerecruited to memory domains to accelerate transcription of associated genes. Indeed, AP1 (c-FOS/c-JUN) appears to be generalizable in unifying memory establishment and rapid recall across a near-universal range of physiological contexts (5).

These prior studies have provided valuable insights into which gene cohorts become targeted for intrinsic epigenetic memories of a particular inflammatory experience and how these memories are established and recalled. However, if, as it seems, epigenetic memory is at the root of recurrent inflammatory events spanning years and even decades, then cell type –specific TFs and enhancer-associated histone modifications may be inadequate to explain the longevity of such phenomena. During mitosis, for instance, such marks are typically erased, necessitating their initial establishing factors to be restored in the newly divided cell (24). It has thus been postulated that epigenetic memories might persist only in particular niches and/or infrequently dividing cells. Alternatively, if cells can retain memory through multiple divisions and irrespective of a specialized niche, a compatible mechanism must account for this enduring, inheritable maintenance.

In this study, we tackled these fundamental issues by investigating what happens over a mouse’s lifespan to epigenetic memories mounted during an acute inflammation in young adulthood. We use epidermal stem cells (EpdSCs) of murine skin as our paradigm because of their abundance, long-lived tissue residence, and spatial stability as well as the well-defined TFs and histone modifications that underlie the establishment, early maintenance, and recall of their memories after an acute inflammatory experience (22). We also exploit the fact that EpdSCs exhibit heterogeneity in their cell cycle behaviors and regional locales and niches (25–27), enabling direct assessment of the contributions of each toward the longevity of epigenetic memory.

## EpdSCs epigenetically remember a psoriatic skin flare for life

Six-day topical application of imiquimod (IMQ) onto murine back skin triggers a robust psoriasis-like type 17 inflammation that self-resolves within 30 days. This acute inflammation causes ~40,000 EpdSC chromatin domains to gain accessibility, ~1000 of which remain open after inflammatory pathology and transcription resolve (4, 22, 28). These IMQ-endowed memory domains are cooperatively opened by pSTAT3 (phosphorylated signal transducer and activator of transcription 3) and the c-FOS/c-JUN AP1 complex (22). Although neither pSTAT3 nor c-FOS persist after inflammation, their activity facilitates sustained binding of endogenous EpdSC TFs and enhancer-associated histone modifications. Thus, as pathology resolves, inflammatory memory becomes recognizable primarily at the chromatin level, which remains transcriptionally poised until a subsequent encounter with stress.

**Fig. 1.**
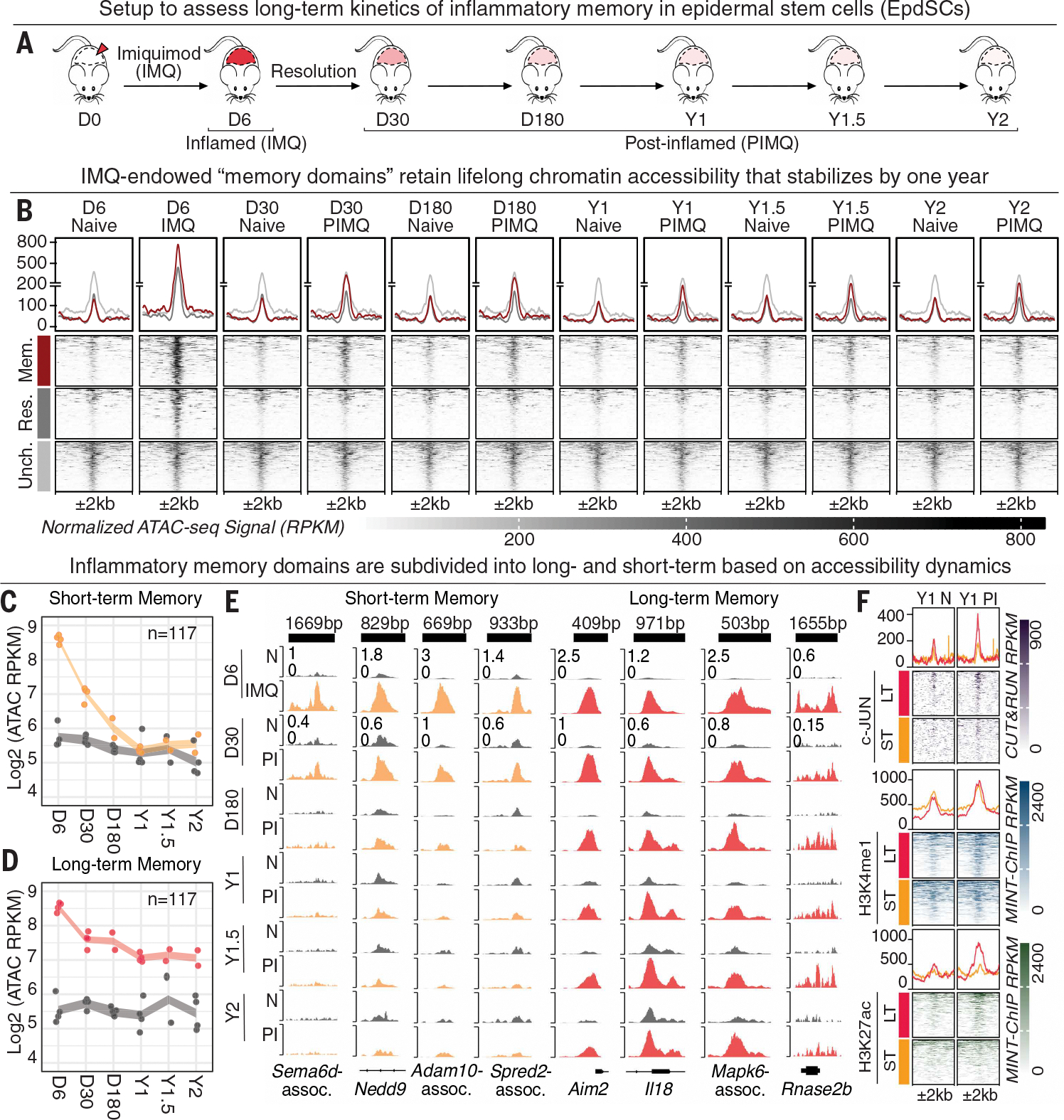
Transient skin inflammation imparts years-long epigenetic memories to EpdSCs. (**A**) Strategy to induce psoriasis-like inflammation with IMQ then track resolution through the murine lifespan. (**B**) Heatmaps of ATAC-seq signal in chromatin of EpdSCs isolated at the indicated times. Each row represents one ATAC peak categorized according to “Memory” domains (Mem.) opened upon IMQ and remaining open through ≥D30 after inflammation (PIMQ), “Resolved” domains (Res.) opened upon IMQ but closed PIMQ, and “Unchanged” domains (Unch.) refractory to inflammation or aging. Signals were normalized as RPKM and merged across biological replicates. *n* ≥ 2 biological replicates per condition, with two or three mice pooled per biological replicate. Data represent one experiment per time point, with the exceptions of D6, D30, and Y1 (two independent experiments each). kb, kilobase pairs; D, day; Y, year. See methods for full replicate list and category definitions. (**C** to **D**) Average ATAC-seq signal across (C) short-term memory domains retained through D30 then resolved by Y1 and (D) long-term memory domains retained through at least Y1. Signals represented by log_2_(RPKM+1) transformation. Each dot represents one biological replicate. Colored points indicate signal in IMQ-exposed EpdSCs, and black points indicate signal in corresponding naïve conditions. (**E**) ATAC-seq tracks of representative ST and LT memory domains, normalized as counts per million (CPM). (**F**) RPKM-normalized signals of Y1 PIMQ (P) and naïve (N) EpdSCs within ST and LT memory domains for indicated transcription factor and histone modifications. Signals averaged acrossa biological replicates, with *n* = 2 biological replicates per condition and two or three mice pooled per biological replicate. Data represent one experiment per mark. See also figs. S1 and S2 and methods.

To address how memories are propagated over time, we performed ATAC-seq (assay for transposase-accessible chromatin with high-throughput sequencing) of purified EpdSCs over the entire mouse lifespan (Fig. 1A; fig. S1, A and B; and data S1). We identified 934 initial memory domains, which opened during inflammation [day 6 (D6) IMQ] then retained heightened accessibility through at least D30 [DESeq2 adjusted *P* value (*P-adj*) < 0.05, log_2_ fold change > 0; methods] (fig. S1C). For comparison, we selected equivalently numbered subsets of (i) “resolved domains” that gained accessibility during inflammation and then returned to naïve levels by D30 and (ii) “unchanged domains” that showed no change with either inflammation or age over the 2-year timecourse (Fig. 1B, fig. S1C, and data S2).

Aggregate memory domain accessibility steadily declined over 1 year, reflecting an erosion of epigenetic adaptations. However, and not apparent on the shorter timescales previously examined (4, 22), accessibility leveled thereafter, with the remaining degree of openness persisting through life (Fig. 1B).

## The existence of long- and short-term inflammatory memories uncoupled from age-related changes to the genome

The marked persistence of memory accessibility intimated the existence of a subset of highly resilient domains, which we hypothesized might sustain lifelong adaptation. Consistently, IMQ-induced memory domains spanned a spectrum of endurance, in which 117 “long-term” memory domains persisted robustly through at least year 1 (Y1), while their more erosive counterparts closed to varying degrees (DESeq2 *P-adj* < 0.05; methods and data S3 to S5). In line with a role in stabilizing aggregate memory from Y1 onward, long-term memory domains remained largely unperturbed even through Y2 (Fig. 1, C to E).

For comparative purposes, we selected an equivalently sized set of 117 “short-term” memory domains from the 934 (Fig. 1, C to E, and data S6), chosen as the loci with the closest accessibilities to naïve baseline by Y1 [DESeq2 Wald statistic values closest to 0 between Y1 post-IMQ (PIMQ) versus Y1 naïve; methods]. Although these domains were comparably open to their long-term counterparts until D30 PIMQ, thereafter, their accessibility began to wane. Adding to these distinctions were longevity differences in previously described features of epigenetic memory, namely c-JUN (endogenously expressed in EpdSCs), H3K4me1, and H3K27ac (Fig. 1F). Although these modifications marked the entire cohort of memory domains at D30 PIMQ (22), only long-term memory domains retained these marks at Y1, as judged by Cut-and-Run (CNR) TF mapping (29) and multiplexed T7-indexed chromatin immunoprecipitation (MINT-ChIP) of histone modifications (30).

Both erosion and retention of epigenetic memory were largely independent of aging itself. Even for the few (4.2%) memory domains that opened with age, this age-dependent opening was modest compared with the robust accessibility induced and sustained after acute inflammation (DESeq2 *P-adj* < 0.05) (fig. S2, A and B, and data S7). Likewise, inflammatory experience in youth did not lead to marked deviations in the natural course of aging. Tissue pathology, epidermal thickness, and EpdSC proliferation were similar between age-matched naïve and postinflamed skins at Y1, with no signs of active inflammation (fig. S2, C to E). Consistently, although our data recapitulated known age-related rewiring in skin immune composition (31–33), these changes were not altered by inflammatory experience (fig. S2, F to G). Lastly, EpdSCs themselves underwent similar epigenetic aging regardless of inflammatory experience, as by Y1, only a few chromatin changes outside of long-term memory domains distinguished the PIMQ state (fig. S2H). Thus, memory persistence reflected bona fide epigenetic adaptation to long-past acute inflammatory stress.

### Long-lasting epigenetic memories functionally endow lifelong hypersensitivity to stress

**Fig. 2.**
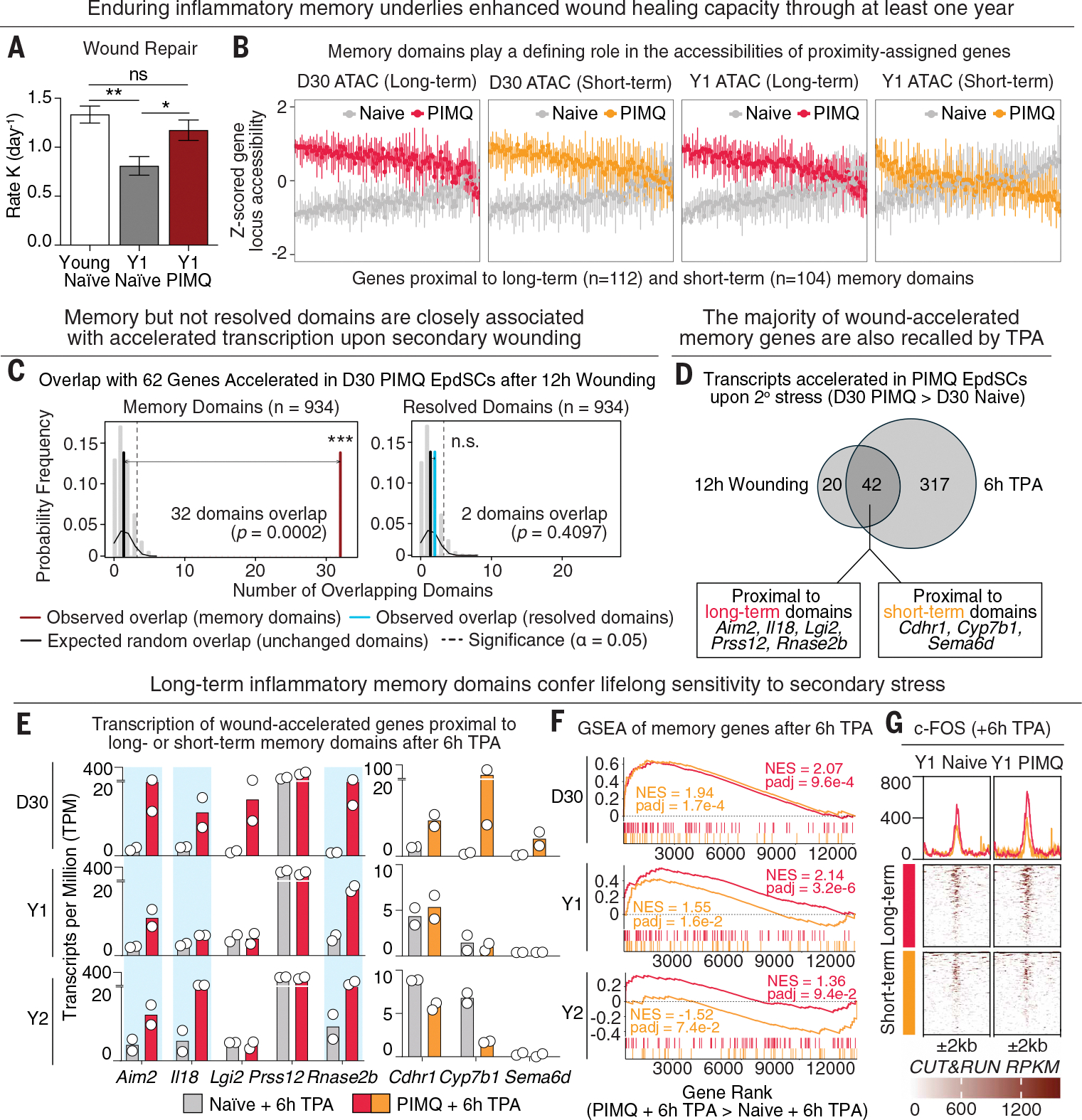
A subset of highly resilient chromatin domains sustains lifelong adaptation to inflammatory stress. (**A**) Rate (κ) of wound closure in young naïve (D6 or D30) and Y1 naïve and PIMQ skins. *n* ≥ 6 mice per condition, with ≥2 wounds per mouse performed as technical replicates. **P = 0.0016; *P = 0.030; ns, not significant (two-tailed unpaired Student’s *t* tests). Data represent two independent experiments. (**B**) Accessibilities of genes associated with ST or LT memory domains, *z*-scored across all biological replicates at the indicated time point. Genes are ordered along the *x* axis by the difference in average locus accessibility between PIMQ and naïve samples (see methods for gene score calculation). (**C**) Permutation test for overlap between genes accelerated 12 hours after wounding in PIMQ EpdSCs (4) and genes associated with either all memory domains (left) or all resolved domains (right). (**D**) Intersection between genes rapidly induced 12 hours after wounding (4) and 6 hours after TPA administration in D30 PIMQ over naïve EpdSCs (22). Representative memory-associated transcripts accelerated upon both secondary stresses are annotated in boxes. (**E**) EpdSC gene expression, normalized as transcripts per million (TPM), after 6 hour TPA exposure to skins of D30, Y1, and Y2 mice. Each dot represents one biological replicate, and each bar represents the average expression across biological replicates. Light-blue boxes highlight examples of memory genes with lifetime hyperresponsiveness after acute inflammation. *n* = 2 biological replicates per condition, with two or three mice pooled (D30, Y1) or one mouse (Y2) per biological replicate. Data represent one experiment per time point. (**F**) GSEA of short-term (orange) or long-term memory-associated genes (red) in RNA-seq data of D30, Y1, and Y2 PIMQ versus age-matched naïve EpdSCs treated with TPA for 6 hours. Note the selective advantage of LT memory genes for rapid responsiveness at Y1 and Y2. NES, normalized enrichment score. (**G**) RPKM-normalized signals of c-FOS binding upon 6 hour TPA treatment at Y1. Note that relative to naïve controls, enhanced c-FOS recruitment is apparent only in LT memory domains. Signals averaged across biological replicates, with *n* = 2 biological replicates per condition and two or three mice pooled per biological replicate. Data represent one experiment. See also fig S3.

Inflammatory memories in EpdSCs heighten the skin’s ability to heal wounds at D30 PIMQ (4). We wondered whether the memories still lingering at Y1 were sufficient to confer enhanced reparative function to aging skin, known to heal slowly in mice as well as humans (31, 34). Accordingly, although aged naïve mice exhibited a ~40% reduction in overall rate of wound-induced reepithelialization compared with their young-adult counterparts, acute inflammation experienced early in adulthood restored aging skin to its youthful wound-regenerative capacity (Fig. 2A).

We posited that the roots of accelerated wound repair should reside within genes poised by EpdSC memory domains for rapid response upon injury. Consistently, at D30 after inflammation, nearly all EpdSC gene loci in proximity to or containing a memory domain were elevated in overall accessibility, whereas by Y1, only long-term memory domain–associated genes sustained this advantage (Fig. 2B). These gene-level accessibilities were closely linked to accelerated transcription upon secondary wounding. At D30, 62 transcripts were more robustly elevated within 12 hours of wounding in postinflamed EpdSCs (DESeq2 log_2_ fold change > 1, *P-adj* < 0.05), among which more than one-third were linked to inflammatory memory domains (Fig. 2B and data S8). This association was much higher than random chance (i.e., mapping of random unchanged domains to wound-accelerated genes), in marked contrast to that of resolved domains that were sensitive to IMQ but closed rapidly thereafter (Fig. 2C and fig. S3A).

Among the 62 transcripts displaying heightened wound sensitivity was the inflammasome effector *Aim2*, which is also associated with a long-term memory domain (Fig. 1E). Upon wounding of postinflamed skin, AIM2 activation in EpdSCs enhances healing through caspase-mediated activation of interleukin-1β (IL-1β) (4), also a central effector of inflammatory memory in innate immune cells (35). Other stress-responsive transcripts included long-term memory–associated genes encoding the inflammatory cytokine IL-18 and antimicrobial and antiviral ribonuclease and chemoattractant RNase2b as well as short-term memory genes *Cdhr1*, encoding a mediator of cell-cell integrity (36), *Cyp7b1*, which activates cellular proliferation during inflammation (37), and *Sema6d*, which regulates cell migration in development and stress (38) (data S6 and S8).

Although the frailty of aged mice precluded extensive wound studies, the similar memory profiles between Y1 and Y2 EpdSC chromatin were suggestive that memory of acute inflammation not only persists throughout the mouse’s lifetime but is also sufficient to impart EpdSCs with lifelong consequences for tissue fitness against stress. To test this hypothesis, we shifted to the protein kinase C activator TPA (12-*O*-tetradecanoylphorbol-13-acetate) to molecularly evaluate secondary stress responses (data S9 to S11). After 6 hours of TPA treatment at D30 PIMQ, nearly 70% of the genes with enhanced responsiveness 12 hours after wounding also displayed hypersensitivity to this stimulus (Fig. 2D).

Similar to wounding, the heightened stress sensitivity of short-term memory genes to TPA waned by Y1, but long-term memory–associated genes, including *Aim2*, *Il18*, and *Rnase2b*, continued to display heightened sensitivity even 2 years after acute inflammation (Fig. 2E). Moreover, gene set enrichment analyses (GSEA) suggested that although memory-associated genes were enriched comparably among up-regulated transcripts in postinflamed EpdSCs upon TPA at D30, only long-term memory–associated transcripts robustly retained this enrichment when exposed to TPA at Y1 or Y2 (Fig. 2F).

Consistent with a recent study correlating the rapidity of a gene’s induction with the proximity of its cis-regulatory elements to the transcription start site (39), *Aim2* and other long-term memory domains with rapid (6 hour) responsiveness to secondary stress (TPA) tended to reside nearer to gene promoters (Fig. 2E and data S2 and S8). We then used secondary stress–induced c-FOS recruitment as a proxy to measure broader memory recall dynamics (22). After TPA exposure at D30 postinflammation, EpdSCs displayed elevated c-FOS across memory domains (fig. S3B). By Y1, however, only long-term memory domains retained this heightened occupancy and did so across nearly the entire cohort (Fig. 2G). These findings implied that despite an apparent importance of promoter proximity toward the rapidity of memory recall, the enduring accessibility of long-term memory domains permitted its core mechanisms to remain operational across the genome, even years after acute stimulation of EpdSCs.

### Long-lasting inflammatory memories are ubiquitously harbored across the basal epidermis

During skin homeostasis, although most EpdSCs proliferate every ~7 days (25), a small subset of highly quiescent EpdSCs has been described (40). In addition, the EpdSCs of the interfollicular epidermis (25) and the orifice of the upper hair follicle (26, 27) reside in distinct niches that differ in their interactions with the external environment and the immune system. We thus postulated that memory longevity might be the purview of specialized EpdSCs, privileged either by quiescence or their immediate niche.

**Fig. 3.**
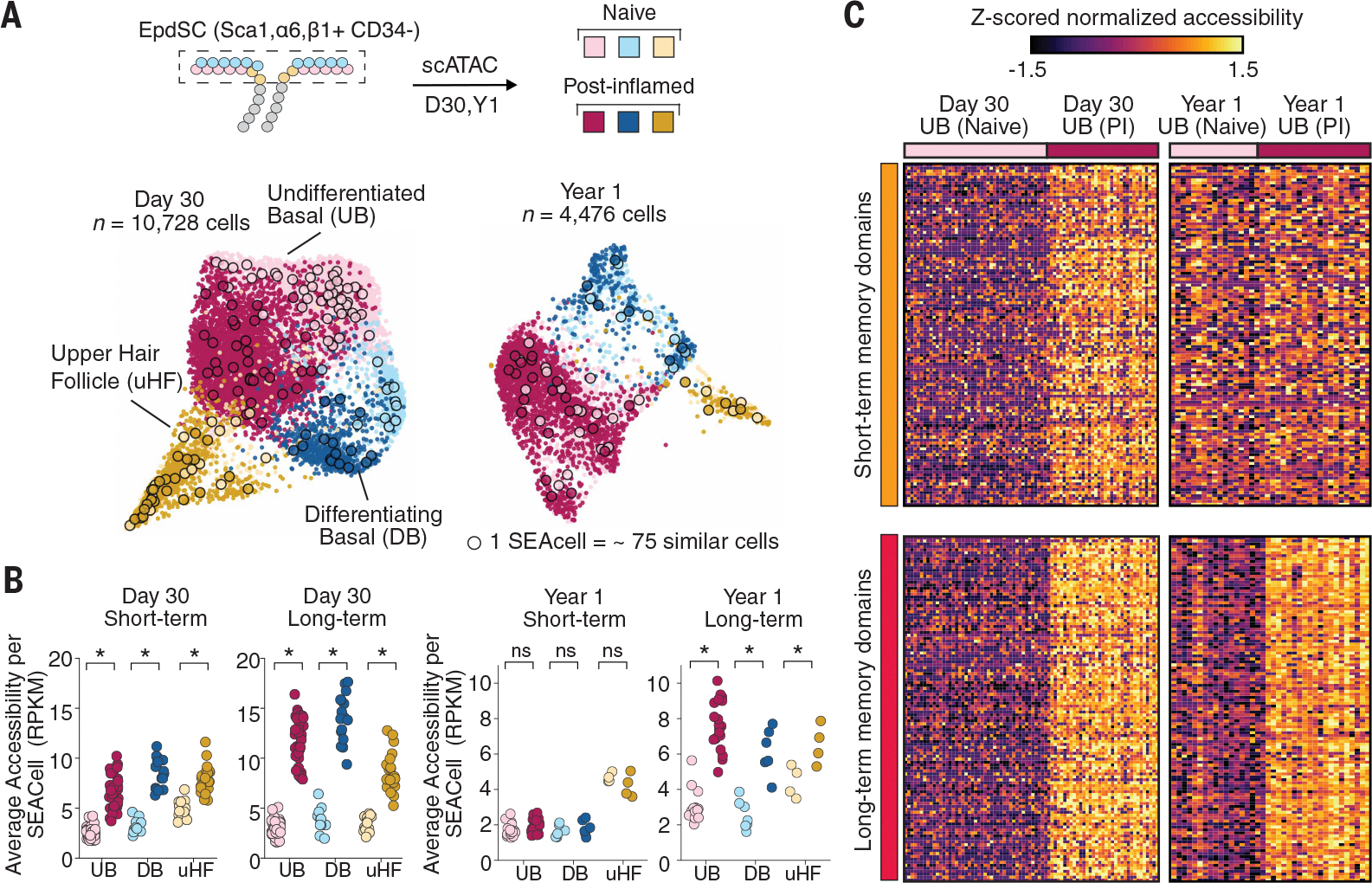
Long-term chromatin accessibility of memory domains is a feature of most if not all inflammation-experienced basal epidermal progenitors. (**A**) (Top) EpdSC purification schematic for scATAC-seq. (Bottom) Force directed layout embedding of indicated cell types based on scATAC-seq profiles at D30 (left) or Y1 (right). Each small point represents one cell, and large points represent mean positions of metacells (“SEACells”) grouped to improve signal-to-noise ratio. *n* = 4 mice pooled per each naïve or PIMQ condition. Data represent one experiment. (**B**) Average RPKM-normalized accessibilities across ST or LT memory domains within each SEACell in the indicated populations and time points. Each dot represents one SEACell and is colored as in (A). *P < 0.001; ns, not significant (two-tailed unpaired Wilcoxon test). (**C**) Heatmap showing relative (*z*-scored) intensity of RPKM-normalized scATAC-seq signals per SEACell within ST or LT memory domains at D30 or Y1, with each column representing one SEACell in the undifferentiated basal (UB) (interfollicular EpdSCs) compartment and each row representing one ATAC-seq peak. *Z*-scores per domain were independently calculated for each time point. See also figs. S4 and S5.

To address these possibilities, we performed single-cell ATAC and RNA sequencing (scATAC-seq and scRNA-seq, respectively) of basal epidermal keratinocytes at D30 and Y1. scATAC (Fig. 3A) and scRNA profiles (fig. S4A) both revealed three major populations: undifferentiated basal (UB) EpdSCs, representing bona fide stem cells of the interfollicular epidermis; their immediate differentiating basal (DB) progeny, which transiently exist within the basal layer before moving upward (41); and EpdSCs at the orifice of the upper hair follicle (uHF) (Fig. 3A and fig. S4A).

Temporal scRNA-seq revealed that inflammation-induced transcription largely returned to baseline across basal populations from D30 onward (fig. S4B and data S12 to S15). There were a few exceptions, most notably *Aim2* within UB EpdSCs. Overall, however, the wide-spread transcriptional return to normalcy throughout individual stem cell pools argued against the notion that persistent, inflammation-induced alterations in signaling from the niche microenvironment contributed to these persistent epigenetic adaptations.

To assess whether the capacity to retain epigenetic inflammatory memory was restricted to a subset of basal epidermal cells, we turned to scATAC-seq. Given the low signal-to-noise ratio inherent to this assay, we used the SEACells algorithm (42) to define “metacells” (hereafter, “SEACells”), each comprising ~75 highly similar single cells allowing for deepened ATAC signal while largely preserving the granularity of single-cell profiling. We additionally focused analyses on accessible chromatin domains that had been determined by bulk ATAC-seq.

Force-directed layout (FDL) embedding of scATAC-seq profiles cleanly demarcated naïve from PIMQ cells within UB and DB compartments at D30 (Fig. 3A). Although no such clear difference was observed in the Y1 FDL embedding, analysis of variably accessible motifs through the ChromVAR algorithm (43) indicated robust chromatin opening over AP1 sites across PIMQ but not naïve SEACells (fig. S5A). These data were in agreement with the sustained accessibility and c-JUN binding that we had seen in long-term memory domains.

The endurance and erosion of nearly all bulk-defined memory domains were conserved across nearly all SEACells in both the short (D30) and long (Y1) terms (Fig. 3, B and C; fig. S5B; and data S16 and S17). The modest structure introduced by hierarchical clustering (fig. S5C) closely tracked with sequencing depth across SEACells, consistent with known depth-driven effects in sparse scATAC-seq datasets rather than underlying biological heterogeneity (44). Similarly conserved dynamics were observed over resolved and unchanged regions (fig. S5D). Thus, long-lasting inflammatory memory was neither confined to a subpopulation of quiescent EpdSCs nor differentially compartmentalized within distinct niche microenvironments. Rather, transient acute inflammation appeared to train nearly the entire EpdSC population, with enduring cell-intrinsic consequences that extended over multiple divisions in both early (D30, ~3.5 divisions per EpdSC) and late (Y1, ~50 divisions) stages of resolution.

### Known epigenetic regulators of memory establishment do not determine longevity

**Fig. 4.**
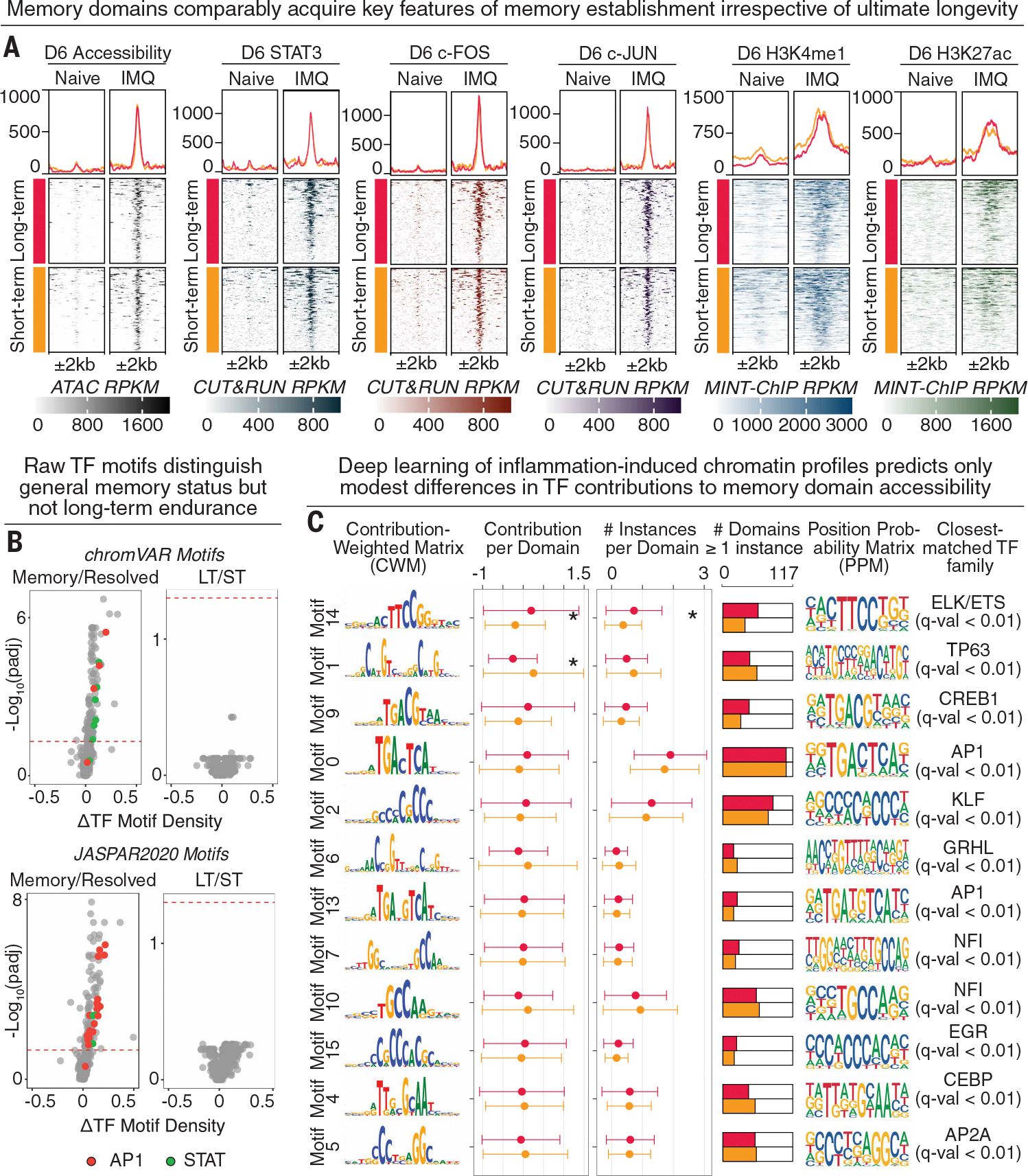
Regulators of memory establishment are uncoupled from the determinants of ultimate longevity. (**A**) Chromatin accessibility, TF binding, and histone modifications over LT and ST memory domains during IMQ inflammation (D6) (22). For all panels, signal was RPKM-normalized and merged across biological replicates. (**B**) Motif enrichment analysis comparing the indicated domains using chromVARmotifs (top) and JASPAR2020 (bottom) databases. Each dot represents one TF motif, with differences in motif densities across conditions on the *x* axis and statistical significance (two-tailed unpaired Wilcoxon test, Benjamini-Hochberg adjusted *P* value) on the *y* axis. Note that these standard motif-scanning methods distinguish memory from resolved domains but fail to discriminate between LT and ST memory. Dashed red line: *P-adj* = 0.05 cutoff. (**C**) De novo motif discovery from D6 IMQ ChromBPNet model. (Left) Contribution weight matrices derived from genome-wide motif instances for each de novo motif, determined by DeepLIFT and TF-MoDISco (hit correlation ≥ 0.75, hit coefficient > 2). The height of each nucleotide represents its relative contribution to accessibility during IMQ inflammation. (Middle) *Z*-score–normalized contribution scores and number of motif instances averaged across all instances per LT (red) or ST (orange) memory domain. *P-adj < 0.05 (two-tailed unpaired Student’s *t* test). (Right) Number of LT or ST domains with ≥1 motif instance, position probability matrices derived from motif instances across all LT and ST memory domains, and closest TF family matches identified using TOMTOM against the JASPAR database. See also fig. S6.

Having ruled out quiescence or a specialized niche as requirements to sustain epigenetic memory, we tested whether long-term memory loci might be more robustly opened and/or remodeled during primary inflammation (D6 IMQ) and thus cross some threshold for subsequent endurance. However, during the height of inflammation, both long- and short-term memory domains acquired comparable chromatin accessibilities and similar inductions of their associated transcripts (Fig. 4A and fig. S6A). During this time, both sets also displayed comparably robust STAT3 and c-FOS/c-JUN (AP1) binding and similar occupancies by H3K4me1- and H3K27ac-containing histones (Fig. 4A). Additionally, conditional *Stat3* genetic knockout or AP1 inhibition equivalently reduced inflammation-induced domain accessibilities (fig. S6B). Thus, although STAT3, AP1, H3K4me1, and H3K27ac demarcated memory domains as a whole during acute inflammation, their binding intensities and functional activities did not account for ultimate longevity.

Positing that additional regulators might delineate establishment of long-term versus short-term memory, we next scanned domain sequences for canonical TF motifs. However, no motifs across two curated databases clearly differentiated the two cohorts (Fig. 4B and data S18 and S19) (45). To probe more deeply, we turned to ChromBPNet (46), a recently developed deep-learning framework that learns DNA sequence patterns predictive of chromatin accessibility. Unlike database-driven motif searches, ChromBPNet models the base-resolution relationship between raw DNA sequence and experimentally measured accessibility, deconvolving assay biases and identifying which short DNA patterns most strongly predict the observed accessibility landscape. By analyzing predicted contributions of these sequences to accessibilities of our memory domains at the height of inflammation (D6), we could then identify whether specific, functionally predictive DNA sequence patterns distinguish long-term from short-term memory establishment beyond what standard motif scans reveal.

We trained ChromBPNet on our inflammation-induced (D6 IMQ) ATAC data and found that the learned model accurately predicted observed accessibility from DNA sequence alone (Pearson correlation, *r* = 0.65) (fig. S5C, methods, and data S20). Attesting to the ability of our model to identify biologically meaningful sequence motifs within memory domains, ChromBPNet successfully recovered experimentally validated high-contribution instances of de novo motifs for AP1 (fig. S6D).

Our model independently ranked an AP1 motif as the dominant contributing sequence pattern across nearly all memory domains, reflecting its status as a conserved memory driver (Fig. 4C). However, the predicted contribution of this motif to accessibility was similar between long- and short-term memory cohorts. ChromBPNet also predicted contribution of several other sequences to inflammation-induced memory establishment (data S21). These sequences resembled known motifs of abundant epidermal homeostatic TFs, including KLF (Kruppel-like factor) and NFI (nuclear factor I) family members, but most, like AP1, did not distinguish memory domains in either abundance or predicted contribution to accessibility (Fig. 4C). The only motifs that did so were for TP63 (tumor protein p63; modestly enriched in the short term) and the ELK/ETS family motif (modestly favoring the long term). However, for both motifs, fewer than half of memory domains contained a site predicted to contribute to their establishment.

### Deep learning of temporal chromatin dynamics predicts that inflammatory memory longevity is intrinsic to domain sequence

**Fig. 5.**
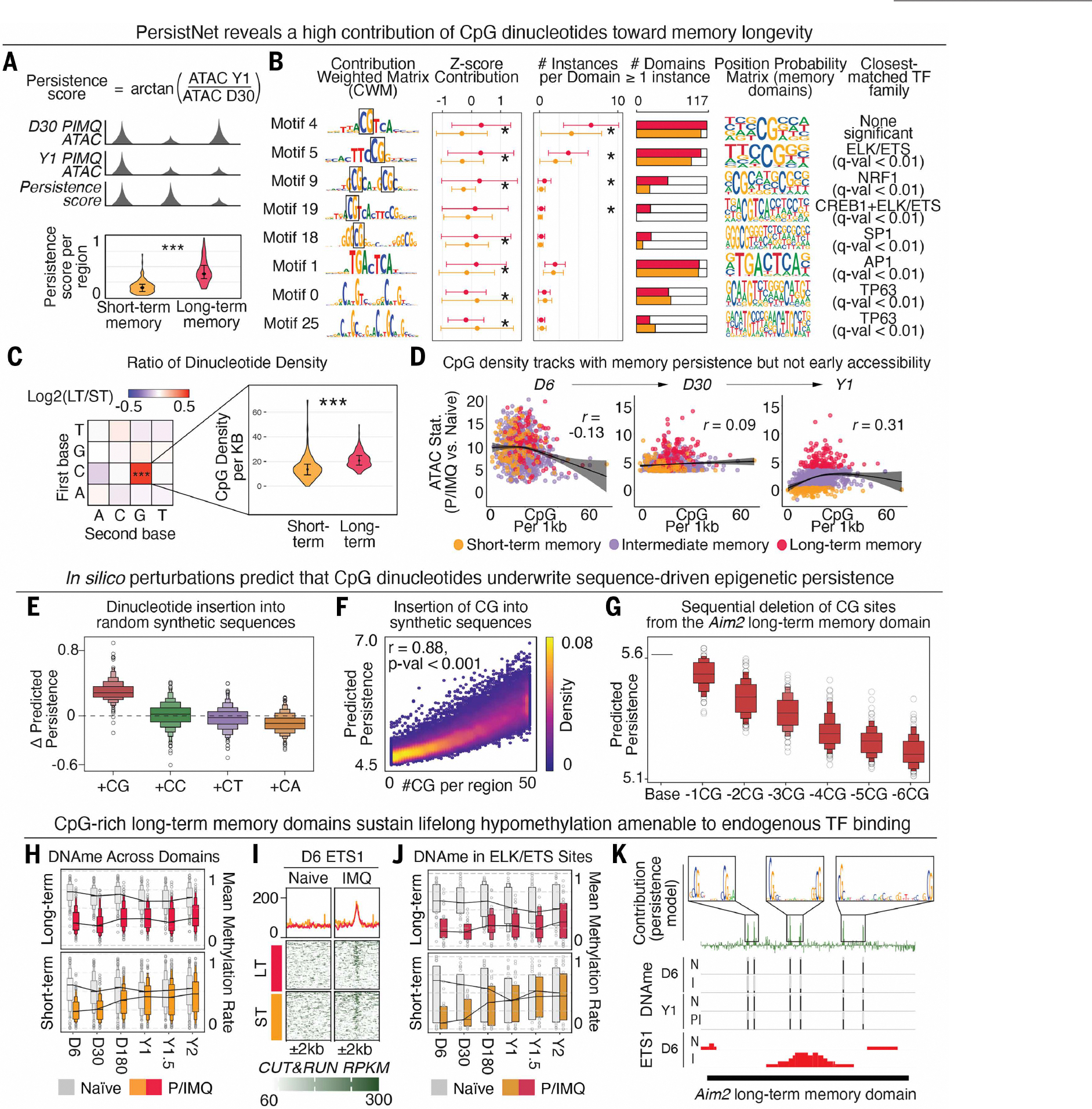
CpG-enriched DNA sequences determine the longevity of inflammatory memory domains. (**A**) (Top) Formula and representative schematic of per-base “persistence scores” for PersistNet model. (Bottom) Violin plots of actual persistence scores comparing accessibility persistence from D30 to Y1 PIMQ in ST and LT memory domains. ***P < 0.001 (two-tailed unpaired Wilcoxon test). (**B**) De novo motif discovery from PersistNet model trained on persistence scores as in (A). (Left) Contribution-weighted matrices derived from genome-wide motif instances of each de novo motif (hit correlation ≥ 0.75, hit coefficient > 2). The height of each nucleotide represents its relative contribution to accessibility persistence. (Middle) *Z*-score–normalized contribution scores and average number of motif instances per LT (red) or ST (orange) domain. *P-adj < 0.05 (two-tailed unpaired Student’s *t* test). (Right) Number of LT or ST domains with ≥1 motif instance, position probability matrices derived from motif instances across all LT and ST memory domains, and closest TF family matches identified using TOMTOM against the JASPAR database. (**C**) (Left) Log_2_ ratios of densities per kilobase of all possible dinucleotide combinations within LT over ST memory domains. (Right) CpG density per kilobase across memory domains. ***P < 0.001; ns, not significant (two-tailed unpaired Wilcoxon test). (**D**) Scatterplots relating CpG density of ST, LT, and intermediate memory domains to their differential chromatin accessibility (DESeq2 ATAC-seq Wald statistic) in P/IMQ over naïve EpdSCs through life. Each dot represents a memory domain. Black lines show smoothed signals across plotted domains using a generalized additive model, with gray ribbons indicating standard error. *r* values indicate Spearman’s rank correlation coefficients. Note robust association of CpG with memory persistence (Y1) but not inflammation-induced accessibility (D6) or early retention (D30). (**E**) Predicted persistence scores of 500 synthetic DNA sequences before and after inserting different dinucleotide sequences in silico. (**F**) Scatterplot showing that steadily increasing the number of CGs within random DNA sequences predicts a continual increase in persistence score. Each point represents one in silico DNA sequence. *r* values indicate Spearman’s rank correlation coefficient. (**G**) Predicted persistence score of the *Aim2* LT memory domain when its six CGs are iteratively removed in silico. (**H**) Mean CpG methylation per domain in ST (orange) and LT (red) memory domains at the indicated time points. Black lines trace median methylation rates across indicated domains for each time point. *n* = 2 biological replicates, with two or three mice pooled per biological replicate. Data represent one experiment per time point. (**I**) RPKM-normalized ETS1 Cut-and-Run signal ±2 kb from LT and ST memory domain centers at the height of acute inflammation (D6 IMQ). Signals were merged across biological replicates. *n* = 2 biological replicates, with two or three mice pooled per biological replicate. Data represent one experiment. (**J**) CpG methylation trajectories for individual CpG sites located within ELK/ETS motif (#5) instances that are ETS1-bound at D6 IMQ. Each point represents one CpG. (**K**) Robust association between PersistNet-predicted nucleotide contributions (top, green), in vivo CpG methylation profiles at D6 and Y1 (middle, black), and ETS1 binding during D6 IMQ within the LT domain of *Aim2*. See also figs. S7 to S9.

As the sequence features that drove memory establishment failed to discriminate longevity during inflammation, we asked whether a distinctive DNA sequence feature might instead act to enduringly control persistence. To this end, we developed PersistNet, a BPNet-based model (47) designed to predict not chromatin accessibility at a single point in time but rather its persistence across time, effectively learning which sequence features are most predictive of lasting accessibility rather than immediate opening (Fig. 5A, fig. S7A, and methods).

Deep learning models discern patterns that reflect the task they are trained on. To uncover what DNA features endow memory with persistence, our model had to be trained explicitly on a measure of persistence itself. We thus defined “persistence scores” as the per-base accessibility ratios of Y1 PIMQ over D30 PIMQ ATAC-seq signal in accessible chromatin domains across the entire genome (methods). Conceptually, this score captures how well each base of an accessible region remains open after 1 year of cellular turnover. Because accessibility is measured over the same genomic sequence at both time points, the ratio inherently normalizes for sequence composition and local assay bias, thus isolating the temporal component of stability rather than absolute accessibility.

Per-base persistence scores robustly distinguished long-from short-term memory domains [area under the curve (AUC) = 0.87] (Fig. 5A). We then trained a model to predict persistence score profiles from DNA sequence, deriving base-level contributions to persistence and ultimately de novo sequence motifs [~6 to 30 base pairs (bp)] associated with long-lasting chromatin accessibility (fig. S7A). The model accurately predicted experimentally observed accessibility persistence (Spearman’s correlation, *r* = 0.62; data S20 and fig. S7, B and C), revealing that longevity was, at least in part, an intrinsic property of sequence. This approach enabled us to assign per-base contribution scores and extract de novo sequence motifs that capture the DNA features most predictive of sustained chromatin accessibility through time.

We identified five de novo sequence patterns that were most predictive of chromatin accessibility persistence in postinflamed EpdSCs (Fig. 5B, fig. S7D, and data S22). These sequences were largely distinct from those uncovered by our earlier ChromBPNet analysis of memory establishment. Whereas ChromBPNet highlighted classical motifs for inflammation-induced TFs, PersistNet revealed a separate sequence vocabulary associated with longevity. Among the five sequences, one corresponded to the AP1 motif (motif #1), in line with the sustained binding of c-JUN observed in long-term memory domains through Y1 (Fig. 1F). By contrast, the remaining four sequences were either unassigned or resembled motifs of other TFs expressed during EpdSC homeostasis, including the ELK/ETS family (motif #5), NRF1 (motif #9), and SP1 (motif #18).

With the exception of ELK/ETS, none of these “persistence sequences” were predicted by ChromBPNet to differentially contribute to inflammation-induced accessibility of long-term memory domains. Even then, the PersistNet ELK/ETS motif differed markedly from its ChromBPNet counterpart, with a dominantly contributive CpG at its core and altered flanking symmetry. This motif was also predicted to have an outsized contribution to persistence in nearly every long-term memory domain, in contrast to its relatively modest contribution to establishment, where fewer than half of these loci carried a ELK/ETS-like site (compare Figs. 4C and 5B). This pronounced difference in both prevalence and predicted weight suggested that while PersistNet-predicted TFs may play a limited role in initiating accessibility, their sequence contexts are well suited to sustain it. Thus, the DNA sequences that encode memory persistence follow a distinct grammar from those governing its initial establishment, underscoring that epigenetic longevity is encoded in a distinctive regulatory syntax.

We next examined whether a common feature might unite these otherwise heterologous persistence motifs to support accessibility through time and cell division. Aside from the AP1 motif, each statistically significant persistence sequence contained at least one CpG dinucleotide that in every case corresponded to its highest base-level contribution to persistence. Indeed the most differential sequence (motif #4) did not match to any known TF; rather, its most notable feature was a well-defined CpG site with largely random flanks (Fig. 5B). Probing further, we found a conspicuous enrichment for CpG, but not any other dinucleotide combination, in long-over short-term memory domains (Fig. 5C) despite similar overall content of individual C and G nucleotides (fig. S7, E and F). This preeminence hinted at a unifying principle: that DNA sequence composition, rather than specific TF identity, could underlie the enduring accessibility of memory domains. Our observations pointed to CpG enrichment itself as a strong candidate for shaping long-term memory persistence, not only within canonical TF-bound contexts such as with ELK/ETS and NRF1 but also in unassigned sequence settings (fig. S7G).

If CpG content underlies memory persistence, it should scale with longevity across all 934 memory domains rather than distinguish only long- and short-term extremes. Consistently, CpG density correlated continuously with accessibility persistence: Higher CpG content predicted greater durability of the open state (Fig. 5D).

To directly test whether CpG content drives the persistence predicted by our model, we performed a series of in silico perturbations. Specifically, we examined whether the presence or absence of CpG dinucleotides within a chromatin domain could increase or reduce its predicted longevity. In silico introduction of CpGs into random DNA sequences was sufficient to raise predicted longevity—a boost not recapitulated by any other dinucleotide pair (Fig. 5, E and F, and fig. S7H). Conversely, iterative removal of the six CpG sites punctuating the *Aim2* long-term memory domain produced a progressive, stepwise decrease in predicted persistence, which scaled with CpG loss in a smooth, dose-dependent manner (Fig. 5G). These analyses suggested that CpG density alone is sufficient to tune the longevity of chromatin accessibility, operating as an intrinsic sequence feature that governs the stability of epigenetic memory.

### Memory persistence is partially supported by inflammation-triggered CpG demethylation

CpG methylation is fundamental to several imbricated aspects of chromatin structure, including nucleosome occupancy (48, 49), TF binding (50–52), and acquisition of variant histones (53). Of note, CpG methylation landscapes and their epigenetic consequences are typically propagated through cell divisions (54). We thus used whole-genome bisulfite sequencing (WGBS) to measure the levels of CpG methylation over the full IMQ timecourse (fig. S8, A and B). At naïve baseline, long-term memory domains were more methylated than their short-term counterparts, which were in turn more methylated than nonmemory regions that resolved by D30 (mean methylation rates: ~70, ~50, and ~35%, respectively) (Fig. 5H and data S24). Upon inflammation, however, all domains reached a comparably low methylation state (~25 to 30% mean methylation), reflecting a global demethylation that most prominently affected long-term memory domains (fig. S8, C and D).

Long-term memory domains were additionally exceptional in maintaining inflammation-induced demethylation, even years after return to normal homeostasis. By contrast, short-term and resolved domains gradually regained DNA methylation over time (Fig. 5H and fig. S8D). In line with these patterns, our persistence model revealed an anticorrelation between methylation status of a CpG site and its predicted contribution toward memory longevity (long-term: *r* = −0.21, *P* < 0.001; short-term: *r* = −0.09, *P* = 0.006) (fig. S8E).

The enduring demethylation of long-term memory domains was intriguing because the two most differential TF-matched persistence motifs resembled binding sites for the ELK/ETS family and NRF1, both of which strongly favor demethylated CpG (51, 55). Indeed, although ETS1 showed no occupancy at memory domains during naïve homeostasis, upon inflammation it bound robustly, concomitant with demethylation (Fig. 5I).

ETS1 binding was clearly detected at high-contribution ELK/ETS motif instances predicted by PersistNet, despite considerable sequence variability among these sites (fig. S9, A and B). These data confirmed that our model recovered genuine in vivo occupancy events despite flexible sequence grammar. When we examined CpG methylation over these sequences, we discovered that ELK/ETS motifs residing in long-term memory domains remained demethylated for life, whereas the others gradually regained baseline methylation levels (Fig. 5J and fig. S9C). Consistently, CpG instances across the top 1000 ELK/ETS memory domain motifs, as ranked by PersistNet contribution, displayed sustained hypomethylation over time, while low-contribution motifs were gradually remethylated (fig. S9D). Finally, using our paradigm long-term memory gene, we found that DNA methylation over *Aim2* was enduringly reduced at the exact bases where predicted contributions to persistence were the highest, which included an inflammation-induced binding site for ETS1 (Fig. 5K).

These data suggested that in sustaining inflammation-induced demethylation, long-term memory domains were able to retain an amenable binding landscape for key persistence-defining TFs. However, we were still left with a paradox as to how demethylation was selectively sustained across long-term memory domains and, more broadly, how these changes translated to inheritable chromatin accessibility.

### The histone variant H2A.Z integrates DNA sequence, methylation, and accessibility to control longevity of inflammatory memory

Nucleosome positioning is strongly influenced by DNA sequence, preferring alternating 10-bp periodicities of AA/TT/AT/TA and GC/CG dinucleotide motifs (56, 57). Given the high CpG content and methylation dynamics of long-term memory domains, we hypothesized that these sites may harbor DNA sequences that support unstable nucleosome architectures.

**Fig. 6.**
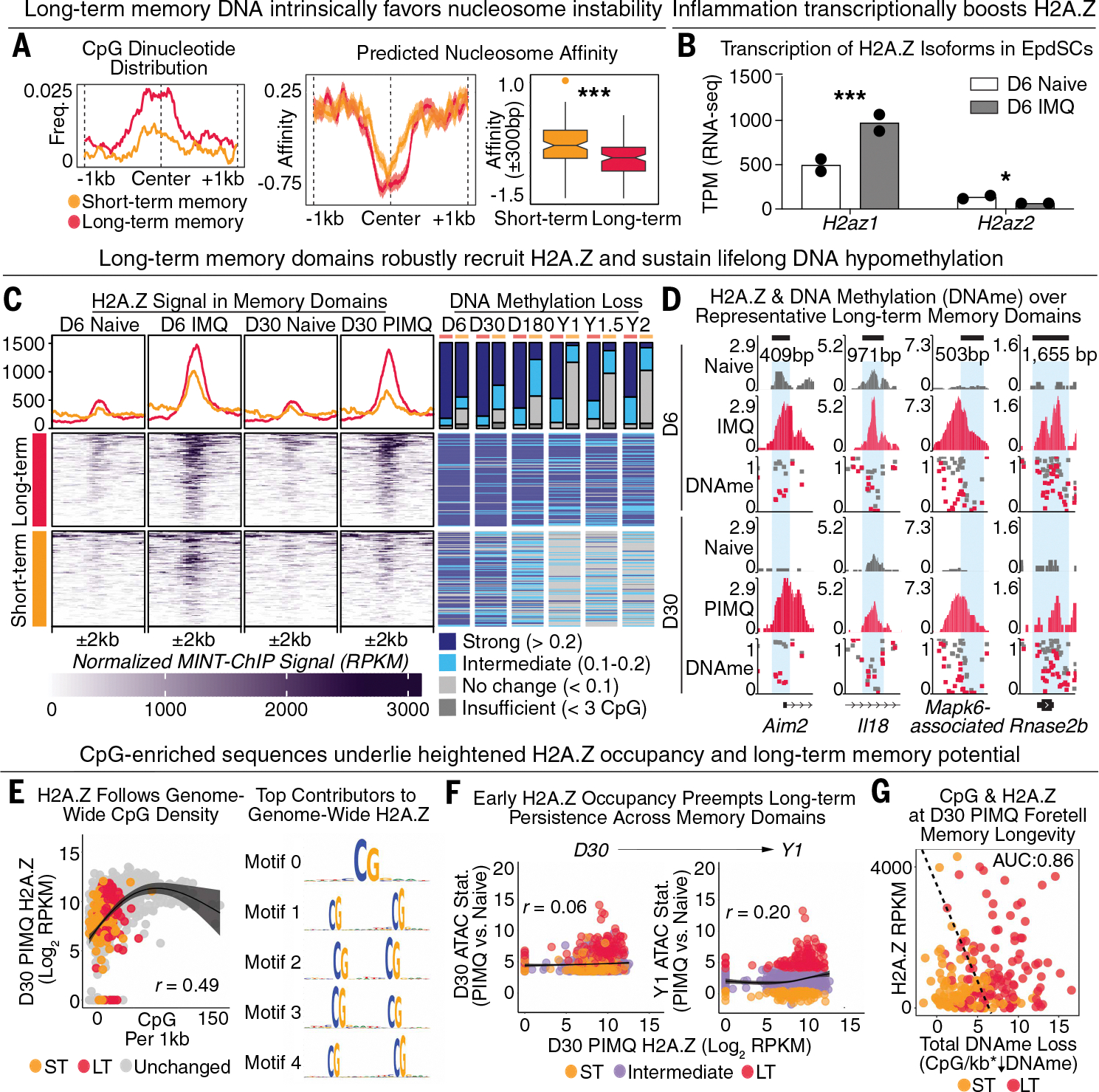
CpG-enriched loci recruit H2A.Z to demarcate lifelong durability in newly accessible inflammatory memory domains. (**A**) Predicted nucleosome affinities for memory domain sequences. (Left) Frequency of CpG dinucleotides within and flanking ST and LT memory domains. (Middle) Predicted nucleosome binding affinities for memory domain sequences ±2 kb from ATAC peak centers, determined using NuPoP (59). Line thicknesses indicate standard errors of prediction. (Right) Box and whisker plots of nucleosome affinities per ST or LT domain ±300 bp of ATAC peak centers. ***P < 0.001 (two-tailed unpaired Student’s *t* test). (**B**) Transcripts per million of H2A.Z isoforms from bulk RNA-seq of EpdSCs at D6 naïve and D6 IMQ (4). Each point represents a biological replicate. ***P-adj < 0.001; *P-adj = 0.030 (DESeq2 Wald test). (**C**) (Left) Heatmaps of RPKM-normalized H2A.Z MINT-ChIP signal ±2 kb from LT and ST domain centers at times indicated. (Right) Corresponding DNA methylation dynamics after inflammation. *n* = 2 biological replicates per condition, with two or three mice pooled per replicate. Data represent one experiment per time point. (**D**) Signal tracks of H2A.Z (RPKM-normalized MINT-ChIP) and mean CpG methylation (DNAme) profiles of representative LT memory domains. Blue shaded boxes indicate domain boundaries as determined by ATAC-seq. Note persisting H2A.Z and CpG demethylation PIMQ. (**E**) (Left) Scatterplots showing H2A.Z versus CpG density in ST memory, LT memory, and unchanged domains of D30 PIMQ EpdSCs. Each point represents one domain. Black lines show smoothed trends fitted by a generalized additive model, with gray ribbon indicating standard error. *r* values indicate Spearman’s rank correlation. (Right) Contribution weight matrices of top de novo sequence motifs predicted to contribute to genome-wide H2A.Z deposition in D30 PIMQ EpdSCs, as identified by a BPNet model trained on H2A.Z MINT-ChIP signal over accessible chromatin (see methods). The height of each nucleotide represents its relative contribution to H2A.Z signal. (**F**) Scatterplots relating H2A.Z signal and chromatin accessibility (DESeq2 Wald statistic of PIMQ over naïve signal) across all 934 memory domains at D30 (early resolution) and Y1 (late resolution). Each point represents a memory domain. Black lines show smoothed signals across plotted domains using a generalized additive model, with gray ribbons indicating standard error. *r* values indicate Spearman’s rank correlation coefficients. Note that similar to CpG density (Fig. 5D), H2A.Z levels at D30 show little correlation with ongoing accessibility but align more strongly with future memory persistence. (**G**) Scatterplot illustrating the combined predictive power of H2A.Z and total loss of DNA methylation (CpG density of each domain multiplied by its reduction in mean DNA methylation at D30 PIMQ) to distinguish LT from ST memories at D30 PIMQ, when accessibilities are overall comparable. Dashed line marks the decision boundary derived from logistic regression, showing that these features form a signature to discern memory longevity shortly after inflammatory resolution. AUC, area under the curve. See also fig. S10.

We used a hidden Markov model trained on highly sensitive chemical maps of mouse nucleosomes to predict the intrinsic “nucleosome affinities” of inflammation-responsive or unchanged DNA sequences (57, 58). All sequences were predicted to have low nucleosome affinities, consistent with their euchromatic state and potential for chromatin openness. However, long-term memory sequences displayed a distinctly lower and broader “valley” of predicted nucleosome disaffinity versus all other domains, which closely matched their zone of CpG enrichment (Fig. 6A and fig. S10, A to C) (59). We next performed in silico predictions of tertiary DNA conformation, integrating observed methylation status from our in vivo bisulfite sequencing. These data suggested that long-term domains preferred to relax negatively angled planar rotations (“propeller twists”) upon inflammation-induced DNA demethylation, consistent with a potentially increased DNA flexibility and ability to unwrap from nucleosomes. This putative reshaping was predicted to remain uniform over 2 years (fig. S10D) (57, 60). Together, these analyses indicated that long-term memory domains may harbor sequence preferences that favor unstable nucleosome structures, which are enduringly supported by inflammation-induced DNA demethylation.

The collective epigenetic and genetic signatures of long-term memory domains bore close resemblance to genomic conditions favoring the histone H2A.Z, which was also transcriptionally up-regulated in EpdSCs upon skin exposure to IMQ (Fig. 6B). H2A.Z is a conserved variant of canonical H2A that differs in its C-terminal tail to engender active epigenetic states marked by nucleosome instability (61, 62), chromatin accessibility, and DNA unwrapping (63–65). H2A.Z-associated nucleosomes are also mutually antagonistic with DNA methylation (53, 65–67), suggesting that they may preferentially occupy CpG-dense domains sensitive to this modification and help stabilize a demethylated state once established (67, 68). Finally, H2A.Z synthesis and incorporation are independent of cell division and can be rapidly restored after DNA replication (67, 69), supporting a potential contribution to propagation of epigenetic states through cell division.

We monitored H2A.Z nucleosome occupancy through acute inflammation and 1 month of resolution. In line with closed chromatin conformations, H2A.Z was largely absent from memory domains in naïve EpdSCs. Upon inflammation, however, and consistent with the concomitant drop in DNA methylation, H2A.Z was robustly gained across memory domains with a notable preference for nearly all long-term regions (Fig. 6, C to D, and fig. S10, E and F). After resolution, H2A.Z persisted at long-term domains with minimal reduction in inflammation-endowed levels from D6 to D30. By contrast, short-term memory domains failed to retain H2A.Z, nearly returning to baseline levels by D30.

Our earlier computational analyses predicted that CpG enrichment privileges long-term memory domains for persistence but not initial accessibility. Accordingly, we found that differential binding of H2A.Z to CpG-enriched long-term memory domains was not a feature of chromatin accessibility itself, as by D30 all memory domains were still comparable in openness. Although H3K27ac has been suggested to promote H2A.Z deposition (70), this association was not obvious within memory domains (fig. S10G; compare with Fig. 6C). Rather, H2A.Z association appeared to foretell which memories would be retained and which were destined to be lost.

We next asked whether the sequence features of long-term memory domains allow these inflammation-dependent loci, once made accessible, to be brought into a larger fold of homeostatically accessible domains that readily acquire H2A.Z. As expected, H2A.Z robustly anticorrelated with DNA methylation in postinflamed homeostasis at D30 PIMQ, both genome-wide and across inflammation-induced memory domains (fig. S10H). Despite these shared dynamics, long-term memory domains consistently harbored more H2A.Z than their short-term counterparts, even though both were similarly demethylated at this time. We thus turned to whether CpG density itself may further influence H2A.Z deposition.

Genome-wide, H2A.Z occupancy increased from low to moderate CpG densities, generally corresponding to enhancers (~0 to 50 CpG/kb) (Fig. 6E). This dynamic range was in contrast to saturated H2A.Z-containing nucleosomes in high CpG density gene promoters (>50 CpG/kb). Reflecting these genome-wide patterns, inflammation-induced memory domains fell along an expected trendline of H2A.Z levels during postinflamed homeostasis (D30 PIMQ), with many long-term memory domains surpassing CpG densities that favored optimal H2A.Z retention. In support of this association, BPNet modeling of genome-wide H2A.Z 1 month after inflammation (D30 PIMQ) suggested that CpG dinucleotides were the dominant sequence feature underlying the presence of local H2A.Z (Fig. 6E).

These connections reflected a tunable signature underlying memory longevity. Following all 934 memory domains through time, we found that the extent of H2A.Z retention at D30 PIMQ mirrored sequence-intrinsic CpG density and continuously scaled with persistence at Y1 (Fig. 6F). Consistently, a joint signature comprising CpG density, DNA demethylation, and H2A.Z acquisition distinguished which memories would endure and which would erode even at just 1 month after inflammation, when all adaptations bore comparable levels of chromatin openness (Fig. 6G; AUC = 0.86). Together, these findings suggested that H2A.Z has genome-wide bias toward CpG-enriched, demethylated DNA and that passage of this organizing principle to stress-activated loci privileges a subset to persist in a poised, accessible state for a lifetime of cellular generations.

Finally, we posited that these mechanisms of memory longevity might apply to other cell types and inflammatory contexts. In agreement, in mining publicly available datasets, we found that CpG was enriched in the long-lasting epigenetic memories formed upon response of EpdSCs to wounding (71), acinar cells to pancreatitis (8), and hematopoietic stem cells to lipopolysaccharide exposure (13) (fig. S11). The extent to which these signatures are analogously marked by H2A.Z, an undermethylated state, and/or methylation-sensitive TFs awaits future study as does the extent to which these features apply to additional cell types, stimuli, and species.

## Discussion

Nonspecific memories of inflammatory experiences can persist for years after infection (72), vaccination (73), and injury (11), through mechanisms long elusive. We found that epigenetic memories in EpdSCs can last a lifetime after acute exposure of the skin to inflammation. The chromatin regions harboring this memory remained largely closed in naïve aged mice, indicating that their lifelong persistence was not attributable to aging itself. Rather, our analyses suggested that a sequence-intrinsic mechanism fine-tunes the rates at which individual inflammatory memories, once established by stress-induced TFs such as pSTAT3 and AP1, persist across time and cellular generations even years after an activating encounter.

Using nucleotide-resolution deep learning approaches, we unearthed CpG density as the principal driver of this longevity-defining syntax, which was distinct from the AP1-dominated sequence grammar that governs initial memory establishment (22). We showed that CpG-enriched long-term memory domains became robustly demethylated during inflammation, harbored functional binding sites for DNA methylation–sensitive homeostatic TFs (e.g., ETS1), had an intrinsic predicted nucleosome disaffinity, and readily incorporated the nucleosome-destabilizing histone variant H2A.Z.

As CpG-mediated chromatin features and H2A.Z are found in nearly all eukaryotes, it is tempting to speculate that mechanisms of memory longevity that we unearthed here represent an ancient, conserved form of stress adaptation. Indeed, H2A.Z regulates the homeostatic expression of several effectors of infectious memory in plants (74), which lack a classical immune system. H2A.Z has also been proposed to poise environmentally sensitive genes in yeast by constructing unstable, or “fragile,” nucleosomes (58). The direct consequences of these activities toward chromatin-harbored memory and secondary stress have yet to be explored but may offer an evolutionary basis for the observed longevity and potential ubiquity of many nonspecific memories in higher-order species, especially given the availability and indispensability of these factors to most cells. To this end, our reanalysis of published datasets suggests that CpG density roots memory longevity in diverse murine cell types, including EpdSCs, acinar cells, and hematopoietic stem cells.

The use of a conserved, CpG-dependent syntax to intrinsically mark certain cis-regulatory domains for long-term adaptation begins to explain puzzling observations as to why heterologous infectious resistance or recurrence of chronic diseases can last for years, often in the absence of enduring inflammation-induced changes to the tissue microenvironment. Moreover, our insights into the mechanisms underlying memory persistence suggest therapeutic axes to enduringly enhance the protective benefits of inflammatory memory while suppressing its most persisting downsides, such as chronic inflammation and cancer.

## Materials and methods

### Animals

C57BL/6J female mice were purchased from the Jackson Laboratory at 6-weeks of age then housed in specific pathogen–free conditions at the Association for Assessment and Accreditation of Laboratory Animal Care International (AAALAC)–accredited Comparative Bioscience Center at the Rockefeller University. All procedures were performed with approval from the Institutional Animal Care and Use Committee (IACUC) (protocol number 23038-H). Male mice were not used to avoid effects of fighting. Aging animals that developed spontaneous inflammatory skin pathologies with age (e.g., ulcerative dermatitis, alopecia) were additionally excluded. All animals were housed in accordance with the procedures delineated in the Guide for the Care and Use of Laboratory Animals. Mice were maintained under a 12-hour light/dark cycle and were provided with food and water ad libitum. Animals were randomly assigned to experimental groups and euthanized with CO_2_ prior to experimental assays.

### Imiquimod treatment

As previously described, the back skin of 8-week-old mice was shaved and subjected to topical application of ~1 mg cm^2^ 5% IMQ cream (Perrigo) for six consecutive days (4).

### Histology

Skin was fixed with 10% formalin in phosphate-buffered saline (PBS), washed three times for 15 min with PBS, and stored in 70% ethanol. Samples were paraffin embedded, sectioned (0.8 mm) and stained with hematoxylin and eosin (H&E) by Histowiz Inc.

### Punch biopsy wounds

Dorsal back skin of Y1 (naïve/PIMQ) mice was shaved, and visual assessment was performed to ensure the wounding area was in telogen (resting phase of the hair cycle). Mice were anesthetized with isoflurane and buprenex (0.1 mg/kg) administered. Four-millimeter biopsy punches (Miltex) were used to make full thickness wounds. Six to nine mice were used per condition, with four wounds per mouse being used as technical replicates. Area of the wounds was measured daily for seven consecutive days.

### TPA administration

12-*O*-Tetradecanoylphorbol-13-acetate (TPA) was diluted to 0.125 mg/ml in ethanol and topically applied to the shaved backs of mice. EpdSCs were FACS (fluorescence-activated cell sorting) purified 6 hours after treatment for RNA-seq and CNR.

### Epidermal thickness quantifications

Epidermal thickness was determined by KRT14 (basal layer of epidermis) and KRT10 (differentiated payer of epidermis) immunofluorescence. Murine back skin was fixed for 20 min at room temperature (RT) in 4% paraformaldehyde in PBS. Tissue was washed three times in PBS and switched to 20% sucrose overnight at 4°C. To remove sucrose, tissue was washed three times in PBS and embedded sagittally in optimal cutting temperature compound, frozen on dry ice, and cryo-sectioned at 14 μm. Tissue sections were then permeabilized with 0.3% Triton X-100 in PBS for 20 min at RT, blocked, stained with primary antibodies (overnight at 4°C), washed, and stained with secondary fluorescence conjugated antibodies (1 hour at RT). Nuclei were stained with 4′,6-diamidino-2-phenylindole (DAPI). Images were taken with an AxioObserver.Z1 epifluorescence microscope and images processed and analyzed with ImageJ software.

### EdU quantifications

EdU (5′-ethynyl-2′-deoxyuridine) was injected in Y1 (naïve/PIMQ) mice intraperitoneally (50 μg/g) (Sigma-Aldrich) 1 hour before analysis. Immunofluorescence was performed as described above, with EdU staining done with the Click-iT EdU Cell Proliferation Kit for imaging by following manufacturer’s instructions. Proliferation was calculated as percent of EdU-positive DAPI-stained nuclei of total DAPI nuclei within the basal layer of the epidermis as marked by positive KRT14 staining.

### Cell isolation and tissue processing

Interfollicular EpdSCs were isolated as previously described (22). Briefly, back skin of mice was shaved, harvested, and digested using 0.25% trypsin/EDTA (GIBCO) at 37°C for 40 min, followed by scrapping to obtain a single-cell suspension. Cells were filtered through 70- and 40-μm filters and then stained with antibodies (table S1) in 200 μl FACS buffer (PBS contained 5% fetal bovine serum and 1% HEPES). EpdSCs were FACS isolated on positive expression of CD49f, Sca-1, and CD29. Lineage negative dump included CD31, CD45, CD117, and CD140a.

### Skin immune cell characterization

Skin immune cells were isolated from 1 cm^2^ pieces of back skin as previously described (7). Briefly, skin was minced then digested with 0.5 mg/ml Liberase TL (Millipore Sigma) at 37°C for 2 hours, followed by filtering through 70- then 40-μm filters. Samples were stained for 20 min in cold PBS using LIVE/DEAD Fixable Blue (Invitrogen), then for 30 min in FACS buffer for adaptive immune cell markers using antibodies listed in table S1. Cells were intracellularly stained using Foxp3/Transcription Factor Staining Kit (ThermoFisher) for cell fixation and permeabilization and using antibodies listed in table S1.

### ATAC-seq library preparation and sequencing

ATAC-seq was performed on 100,000 FACS-purified cells as previously described (75). Briefly, cells were lysed in ATAC lysis buffer for 5 min, then treated with Tn5 transposase (Illumina) for 30 min. Sequencing libraries were prepared according to manufacturer guidelines (Illumina), with up to eight uniquely barcoded samples included per sequencing run. Libraries were sequenced on an Illumina NextSeq500 or NovaSeq 6000. FASTQ files for D6 and D30 can be accessed from GSE171596 (22). At least two biological replicates were sequenced per condition, with 2 or 3 mice pooled per biological replicate.

### ATAC-seq preprocessing

Fifty–base pair paired-end FASTQ files were aligned to the mm10 genome from the GENCODE (v.30) (76) database using BWA-mem (77) with parameters -M -T 10 in paired-end mode. All BAM files showed >90% mapping rate. BAM files were filtered using samtools (78) -F 1804 -f 2 -q 30 to remove unmapped reads, secondary alignments, optical duplicates, and reads that failed platform or vendor quality checks or had low mapping quality. Polymerase chain reaction (PCR) duplicates were identified and marked by P icard (http://broadinstitute.github.io/picard) and removed using Samtools. BAM files were filtered to only consider alignments mapping from Chr 1 to 19 and ChrX. Other alignments mapping to scaffolds or blacklist (downloaded from https://mitra.stanford.edu/kundaje/akundaje/release/blacklists/mm10-mouse/mm10.blacklist.bed.gz) were removed using bedtools intersect. Normalized bigWig files were calculated using the coverage function from the rtracklayer package and normalized by counts per million (CPM). Deeptools (79) bamcompare was used to generate log2 ratio bigWigs of postinflamed versus control conditions.

Forward and reverse reads from BAM files were separated to generate TAG files in BED format. Offsets were adjusted such that reads mapped to the plus strand were shifted by +4, while reads mapped to the reverse strand were shifted by −5. Peaks were called for each replicate with MACS3 (80) using as input the TAG files with the parameters -f BED –nomodel –shift -75 –extsize 150 -g 2652783500 (mouse genome length in base pairs).

Differential accessibility analysis was performed using the DESeq2 R package (v1.42.1) (81). Briefly, consensus peak sets were first generated by requiring that a MACS3 peak was called in at least all but one replicate per condition given more than two replicates, or in both replicates if only two replicates were available. Reproducible peaks per condition were then merged to form a nonredundant consensus set of 220,884 peaks used for differential analysis, after which counts per peak were determined using the summarizeOverlaps() function from the GenomicAlignments V1.44.0 package. Raw counts across all conditions were imported into a DESeq2 object using a standard design model, and principal components analysis (PCA) performed using the pca() function in the DESeq2 package.

### Selection of short- and long-term memory domains from ATAC-seq

To define cis-regulatory domains of interest, the DESeq2 object was subset to only include D6 (inflamed), D30 (short-term resolution), and Y1 (long-term resolution) time points owing to consistent replicate number for each time point and condition (*n* = 4) and stabilization of aggregate memory domain accessibility by Y1. Differential analyses were then performed between inflamed/postinflamed and naïve conditions for each time point, using the DESeq2 default Wald test (*P-adj* < 0.05, log_2_ fold change > 0 used as significance thresholds for all comparisons). Memory domains were defined as loci having heightened accessibility in both D30 PIMQ over D30 naïve (979 peaks) and D6 IMQ over D6 naïve (75,642 peaks) comparisons (final overlap *n* = 934 peaks). Long-term memory domains were defined as peaks with heightened accessibility in Y1 PIMQ over Y1 naïve (131 peaks), which also belonged to the initial 934 memory peaks identified at D30 (final overlap 117 peaks). Short-term memory domains were then defined as the 117 peaks of the initial 934 memory domains having the Wald statistic value closest to 0 in the Y1 PIMQ versus Y1 naïve comparison. Resolved domains were defined as 934 loci having heightened accessibility in D6 IMQ over D6 naïve, then having the Wald statistic closest to 0 in the D30 PIMQ versus D30 naïve comparison. Unchanged domains were first identified as peaks that did not change accessibility in any comparison between naïve conditions at D6, D30, or Y1 nor in any comparison between P/IMQ and naïve for any time point (*P-adj* > 0.2; 16,897 peaks). Nine hundred thirty-four peaks were randomly sampled from these domains to derive a final set of unchanged peaks for downstream analysis. To generate profile plots and heatmaps, we used deepTools v3.5.6 with the following procedure. First, bigWig files were generated from merged BAM files using bamCoverage with parameters–binSize 1 –normalizeUsing RPKM –extendReads. Next, computeMatrix was applied in scale-regions mode with parameters –regionBodyLength 500 -binSize 1. Finally, plotProfile and plotHeatmap functions were used with default parameters to generate the corresponding visualizations.

### Memory domain–associated genes and gene scores from ATAC-seq

To associate memory domains with their putative target genes, we performed proximity-based gene annotation using the mouse mm10 genome assembly. Transcript and gene body coordinates were obtained from the TxDb.Mmusculus.UCSC.mm10.knownGene R package, and transcription start sites (TSSs) were defined for all transcripts using the promoters() function. For each ATAC-seq memory region, we identified the nearest TSS using the distanceToNearest() function, calculating the genomic distance between the peak and the closest TSS in a strand-aware manner. Gene symbols were mapped from Entrez IDs using the org.Mm.eg.db annotation package. The resulting annotation table included peak coordinates (chromosome, start, end), nearest gene information (Entrez ID, gene symbol, TSS strand, distance in base pairs), and gene body coordinates (chromosome, start, end, strand) for each memory domain. To quantify chromatin accessibility at the gene level, we adapted the gene scoring approach from ArchR (44) and implemented a custom calculateBulkGeneScores() function. This method takes as input the continuous ATAC-seq signal from cut sites BigWig files across gene-centric regions, applying a distance-weighted scoring scheme. For each gene, a plateau region was defined extending from 5 kb upstream of the TSS to the transcription termination site (TTS), where maximal weight (1 + e^−1^ ≈ 1.368) was assigned. Signal outside this plateau, extending up to 100 kb upstream and downstream, was weighted using exponential decay [ê(−distance/5000)]. Gene boundaries were respected to prevent signal attribution from neighboring genes. Raw gene scores were normalized using reads per million (RPM) to account for sequencing depth differences across samples, enabling direct comparison of chromatin accessibility across biological replicates and conditions. Lastly, gene scores were standardized by calculating *z*-scores across all eight replicates (four Y1 Ctrl and four Y1 PIMQ, or four D30 Ctrl and four D30 PIMQ) to facilitate comparison of chromatin accessibility between conditions.

### Classical motif scanning using MotifMatchR

Motif occurrence was quantified by scanning sets of genomic regions (short-term and long-term) against two independent transcription factor binding site databases to ensure robustness of findings. The primary analysis used a curated collection of 884 mouse transcription factor position-weight matrices (PWMs) derived from Cis-BP (45) (mouse_pwms_v2), while parallel analyses were conducted using 746 vertebrate TF motifs from the JASPAR2020 CORE collection (82). Both analyses were performed against the mm10 reference genome. Motif matching was performed using the matchMotifs function from the motifmatchr package (83) with the output parameter set to “positions” to retrieve the genomic coordinates of all motif matches. For each PWM in both databases, we recorded all matching genomic positions and then quantified the number of motif hits overlapping each region using the countOverlaps function from the GenomicRanges package, producing region × motif tables in which each row corresponds to a specific region–motif pair and includes the region identifier, motif ID, associated TF name, and the raw number of hits (i.e., motif occurrences) per region. To identify motifs differentially represented between classes, TFs were defined by their motif annotations, and, for each TF, the distributions of per-region motif counts in short-term versus long-term sets were compared using a two-sample unpaired Wilcoxon test. *P* values were adjusted for multiple testing across all tested TFs using the Benjamini–Hochberg procedure and a nominal threshold of adjusted *P* value < 0.05 was used to flag significance.

### Sample preparation and processing for scATAC-seq

D30: EpdSCs (CD49f^+^, Sca-1^+^, CD29^+^, CD34^−^, CD45^−^, CD117^−^, CD140a^−^, CD31^−^) and immune cells (CD45^+^, Ly6G^−^) were FACS isolated and mixed at a 1:1 ratio to perform 10x Chromium Next GEM single cell multiome ATAC + Gene expression following 10x protocol for nuclei extraction. Downstream processing of extracted nuclei was performed by the Integrated Genomics Operation (IGO) at Memorial Sloan Kettering Cancer Center. Downstream processing was only performed on scATAC-seq data.

Y1: EpdSCs (CD49f^+^, Sca-1^+^, CD29^+^, CD34^−^, CD45^−^, CD117^−^, CD140a^−^, CD31^−^) were FACS isolated and nuclei extracted using 10x protocol for nuclei extraction. Downstream processing of extracted nuclei was performed by the Integrated Genomics Operation (IGO) at Memorial Sloan Kettering Cancer Center where single cell ATAC-seq was performed.

### scATAC-seq alignment for D30 and Y1

Raw scATAC-seq reads were aligned using CellRanger ATAC (v2.0) to generate fragment files, and data processing was performed with ArchR (v1.0.2). Arrow files were constructed using default parameters, including gene score and tile matrices. Only fragments shorter than 600 bp were considered for all scATAC-se preprocessing steps and downstream analyses.

### scATAC-seq quality control and preprocessing (D30)

Preprocessing of the D30 scATAC-seq dataset was carried out using ArchR (v1.0.2). Fragment files generated by CellRanger ATAC were used to construct Arrow files containing both TileMatrix and GeneScoreMatrix representations. To ensure data quality, cells were filtered on the basis of three criteria: a minimum of 2000 fragments per cell, a TSS enrichment score greater than 5.5, and exclusion of cells identified as doublets. Doublet detection was performed using ArchR’s addDoubletScores() followed by filterDoublets(). In addition, immune cells were removed on the basis of high PTPRC gene scores, resulting in a filtered dataset comprising 11,034 high-quality epithelial cells, with 5399 from the control group and 5635 from the PIMQ group. Dimensionality reduction was performed using iterative latent semantic indexing (LSI) applied to the TileMatrix. The LSI procedure involved three iterations, using 20,000 variable features and retaining the top 30 principal components. Clustering was carried out using the Seurat method with a resolution of 0.2. A two-dimensional UMAP (uniform manifold approximation and projection) embedding was then computed using cosine distance with 30 neighbors and a minimum distance of 0.5. All filtering thresholds and dimensionality reduction parameters were selected on the basis of diagnostic visualizations and gene score patterns, ensuring robust and reproducible separation of epithelial subpopulations for downstream analysis.

### scATAC-seq quality control and preprocessing (Y1)

The Y1 dataset underwent the same preprocessing pipeline in ArchR, with adjusted quality control parameters to account for differences in sequencing depth and signal characteristics. Arrow files were generated with TileMatrix and GeneScoreMatrix for each sample. Cells were retained if they showed a TSS enrichment score of at least 13, had a minimum of 12,000 fragments, and were not classified as doublets by the addDoubletScores() and filterDoublets() functions, using UMAP as the neighborhood graph for doublet detection. These thresholds were selected on the basis of manual inspection of UMAP projections colored by fragment counts and gene activity, allowing the selection of dense clusters representing high-confidence epithelial cells. After filtering, the final dataset included 4476 epithelial cells, composed of 2181 from the control group and 2295 from the PIMQ group. Dimensionality reduction was performed using iterative LSI with three iterations and 20,000 variable features, retaining the top 30 components. Cells were clustered using the Seurat method at a resolution of 0.2, and UMAP was computed using 30 neighbors, a cosine distance metric, and a minimum distance of 0.5. To further assess the robustness of the low-dimensional embedding, a second round of LSI was performed using an expanded feature set of 30,000 tiles, followed by reclustering and UMAP recalculation. Visual validation of cluster structure, fragment enrichment, and gene score patterns confirmed the stability and reproducibility of the embedding and ensured the integrity of the filtered dataset for subsequent analyses.

### Peak calling from scATAC-seq D30 and Y1

To define a reproducible peak set across cells, we used Macs3 through ArchR to perform peak calling independently for each dataset (D30 and Y1). First, pseudo-bulk replicates were generated by grouping cells by condition using addGroupCoverages function, restricting fragment lengths to a maximum of 600 bp to enrich for nucleosome-free regions. Peak calling was then performed using addReprodicublePeakset function with default parameters. The resulting peak set was used to construct a binary peak-by-cell accessibility matrix, again restricting fragments to a maximum length of 600 bp. This resulted in 112,211 and 164,945 peaks for D30 and Y1 scATAC-seq, respectively.

### SEACell calling from scATAC-seq

To address the sparsity of scATAC-seq data, we applied SEACell (42) independently to the control and PIMQ datasets for both D30 and Y1. Inputs included de novo peak-by-cell accessibility matrices (PeakMatrix), reduced-dimensional embeddings from iterative LSI, and gene score matrices exported from ArchR. The kernel for SEACell inference was constructed using the LSI representation (X_svd). The number of SEACells was determined heuristically at approximately one SEACell per ~75 cells. A neighborhood graph was initialized using the top 10 way-point eigenvectors, and metacell fitting was performed iteratively (up to 500 iterations) until convergence. Initialization and convergence diagnostics were visually inspected to ensure that SEACell archetypes were well distributed across the phenotypic space and that the model had stabilized. Both hard (each cell assigned to a single SEACell) and soft assignments (weight distribution across SEACells per cell) were computed. The weight distribution was analyzed to confirm that most cells had one to four SEACells with nontrivial contributions (weight > 0.1), indicating a sparse and interpretable clustering. The strength of the top five assignment weights per cell was also examined. SEACell purity was evaluated by comparing assignments to preexisting ArchR clusters, revealing that most SEACells were predominantly composed of a single cell type, consistent with biological coherence.

### SEACell normalization and cell type annotation

After SEACell inference, peak accessibility matrices for control and PIMQ were aggregated independently using hard SEACell assignments, resulting in metacell-level AnnData objects (control_meta_ad, pimq_meta_ad). For each SEACell, we computed average coordinates in the LSI and UMAP spaces on the basis of constituent single-cell embeddings, enabling consistent integration across conditions in latent space. Gene activity scores were also aggregated across cells per SEACell. Although gene scores from scATAC-seq are not strictly quantitative, their relative enrichment patterns helped in assessing SEACell identity at the cell-type level. The control and PIMQ SEACell data were then merged into a unified object (ad_norm). All 112,211 (for D30 dataset) and 164,945 (for Y1 dataset) peaks were retained as each was detected in at least one SEACell. Library size normalization was performed using sc.pp.normalize_total(), followed by log-transformation with sc.pp.log1p() for both peak accessibility and gene score matrices. UMAP embeddings were recomputed from the singular value decomposition (SVD) representation, and clustering was performed using the Leiden algorithm (resolution = 1). Known epithelial lineage markers (Krt5, Krt14, Krt1, Krt10, Sox9) were visualized alongside condition and cluster labels to assess metacell composition and detect condition-specific shifts. On the basis of these marker gene patterns and cluster-level expression, SEACells clusters were annotated as cell types: clusters 1 to 4 were labeled as undifferentiated basal (UB), clusters 5 and 6 as differentiating basal (DB), and clusters 0 and 7 as upper hair follicle (uHF). Cell type annotation was performed manually by visualizing gene activity levels of canonical epidermal lineage markers (*Krt5*, *Krt14*, *Krt1*, *Krt10*, and *Sox9*) across SEACells and clusters. Annotations were based on known marker combinations characteristic of major epidermal states: UB SEACells were defined by high accessibility near *Krt5* and *Krt14* loci, reflecting chromatin openness typical of basal EpdSCs. DB SEACells displayed increased accessibility at *Krt1* and *Krt10* regions, consistent with cells initiating the differentiation process but still showing stemness markers (*Krt14*, *Krt5*). uHF SEACells were annotated on the basis of strong accessibility at the *Sox9* locus, marking hair follicle–associated stem cells.

### Force-directed layout visualization of SEACells

To visualize the global architecture of scATAC-seq data at the SEACell level, we computed a PAGA-guided FDL separately for each experimental time point (D30 and Y1). First, SEACell-aggregated AnnData objects from control and PIMQ conditions were concatenated. Then, a *k*-nearest neighbor (k-NN) graph (*k* = 15, cosine metric) was computed in the latent SVD space ('X_svd') using sc.pp.neighbors from Scanpy (84). Connectivity between SEACells was quantified using sc.tl.paga with the SEACell labels as the grouping variable (groups='SEACell') and the model set to 'v1.2'. Partition-based graph abstraction (PAGA) coordinates were initialized by invoking sc.pl.paga without edge thresholding, which stores the node layout in ad.uns['paga']['pos']. To generate the FDL, we used sc.tl.draw_graph(ad2, layout='fa', init_pos='paga'), which initializes the ForceAtlas2 layout using the PAGA coordinates. This approach improves convergence and preserves the global graph topology inferred by PAGA. To enable simultaneous visualization of SEACell and single-cell structure, we overlaid SEACell centroids onto the same FDL embedding. These centroids were computed as the average of the ForceAtlas2 coordinates (FA1, FA2) of all constituent single cells assigned to each SEACell.

### chromVAR analyses at the SEACell level

To quantify transcription-factor (TF) activity at single-SEACell resolution, we analyzed our scATAC-seq data with chromVAR v1.30.1 (43). We began with a peak-by-SEACell count matrix containing the 220,884 nonredundant peaks identified in the bulk ATAC-seq workflow (see “ATAC-seq preprocessing”) across SEACells from D30 PIMQ and Y1 PIMQ. Peaks with zero fragments in all SEACells were discarded. chromVAR first normalized fragment counts for GCcontent bias using the mm10 reference genome and then scanned every retained peak for 884 mouse PWMs from the mouse_pwms_v2 collection (chromVARmotifs). For each TF motif in each SEACell, it returned (i) a deviation *z*-score, which measures whether the motif’s binding sites are more or less accessible than expected given sequencing depth and GC composition, and (ii) a variability statistic that summarizes dispersion across SEACells. Deviation matrices were then averaged within each of the six condition–cell-type groups (two conditions: naïve and PIMQ; three cell types: UB, DB, and uHF) to yield a matrix of 884 motifs × 6 columns for each time-point object (D30 and Y1). For every motif, we computed Δ-scores for each cell type —for example, D30Δ_UB = D30PIMQ_UB – D30Naïve_UB—to capture the accessibility changes induced by inflammation that are retained during resolution in each population. Δ-scores from the two time points were merged and displayed in a density-weighted scatterplot (*x* axis: Day 30 Δ_UB; Y-axis: Year 1 Δ_UB). A dashed square spanning ± 5 Z-units delineates motifs whose accessibility remains essentially unchanged; statistical significance was determined with a Bonferroni-corrected *P-adj* < 0.01 to account for the 884 parallel hypothesis tests.

### Memory domain accessibility at the SEACell level

To quantify chromatin accessibility within the SEACell framework, we bypassed de novo peak calling on single cells—which yields distinct peak sets for the D30 and Y1 libraries and complicates cross-time-point comparisons—and instead relied on the nonredundant set of 220,884 peaks previously defined from bulk ATAC-seq (D6, D30, and Y1; see section “ATAC-seq preprocessing”). Using ArchR’s addPeakSet function, we intersected this reference catalog with the fragment files for each single cell, produced a peak-by-cell count matrix for both D30 and Y1 and then summed counts across cells belonging to the same SEACell, yielding peak-by-SEACell matrices that are directly comparable across time points and anchored to our bulk data.

For each SEACell, we converted raw fragment counts to reads per kilobase per million (RPKM) to correct simultaneously for sequencing depth and peak length as our bulk peakset include peaks with variable length. Total fragments per SEACell (n_counts) were extracted from the original single-cell object; peak widths were computed from the genomic coordinates encoded in each peak identifier. The RPKM value for peak j in SEACell i was calculated as

$${RPKM}_{ij}=10^{9}\times \frac{{\text{counts}}_{ij}}{{\text{Total}\;\text{counts}}_{ij}\;\times \;{\text{Peak}\;\text{width}}_{ij}}$$

Because the bulk peak catalog had already been classified into four mutually exclusive categories—short-term (ST-memory), long-term (LT-memory), resolved and unchanged—we transferred these labels to the peak-by-SEACell matrices. For every SEACell, we computed the mean RPKM across peaks belonging to each category. To visualize the full distribution of accessibility within each domain, we also generated a *z*-score normalized peak-by-SEACell matrix. Starting from the peak counts aggregated, we centered each peak column by its mean and scaled it by its standard deviation across all SEACells. This standardization, performed independently for every peak, produced a metric that highlights relative gains or losses in accessibility without being influenced by absolute fragment depth. *Z*-scores normalized accessibility values were shown as a heatmap within a value range −1.5 to 1.5. Hierarchical clustering of SEACells was performed on the basis of Euclidean distances and average linkage, while retaining the fixed peak ordering using sns.clustermap function.

### Sample preparation and processing for scRNA-seq

scRNA-seq was performed on FACS-sorted EpdSCs collected at D30 naïve, D30 PIMQ, Y1 naïve, and Y1 PIMQ. Two biological replicates were collected per condition with at least two mice pooled per biological replicate. Each biological replicate was hashed using TotalSeq-B antibodies (table S2) and libraries prepared using 10X Genomics single cell 3′ V3 chemistry. Libraries were sequenced on an Illumina NovaSeq.

### scRNA-seq alignment and background removal

FastQ files were aligned with Cellranger v7.1.0 (85) the transcriptome refdata-gex-mm10-2020A using the command cellranger count indicating the feature references the eight sequences for the different antibody capture (table S2). We used CellBender v.0.3.0 (86) to remove ambient RNA from the raw count matrix (raw_feature_bc_matrix.h5) with a false positive rate of 0.01, 150 epoch, and using the full model. Output CellBender and CellRanger matrices were read using the load_anndata_from_input_and_output function. This anndata was filtered to include cells annotated with a predicted cell probability > 0.5 and preprocessed with Scanpy v1.10. 0 as described below.

### scRNA-seq quality control, normalization, and demultiplexing

For quality control, we performed an iterative process of filtering based on several steps followed by clustering. First, we normalized raw gene expression counts using cell-specific size factors derived from the total transcript count per cell (on the natural scale). Specifically, we first scaled each cell’s expression profile by dividing by its size factor, followed by multiplication by the median size factor across all cells to preserve the overall scale of the data. After normalization, we applied a log-transformation with a pseudocount of 1 (log(x + 1)) to stabilize variance across genes and reduce the influence of highly expressed genes. Our filtering procedure starts with removing cells with < 10 counts and genes expressed in < 10 cells. These permissive thresholds were selected to avoid removing likely viable cells with stringent filters. Given this is a FACS sorted cell type dataset with expected low heterogeneity, we used 5000 top highly variable genes (HVGs) to calculate the PCA with n_comps = 100. Neighborhood graph were calculated using n_neighbors = 31. After clustering with PhenoGraph (87) using ‘leiden’ as clustering algorithm, seed = 0, and k = 30, one small cluster was detected to have high % ribosomal counts and removed. Cells were later removed on the basis of % mitochondrial genes and the number of detected genes (% mitochondrial < 0.30, detected genes < 300).

To identify outlier cells on the basis of their library complexity and transcriptional richness, we applied the LocalOutlierFactor (LOF) algorithm from sklearn.neighbors. This method estimates the local density deviation of a given cell with respect to its neighbors, allowing the detection of cells that lie in regions of lower density—indicative of atypical or poor-quality profiles. We used LOF with 100 nearest neighbors and set the expected contamination rate to 10%, identifying cells with significantly lower density relative to surrounding data points in the space defined by log-transformed library size and number of detected genes resulting in 10,716 cells and 16,499 genes. To perform demultiplexing of hashed single-cell data and assign sample identities to individual cells, we used the HashSolo probabilistic model as implemented in Scanpy. This method estimates the likelihood that each cell corresponds to a negative (unlabeled), singlet (assigned to one barcode), or doublet (tagged with two or more barcodes) on the basis of the distribution of hashtag oligonucleotide (HTO) counts. We provided eight HTO count channels corresponding to experimental samples and specified prior probabilities of 0.01 for negatives, 0.95 for singlets, and 0.04 for doublets, reflecting our expectation that most cells would be valid singlets with a small proportion of doublets and minimal background contamination. From this, 2150 and 41 cells were identified as doublet and negative cells, respectively, and were removed.

The demultiplexed and cleaned AnnData object was normalized and log-transformed again from the raw data. New 5000 HVG were calculated, which were used to obtain the PCA using n_comps = 100. Neighborhood graph were calculated using *n* = 31, which were used to get clusters with *k* = 30, clustering algorithm = ‘leiden,’ and seed = 0

To detect potential doublets in our data that were missed with HashSolo, we applied the BoostClassifier from the DoubletDetection package, which leverages an ensemble-based classification strategy. This method simulates artificial doublets by combining transcriptomes from pairs of real cells and trains a boosted classifier to distinguish between synthetic and real cells. We ran the algorithm for 10 iterations to ensure stability in the doublet probability estimates and found that the model converged at the eighth iteration. The classifier internally performs dimensionality reduction using PCA on the top variable genes and applies PhenoGraph V.1.5.7 clustering to model the structure of the transcriptomic space. Each cell was assigned a doublet score, and final labels were based on the aggregated classification across iterations. Cells classified as doublets were flagged for visualization and excluded from downstream analyses. These filtering steps resulted in 8221 cells and 15,731 genes (D30-Ctrl-1: 1526 cells, D30-Ctrl-2: 1721 cells, D30 PIMQ-1: 1044 cells, D30 PIMQ-2: 1258 cells, 1Yr-Ctrl1: 580 cells, 1Yr-Ctrl-2: 689 cells, 1Yr PIMQ-1: 742 cells, 1Yr PIMQ-2: 661 cells).

### scRNA-seq clustering and cell type annotation

The gene *MALAT1* was excluded before HVG selection owing to its high and ubiquitous expression, which disproportionately influenced the variance structure in our dataset. This exclusion was done to prevent it from dominating the PCA and downstream clustering. We renormalized and log-transformed our data and calculated again the HVGs, PCA, and neighborhood graph. In this case, we selected 58 principal components that represent 70% of the data variance. To identify transcriptionally distinct cell populations, we applied Phenograph clustering using the Leiden algorithm. To determine the optimal number of neighbors (*k*), we systematically evaluated values ranging from 20 to 100 in steps of 5. For each *k*, clustering results were compared pairwise using the Rand index, which quantifies similarity between cluster assignments. We calculated the average Rand index across all pairwise comparisons for each *k* and selected the value with the highest average similarity and lowest variability as the most stable setting (*k* = 55). Under this setting, one cluster showed high expression of *Krt6a* and was found to originate exclusively from a single replicate (D30-Ctrl-2). After manual inspection and verification, we considered this cluster to be potential technical contamination or replicate-specific artifact. While we excluded this cluster from the final dataset to prevent bias, we confirmed that its inclusion did not alter any downstream conclusions or affect the overall interpretation of results.

After excluding the replicate-specific cluster, we repeated the same preprocessing pipeline: highly variable genes were identified, followed by PCA using the top 100 components. The neighborhood graph was constructed using 100 principal components (representing 41% of the variance of the data) and 31 nearest neighbors, and UMAP was recomputed for visualization. Phenograph clustering with the Leiden algorithm was reapplied across a range of *k* values (20 to 100, step 5). To assess clustering stability, pairwise Rand index scores were calculated between all clustering results. The *k* value yielding the highest average similarity and lowest variability across runs was selected as the most robust (*k* = 65) and used for final cluster assignment.

Cell type annotation was performed on the basis of canonical marker gene expression commonly used in skin epidermal biology. We focused on a minimal set of well-established markers to avoid overfitting and to allow clear distinction between major cell states, especially given the limited transcriptional complexity of our scRNA-seq data. UB cells were identified by high expression of *Krt14* and *Krt5*, markers associated with proliferative basal keratinocytes. DB cells were defined by up-regulation of *Krt1* and *Krt10*, which are known to mark cells transitioning toward suprabasal differentiation in addition to the *Krt14* and *Krt5*. Upper Hair Follicle Stem Cells (uHFSCs) were annotated on the basis of strong expression of *Sox9*, a key transcription factor involved in hair follicle stem cell identity.

### scRNA-seq differential expression analysis

Differential expression analysis was performed at the single-cell level using the Wald test from the diffxpy package (v0.7.4+21). For each time point (D30 and Y1), we compared PIMQ and control conditions within each cell type, filtering out genes expressed in <10 cells to reduce noise from low-abundance transcripts. Cells were grouped into two conditions—PIMQ (G1) and control (G2)—and a simple design formula (~1 + condition) was used to model expression differences. Genes were considered significantly differentially expressed if they met all three criteria: adjusted *P* < 0.05, absolute log_2_ fold change > 1, and normalized mean expression > 0.02. These thresholds were chosen to detect biologically meaningful changes while minimizing false positives. The analysis focused primarily on the UB population, the main stem-like compartment relevant to our study.

### IDR peak calling from bulk ATAC-seq

Deep learning models that predict chromatin profiles from DNA sequence require highly reproducible sets of peaks to achieve a good accuracy. To assess the reproducibility of our peak calls across replicates and generate a high-confidence peak set, we performed irreproducible discovery rate (IDR) analysis following the ENCODE pipeline recommendations using two independent replicates per condition. Peaks were independently called on Rep1, Rep2, and the merged TAG file (Rep1+Rep2) using MACS3 as explained previously. The idr tool was run in narrowPeak mode with peaks ranked by *P* value (–rank p.value). Peaks with IDR scores below 0.05 were retained, corresponding to −log10(IDR) > 1.3. Diagnostic plots were also generated to verify the robustness of the analysis. The –use-bset-multisummit-IDR flag was applied to improve resolution for broad or multimodal peaks. To ensure quality and comparability, IDR-passing peaks were filtered against the ENCODE blacklist, and peak scores were capped at 1000 to comply with narrowPeak format. Only peaks on autosomal and sex chromosomes (chr1-chr22, chrX) were retained. The final output consisted of a filtered, high-confidence peak set derived from both replicates and their merged data, suitable for robust training of deep learning models.

### ChromBPNet model for memory establishment

To identify TF motif instances associated with chromatin accessibility during peak inflammation—corresponding to the establishment of epigenetic memory—we used the ChromBPNet architecture (46). The full workflow consisted of four major steps: (i) training a ChromBPNet model to predict chromatin accessibility directly from DNA sequence using our bulk ATAC-seq dataset at day 6 post-IMQ treatment; (ii) using DeepLIFT (88) for estimating per-base contributions to chromatin accessibility predictions; (iii) discovering de novo sequence motifs with MoDISco (89) and annotating them with candidate TFs based on external motif libraries (45, 90, 91); and (iv) detecting high-confidence motif instances across accessible chromatin regions. We selected day 6 post-IMQ because this time point captures peak inflammation, during which chromatin accessibility is thought to establish long-term and short-term epigenetic memory. Modeling TF binding at this stage may reveal regulatory factors responsible for memory imprinting. ChromBPNet operates on a one-hot encoding of 2114 bp DNA sequences centered around ATAC-seq peaks (identified via IDR-filtered MACS3 peak calling, as detailed in the previous section). The model is trained to predict two outputs: the total Tn5 insertion counts over a 1000 bp central region and the base-resolution Tn5 insertion profile across this region. We optimized parameters using a grid search over: number of filters (256, 512, 1024), number of dilated layers (4, 8, 10), and learning rate (0.0001, 0.0010, 0.0050). The best configuration (1024 filter, eight dilated layers, learning rate of 0.0010, other parameters default) was selected on the basis of the Pearson correlation between predicted and observed log counts in the test data, we note that this aligned with the parameters that optimized and the Jensen-Shannon divergence (JSD) of the predicted and observed profiles. The performance of the final model was assessed through fivefold chromosome hold-out cross-validation. Chromosomes were partitioned across folds, with specific chromosomes reserved for testing and validation in each iteration: fold 0 (test: chr1, chr3, chr6, validation: chr8, chr19), fold 1 (test: chr2, chr8, chr17; validation: chr5, chr10, chr18), fold 2 (test: chr5, chr9, chr11; validation: chr12, chr14), fold 3 (test: chr4, chr10, chr18; validation: chr2, chr7), and fold 4 (test: chr5, chr15, chr12; validation: chr3, chr6). The predictive accuracy for the count output was evaluated using Spearman’s correlation for log-transformed counts, while the profile output was assessed using JSD.

To interpret model predictions, we applied the DeepLIFT algorithm (88), which quantifies the contribution of each base to the predicted chromatin accessibility. Contribution scores were calculated as the additive decomposition of the difference between the predicted log(total counts) of the native sequence and the average prediction across 20 dinucleotide-shuffled background sequences. Because we had five different models (one model for each fold), we calculated the contribution scores for each model, and these were averaged to have a final global contribution score that was used for de novo TF motif discovery. Motif discovery was conducted using TF-MoDISco (89) (v0.5.16.4.1; https://github.com/kundajelab/tfmodisco), which clusters high-contribution subsequences (seqlets) to identify representative, nonredundant motifs across accessible chromatin regions. Motif discovery was performed over 500-bp windows centered on peak summits, with a maximum of 50,000 seqlets considered per metacluster. Identified motifs were subsequently annotated to putative TFs or TF families through comparison with motifs from the MEME v4 database using the TOMTOM (92) tool, using the command tomtom -no-ssc -oc . -verbosity 1 -text -min-overlap 5 -mi 1 -dist pearson -evalue-thresh 10.0.

Finally, to identify these specific predictive instances of the de novo motifs, we scanned contribution score tracks using FiNeMo (v0.25; https://github.com/austintwang/finemo_gpu). Briefly, FiNeMo uses sparse linear regression to reconstruct regional contribution score profiles from a weighted combination of candidate motifs. Specifically, for each peak region, FiNeMo seeks to identify motif instances by fitting trimmed candidate contribution weight matrices (CWMs) to observed scores, thereby minimizing the discrepancy between the observed and reconstructed profiles. Positions assigned nonzero coefficients in this process represent predictive motif instances, corresponding to short genomic segments where both motif sequence matches and elevated absolute contribution scores coincide. We applied FiNeMo using the complete set of the de novo CWMs discovered by TF-MoDISco, setting parameters to –alpha 0.7 and –trim-threshold 0.3, with default values for remaining parameters. These high-confidence motif instances were later intersected with memory domains to evaluate their role in establishing long-term accessibility.

### Functional validation of ChromBPNet-predicted AP1 motifs

To assess the biological relevance of the de-novo AP1 motif (Motif #0) identified by our ChromBPNet model, we analyzed two independent datasets: (i) ATAC-seq generated after expression of a dominant-negative FOS construct (AFOS) and (ii) CNR-seq profiles for c-FOS, cJUN, STAT3, and TP63 obtained under identical D6-IMQ conditions. Motif #0 occurrences were first located within the set of memory domains with FiNeMo (see ChromBPNet model for memory establishment section). For each occurrence, the instance-level contribution score was defined as the sum of absolute per-base contributions output by ChromBPNet. Instances were ranked by this score and binned into deciles. Memory domains containing at least one instance in the top decile (90 to 100th percentile) and no lower-ranked instances were designated high-contribution domains; those harboring exclusively 10 to 20th percentile instances were designated low-contribution domains. Domains with mixed-rank instances were omitted from further analysis. AFOS ATAC-seq libraries generated 24 hours after D6-IMQ stimulation (two biological replicates; GEO GSE171596) were processed with the alignment workflow described in the section “ATAC-seq preprocessing”. Fragment counts were summarized per domain, normalized to CPM and visualized with the *plotProfile* utility in deepTools. The same processing pipeline (“CNR-seq preprocessing” section) was applied to our previously published CNR-seq datasets for c-FOS, c-JUN, STAT3, and TP63 (GEO GSE171596). For each TF, CPM values were extracted for every domain, and *plotProfile* was used to compare signal distributions between high- and low-contribution groups. This approach enabled direct functional validation of ChromBPNet-predicted AP1 motif importance through both chromatin-accessibility perturbation (AFOS) and transcription-factor binding measurements.

### PersistNet: Persistence score as per-base metric to explore memory maintenance

To study the persistence of chromatin accessibility over time, we sought to develop a model to directly train on what interests us, the persistence of memory domains. The individual chromatin tracks at each time point do not capture the notion of persistence, as each individual peak is different and a peak at Y1 does not provide relative information to peak height at D30. Therefore, instead of training separate ChromBPNet models for each time point, which would reduce comparability across datasets, we developed an approach that attempts to directly predict persistence. The notion of persistence we wish to quantify is how much of the peak was retained at Y1, relative to its abundance at D30. Therefore, we consolidated our postinflammation time points—D30 and Y1—and developed the per-base persistence score metric. This score quantifies, at single-nucleotide resolution, the relative stability of accessibility across these time points. A high persistence score reflects sustained accessibility between D30 and Y1, while a low score indicates regions that lost accessibility over time. We selected D30 and Y1 to be consistent with the definition of short-term domains (significantly accessible at D30 but not significant at Y1), and long-term domains (significantly accessible at D30 and Y1). Moreover, these time points had higher coverage, as well as more biological replicates (four biological replicates for each time point).

First, to achieve the read depth required for single-nucleotide scoring, we merged the paired-end fragment files from the four biological replicates available at each time point, obtaining 215 million unique fragments for D30 and 175 million for Y1 datasets. Then, we normalized each dataset by sequencing depth using CPM at single base-pair resolution and multiplied by 1000 to generate counts per billion (CPB). We applied a minimal coverage filter to the D30 signal to exclude low-confidence positions. Specifically, we retained only positions where the D30 ATAC-seq signal exceeded a value of 5 CPB, which corresponds to one raw TN5 insertion in the merged dataset. At every position passing this filter, we computed a persistence score as follows

$${\text{Persistence}\;\text{score}}_{i}=10\times \frac{4}{\pi }\times arctan\left(k\times \frac{{CPB}_{Y1,i}}{{CPB}_{D30,i}}\right)$$

where ${CBP}_{Y1,i}$ and ${CBP}_{D30,i}$ represent the normalized chromatin accessibility values (in CPB) at nucleotide “i” for Y1 and D30, respectively. The arctangent transformation compresses extreme Y1/D30 ratios, preventing outliers from dominating the dynamic range, while the factor $10\times \frac{4}{\pi }$ rescales the output so that scores span from 0 to 20 closely mirroring the count magnitudes typically observed in ATAC-seq and facilitating more comparable ranges for BPNet input. The dimensionless scaling constant k controls the steepness of the arctangent curve: Values k>1 emphasize large fold changes, whereas k<1 accentuate modest differences. To select k, we performed a five-point grid search {0.1, 0.5, 1, 2, 5} by evaluating how well the resulting persistence scores discriminated the 117 long-term from 117 short-term memory domains, using the area under the ROC curve (AURC) alongside sensitivity and specificity. The optimal value, k=0.5, maximized AUC while maintaining balanced sensitivity and specificity.

### PersistNet: BPNet-based model to predict persistence score from DNA sequence

To estimate the persistence of chromatin accessibility directly from primary DNA sequence we implemented a two-head convolutional neural-network inspired by BPNet deep learning model (47). The architecture (fig. S5A) preserves the key design principles of BPNet—causal dilated convolutions and residual connections—while (i) restricting the model to a single biological task and (ii) producing two complementary outputs for every 2114 bp input sequence:

- Profile head—a vector of length L (here 1000) that represents, at single-nucleotide resolution, the predicted distribution of persistence scores along the centered sequence.
- Count head—a single scalar that summarizes the average persistence within the same window and serves as a regularizer for the profile prediction.

We opted for the compact BPNet architecture over ChromBPNet as our persistence score is a function of the ratio of two ATAC-seq tracks over the same single nucleotide position which mitigates the known sequence biases of ATAC-seq. Indeed, de novo motif discovery did not recover any Tn5-related motifs across multiple models, suggesting minimal to no confounding from transposase sequence preferences. Lastly, evaluation metrics (Pearson correlation and JSD) showed similar performance between our model and classical ChromBPNet implementations.

### PersistNet: Model architecture

Input is a one-hot–encoded DNA segment (4 × 2114). A first Conv1D layer (21-bp kernel, *ReLU*) extracts local k-mer features. Eight residual blocks follow, each consisting of a 3-bp dilated convolution (dilation 2^*i*^, *i* = 1…8), symmetric cropping to maintain center alignment, and elementwise addition to the incoming tensor. All convolutions use the same number of filters (*f* = 512, selected by grid search). The profile head applies a wide (75-bp) convolution without activation, then crops the output so that its receptive field is centered on the input window. We finally flatten the tensor to obtain logits of length L. The count head first collapses the spatial dimension with global-average pooling and feeds the resulting vector into a fully connected layer (units = 1). Training minimizes a joint loss

$$L=L_{\text{profile}}+\lambda L_{\text{counts}}$$

where $L_{\text{profile}}$ is the multinomial negative log-likelihood between observed and predicted per-base persistence distributions, $L_{\text{counts}}$ is the mean-squared error on the scalar output, and λ (set to 1) balances the two terms. The Adam optimizer (learning rate 0.001) is used with deterministic behavior enforced by fixing Python, Numpy, TensorFlow and random-seed states.

Per-base persistence tracks were computed from the D30 and Y1 post-IMQ ATAC-seq datasets. Candidate regions were selected as peaks identified by MACS3 followed by IDR filtering (see section “IDR peak calling from bulk ATAC-seq”). To construct training examples, we performed two sequential filters on these peaks:

1. Edge filter. Regions whose centers lay <500 bp from chromosome ends or bigWig boundaries were removed ensuring that every extracted window is fully represented in both input and output tensors.
2. Outlier filter. For robustness, we discarded the top and bottom 1% of peaks on the basis of total counts (upper = 99th percentile, lower = 1st percentile), producing the final training set of 77,753 peaks.

The median of the counts from the final peaks set is divided by 10, providing an empirical scale for the count-loss weight, which stabilizes learning by matching the magnitude of both loss components.

For every mini-batch, training examples were generated in real time by extracting centered windows with a random positional jitter of ±250 bp. The jitter was applied consistently to both input and target persistence profiles, serving as data augmentation to prevent positional overfitting. To optimize the model, we performed a grid search across several hyperparameters: number of filters (256, 512, 1024), number of dilated layers (4, 6, 8), learning rate (0.001, 0.005), and jitter (50, 100, 250, 500), which increases dataset variability by introducing positional shifts in training examples. The optimal configuration used 512 filters, eight dilated layers, a learning rate of 0.001, and jitter of 250. Five-cross validation were performed with specific chromosomes reserved for testing and validation in each iteration: fold 0 (test: chr1, chr3, chr6, validation: chr8, chr19), fold 1 (test: chr2, chr8, chr17; validation: chr5, chr10, chr18), fold 2 (test: chr5, chr9, chr11; validation: chr12, chr14), fold 3 (test: chr4, chr10, chr18; validation: chr2, chr7), and fold 4 (test: chr5, chr15, chr12; validation: chr3, chr6).

We trained our model using the bigWig track of per-base persistence scores (see section “PersistNet: Persistence score as per-base metric to explore memory maintenance”) and selected the peaks which passed IDR threshold obtained from D30 PIMQ dataset (see section “IDR peak calling from bulk ATAC-seq”). We used the D30-PIMQ dataset because it captures both short- and long-term memory domains, and because inflammation-related chromatin changes are largely resolved, making it a stable reference time point.

After training, model interpretation was carried out with the chromBPNet interpret command, which implements the DeepLIFT algorithm (88). Briefly, for every 2114-bp input sequence, we obtained a vector of contribution scores that quantify the impact of each nucleotide on the scalar persistence count head predicted by the model. Because five independent models were produced during cross-validation, we averaged the contribution maps across folds and used this track to generate the final bigWig file; this fold-averaging markedly reduces variance introduced by random weight initialization and ensures that downstream motif discovery is driven by stable, biology-dependent signal.

De novo motif discovery followed the TF-MoDISco workflow (version 0.5.13). Briefly, TF-MoDISco identified and extracted the 50,000 highest-scoring seqlets (short contiguous segments with high cumulative contribution score) drawn from 500-bp regions centered on each peak summit. Seqlets were clustered with default cosine distance, and clusters were refined into CWMs that describe the average importance of each base at every position. Identified motifs were subsequently annotated to putative TFs or TF families through comparison with motifs from the MEME v4 database using the TOMTOM tool, using the command tomtom -no-ssc -oc. –verbosity 1 -text -min-overlap 5 -mi 1 -dist pearson -evaluethresh 10.0.

We next searched for motif instances inside the 117 short-term and 117 long-term memory domains with FiNeMo (commit 1d5b88c). Because FiNeMo was originally designed for rapid, genome-wide motif scanning rather than high-resolution analysis of small regions, we adjusted its parameters to better suit our focused dataset of 234 loci. Specifically, we relaxed the regularization threshold (–lambda = 0.25; default = 0.7) to increase sensitivity and recover a broader set of candidate motif instances within these limited regions. This preliminary expansion allowed us to apply a secondary stringent filter based on the same cosine-similarity metric used by TF-MoDISco, which incorporates base-pair–level contribution information and aligns with our motif-discovery workflow. Motif instances were retained only if they satisfied both cosine similarity ≥ 0.75 and coefficient score ≥ 2, criteria that effectively enrich for true positive hits while maintaining consistency with MoDISco’s definition of high-confidence sequence matches.

### De novo motif enrichment analysis within short- and long-term memory domains

To ensure high-confidence hit instances and avoid redundancy, we applied two sequential filtering steps to the identified motif hits. First, for motifs detected on both positive and negative strands at the same genomic coordinates, we retained only the instance with the highest correlation score to the de novo discovered motif pattern, as this represents the best match to the original consensus sequence. Second, we addressed potential redundancy arising from overlapping motif instances within each de novo motif. For each TF motif, hits were ranked by their correlation scores and examined pairwise for overlaps. When two motif instances overlapped by more than 3 nucleotides on the basis of their genomic coordinates, we retained only the instance with the higher correlation score and discarded the lower-scoring overlapping hit. This hierarchical filtering approach prioritized the most statistically robust motif matches while eliminating redundant or lower-confidence instances that could confound downstream analyses.

De novo motifs differentially enriched between long- and short-term memory domains were identified using two complementary scoring approaches. The first approach quantified motif enrichment on the basis of instance frequency, counting the number of motif occurrences within each memory domain. The second approach incorporated a weighted importance score, calculated as the sum of contribution scores (from the FiNeMo output) for all motif instances within each domain, thus accounting for both the quantity and quality of motif matches. For both scoring methods, only de novo motifs present in at least 20 memory domains were retained for statistical analysis. Differential enrichment between long- and short-term domains was assessed using unpaired *t* tests, with *P* values adjusted for multiple testing using the Benjamini-Hochberg procedure. Motifs with adjusted *P* values < 0.05 were considered significantly enriched. Additionally, we calculated the prevalence of each motif across domains by determining the number of memory domains containing at least one instance, providing insight into which de novo motifs were broadly representative of long-term memory domains versus those with more restricted distributions.

The correspondence between identified hits from memory domains and annotated TF motifs was validated through PWM reconstruction and comparison. PWMs were generated from all detected hits across the 234 memory domains (117 short-term and 117 long-term domains). These reconstructed PWMs were then compared against the original annotated TF motifs using TOMTOM with the following parameters: -no-ssc -minoverlap 5 -mi 1 -dist pearson. Statistical validation was achieved through a q-value threshold of < 0.05, confirming that the hits identified in memory domains accurately represented the expected binding patterns of their corresponding annotated transcription factors.

### Memory domain C or G frequency and CG density per kilobase

We extracted the nucleotide sequences for each domain using BSgenome. Mmusculus.UCSC.mm10 as the reference mouse genome. Then we calculated the “C or G density” as the number of C or G within the sequence normalized per kilobase. Analogously, we calculated the “CpG density” as the number of CpG dinucleotides per kilobase of sequence. Specifically, we counted how many times the dinucleotide “CG” appears in each sequence. For both cases, the count was then normalized by dividing it by the total number of bases in the sequence, and the result was multiplied by 1000 to convert it into a measure of CpG occurrences per kilobase.

### In silico mutation analyses using PersistNet model

To evaluate the functional role of CG dinucleotides in sustaining chromatin accessibility, we performed three in silico mutation strategies using our trained PersistNet model. These analyses aimed to determine whether CpG is uniquely predictive of long-term accessibility, whether its dosage correlates with persistence, and how its disruption affects native memory regions.

1. **Comparing CG with other dinucleotides.** First, we tested whether CG dinucleotides uniquely promote persistence relative to the other 15 possible dinucleotide combinations. For each of the 16 dinucleotides, we generated a specific background set of 500 synthetic sequences, each 1000 bp in length, that completely lacked the target dinucleotide (zero occurrences) while maintaining a realistic GC content (~45%), matching the typical composition of our memory domains. The sequence generation process involved creating random sequences with the specified GC percentage content, then systematically eliminating any occurrences of the target dinucleotide by substituting individual nucleotides with alternatives that would not recreate the forbidden dinucleotide. This ensured that each dinucleotide was tested under identical conditions: comparing complete absence (zero occurrences) versus controlled presence (20 occurrences). For the test condition, we inserted exactly 20 occurrences of each dinucleotide at predetermined positions with a minimum separation of 30 bp to avoid clustering effects. The value of 20 dinucleotides per kilobase was chosen to resemble the average CpG density observed in our long-term memory domains. To prepare sequences for prediction with the PersistNet model (which requires 2114 bp input), we padded each 1000 bp sequence with 557 bp flanking regions on each side. Critically, these flanking sequences were also generated to exclude the target dinucleotide, preventing any unintended introduction of the tested dinucleotide outside the central region. The resulting sequences were stored in FASTA format: 500 background sequences (zero occurrences) and 500 test sequences (20 occurrences) for each dinucleotide, totaling 16,000 sequences. Predictions were performed using the count head of the persistence model, which outputs a scalar value representing predicted chromatin persistence for the central 1000 bp region. We chose this output head because benchmarking showed that the model more reliably captured signal intensity through the count head than the profile head. To assess the effect of each dinucleotide, we calculated paired differences (test minus background) for each of the 500 sequence pairs per dinucleotide. This paired design controls for sequence-specific variation and provides a direct measure of each dinucleotide's contribution to chromatin persistence. Statistical significance was assessed using the Wilcoxon signed-rank test for paired samples, with Bonferroni correction for multiple testing across all 16 dinucleotides.
2. **Assessing the effect of CpG dosage.** Next, we investigated whether the number of CpG sites correlates positively with predicted persistence. Starting again from the same 500 background sequences described above, we sequentially replaced random dinucleotide positions with 1 to 50 CGs, generating 50 groups of 500 sequences each. Sequences were extended to 2114 bp by adding 557 bp of AT-only sequence on each flank to ensure that flanking regions did not introduce unintended CGs or affect GC content. We then used our persistence model to predict persistence scores for each sequence and calculated the Pearson correlation between the number of CpGs and the model’s predicted persistence. This approach allowed us to determine whether there is a dose-response relationship between CpG content and chromatin accessibility persistence.
3. **Disrupting CGs in a real long-term memory region.** Finally, to test the functional necessity of CG sites within a native long-term memory domain, we selected a well-characterized region associated with *Aim2*, chr1:173,420,346–173,420,754, which spans 409 bp and contains 6 CpG sites. To meet the model’s required input length, we expanded this sequence symmetrically to chr1:173,419,494–173,421,608, covering 2114 bp of native genomic context. We then performed systematic CG disruption. In the first set, we generated 100 mutated versions of the region where one of the six CG sites was randomly selected and replaced with one of the other 15 dinucleotides (e.g., CA, TG, AA, etc.). This yielded 90 unique combinations (6 positions × 15 replacements), and the remaining 10 were generated by sampling with replacement. We repeated this procedure to generate groups where two, three, four, five, or all six CGs were replaced, creating a total of six mutation groups of 100 sequences each. This allowed us to explore how incremental loss of CG sites affects predicted persistence in an authentic genomic context. All predictions were performed using the ensemble of five persistence models (one per fold), and the resulting scores were averaged across models to increase robustness.

### Analysis of memory domain CpG methylation dynamics

We quantified DNA methylation trajectories at the regulatory-domain scale to compare LT and ST memory classes. For each time point (D6, D30, D180, Y1, Y1.5, and Y2), all CpG sites whose genomic coordinates fell within annotated LT or ST domains were enumerated and aggregated into a per-domain mean methylation fraction. This domain-level summarization captures global dynamics within the regulatory unit used by our model while reducing sensitivity to outlier CpGs and heterogeneity in CpG density across regions. These per-domain means were then contrasted between LT and ST classes and across time relative to age-matched naïve controls to establish class-specific methylation baselines and trajectories.

To test whether ETS1 engages memory domains during acute inflammation—when these regions undergo robust demethylation—we processed ETS1 CNR data from D6 IMQ-treated and naïve skin and depth-normalized signals as RPKM. For each memory domain, we extracted ETS1 signal in ±2 kb windows centered at the domain midpoint, binned the tracks, and averaged within class to yield metaprofiles. We generated heatmaps with one row per domain, sorted within class by D6 IMQ signal, enabling simultaneous visualization of the class mean and domain-to-domain heterogeneity.

We next assessed whether the sequence features recovered by the persistence model correspond to experimentally bound ETS1 sites within memory domains. PersistNet-predicted ELK/ETS instances (motif #5) were identified within all memory domains (*n* = 934 domains), aligned on their centers (±5 bp), and ranked by their mean base-level contribution to persistence. To integrate sequence grammar, model attributions, and binding, motif instances were grouped by their 6-mer core and, for each group, we computed (i) frequency across memory domains, (ii) a min–max–scaled mean persistence contribution (across all 6-mer groups), and (iii) *z*-scored ETS1 CNR signal (CPM) in D6 IMQ and in naïve skin. This joint analysis tests whether grammars with high predicted contributions correspond to ETS1 binding during inflammation, thereby validating that model-implicated instances represent functional sites rather than spurious matches. Having established the utility of model-implicated instances, we refined methylation analyses from the domain level to individual CpGs. We first considered all CpG sites contained within memory domains and tracked their methylation longitudinally across the six time points, treating each CpG as one observation within its class; this provided a high-resolution view of per-site dynamics independent of local CpG density or domain size (short-term: 1188 CpGs; long-term: 2071 CpGs). We then imposed a sequence-level filter by restricting to CpGs that reside inside ELK/ETS motif #5 instances within memory domains; these CpGs were stratified by class to compare trajectories for motif-embedded sites (short-term: 209 CpGs; long-term: 389 CpGs). Finally, to couple occupancy with methylation at model-implicated sequences, we applied a stringent binding filter by retaining only motif #5 instances that showed evidence of ETS1 binding at D6 IMQ, defined as ETS1 CNR > 0.5 CPM over the exact motif coordinates. CpG methylation within bound motif center at every time point was tracked from D6 through Y2. This yielded a high-confidence set of ETS1-engaged CpGs for direct class comparisons (long-term: 128 CpGs; short-term: 76 CpGs), minimizing confounding from unbound or weakly bound instances.

To quantify the relationship between DNA methylation and the model’s predicted importance for memory longevity, we paired each CpG’s methylation fraction with the PersistNet-assigned contribution at that exact CpG position (log_2_-transformed) and examined this association at D30 post-IMQ, when LT and ST trajectories begin to diverge. Correlations were computed separately within LT and ST classes (Pearson correlation), providing an orthogonal test of whether higher predicted persistence contribution coincides with sustained hypomethylation.

Lastly, to evaluate whether the persistence-predictive grammar corresponds to durable hypomethylation over time, the ranked all ELK/ETS motif #5 instances inside memory domains by mean persistence contribution were used to select two nonoverlapping cohorts: the top 1000 (high-contribution) and the bottom 1000 (low-contribution) instances. For each instance, CpGs at the motif center were identified and followed across the six time points. Heatmaps were used to visualize temporal patterns; within each cohort, rows were ordered by mean methylation at the D6 IMQ condition. Instances lacking a central CpG were excluded from heatmap visualizations.

### BPNet model to identify sequence determinants of H2A.Z deposition

We used the same BPNet architecture as previously described to train a model that learns H2AZ signal track at D30 PIMQ from DNA sequence. The model was trained to predict H2A.Z deposition across memory domains using both count and profile losses using the same peakset used for our persistence model (77,753 peaks). While both losses were included—count loss (mean squared error) for predicting H2A.Z levels and profile loss JSD) for the shape of the per-base persistence distribution—the primary focus during training was on the count loss. This was due to the broader nature of H2A.Z deposition, which, in contrast to transcription factors or other narrower genomic signals, does not have the same localized peaks but instead exhibits a broader chromatin modification pattern. Thus, despite using both losses, the model was trained with an emphasis on learning the count head (count_loss_weight = 50,000), ensuring that the model captured the overall distribution of H2A.Z deposition more effectively. Data augmentation was performed by introducing random positional jitter (±250 bp) to enhance the model’s generalization. In a similar fashion, for model interpretation, we used DeepLIFT to obtain contribution scores for each nucleotide regarding counts head, which were averaged across folds. This provided insight into the nucleotide-level contributions to H2A.Z deposition, helping us identify key motifs involved in this broader chromatin mark.

### H2A.Z deposition and total loss of DNA methylation as predictors of memory domain longevity

We aimed to use metrics obtained from either sequence features or sequencing experiments at the early resolution time point (D30) to distinguish long-term from short-term memory domains accurately. To achieve this, a logistic regression model was constructed using RPKM normalized H2A.Z signal at D30 PIMQ and the total loss of DNA methylation as predictors; and the LT/ST category as binary dependent variable. The total loss of DNA methylation was defined as a metric that combines two key factors: CpG density and ΔmCG (delta methylation between the naïve and PIMQ conditions at D30). CpG density is calculated as the number of CpGs per kilobase within a memory domain, reflecting the capacity of a region to undergo cytosine methylation. ΔmCG captures the average difference in methylation levels between D30 PIMQ and control conditions, measuring the change in methylation state between these conditions. The total loss of DNA methylation places greater emphasis on regions where average methylation changes are both significant and occur in CpG-rich areas, which are known to be more sensitive to methylation-driven chromatin modifications.

This model enabled the determination of a decision boundary: domains with higher CpG impact scores and greater H2A.Z deposition were more likely to be classified as long-term memory domains. For model validation, a 10-fold cross-validation strategy was used. Model performance was evaluated using metrics such as the ROC curve and accuracy, ensuring the robustness and reliability of the model in correctly classifying long-term and short-term memory domains. This method, while simple in its formulation, offers valuable insights into the predictive role of DNA methylation and chromatin remodeling for the persistence of epigenetic memory.

### MINT-ChIP

MINT-ChIP was performed as described (30), using 50,000 FACS-purified EpdSCs per target histone mark for each biological replicate, with two biological replicates per condition and two or three mice pooled per biological replicate. Briefly, purified samples were split in two (25,000 cells each), then lysed for 20 min on ice in the presence of either low (60 units) or high (120 units) concentration of micrococcal nuclease (NEB). Sample/MNase concentration-specific T7 adapters were then ligated to each sample. After ligation, samples for each MNase concentration were pooled together, then split evenly for overnight incubation with ChIP antibodies in 4°C (table S1). Antibody-bound DNA was isolated the next day through 4 hour incubation with protein G beads in 4°C, after which samples were washed twice with ice-cold radioimmunoprecipitation assay (RIPA) buffer, then once each with high salt RIPA buffer, LiCL wash buffer, and TE buffer. Eluted DNA was then cleaned and subjected to overnight in vitro transcription in 4°C. Resulting RNA was isolated and reverse transcribed, then final library PCR was performed with sample-specific barcodes for pooled sequencing. Finally, libraries were cleaned, pooled, then sequenced for 50 bp paired-end reads on an Illumina NextSeq P2000.

### Analysis of MINT-ChIP

MINT-ChIP reads were processed as described (30). Reads were first demultiplexed into FASTQ files specific for each target histone mark and MNase digest condition using Illumina’s bcl2fastq2 and further demultiplexed into sample-specific FASTQ files using the T7 adapter barcode from read2. Demultiplexed reads were trimmed for adapters using Skewer and aligned to the mm10 genome (GENCODE v.30) using Bowtie2. Duplicated reads were marked and removed using Picard, then replicates per condition and MNase digest conditions (high/low) merged using Samtools. For visualization, bigWig files were generated using the bamCoverage function in deeptools (version 3.5.5) with RPKM normalization, and summary counts of bigWigs over ATAC-seq peaks generated using deeptools computeMatrix. All plots were created using the profileplyr R package (version 1.4.3).

### Cut-and-Run sequencing

CNR was performed as previously described (22), using 500,000 FACS-purified EpdSCs per biological replicate per target antibody, with two biological replicates per condition and two or three mice pooled per biological replicate. Cells were lightly fixed in cross-linking buffer (10 mM HEPES NaOH pH 7.5, 100 mM NaCl, 1 mM EGTA, 1 mM EDTA, 1% formaldehyde) and rotated at room temperature for 10 min. Cross-linking was quenched with glycine (0.125 M final concentration) and rotated at room temperature for 5 min. Cells were washed with cold PBS and nuclei isolated by resuspending in NE1 buffer (20 mM HEPES-KOH pH 7.9, 10 mM KCl, 1 mM MgCl_2_, 1 mM DTT, 0.1% Triton X-100, with Roche complete EDTA-free protease inhibitor) and rotated at 4°C for 10 min. Nuclei were washed twice with wash buffer (20 mM HEPES pH 7.5, 150 mM NaCl, 0.5% BSA, 0.5 mM spermidine, with protease inhibitor) then incubated with concanavalin-A (ConA) beads previously washed with binding buffer (20 mM HEPES-KOH pH 7.9, 10 mM KCl, 1 mM CaCl_2_, 1 mM MnCl_2_), for 10 min at 4°C. ConA-bead-bound nuclei were incubated overnight at 4°C in antibody buffer (Wash buffer supplemented with 0.1% Triton X-100 and 2 nM EDTA) and antibody (table S1). ConA-bound nuclei were washed once with triton wash buffer (wash buffer supplemented with 0.1% Triton X-100) then resuspended in antibody wash buffer with 2.5 μl pAG-MNase (EpiCypher) and incubated at 4°C for 1 hour. ConA-bound nuclei were washed twice with triton wash buffer and resuspended in 100 μl of lysis buffer (50 mM Tris-HCl, 150 mM NaCl, 1% Triton X-100, 0.1% sodium deoxycholate, 5 mM CaCl_2_) and incubated iced metal block (0°C) for 30 min. MNase reaction was stopped with addition of 100 μl 2x stop buffer (30 mM EGTA) and incubated at 37°C for 10 min. After incubation, ConA-bound nuclei were captured using a magnetic tube holder and supernatant containing CNR DNA fragments was collected. Supernatant was incubated with 2 μl 10% SDS and 2.5 μl 20 mg/ml proteinase K at 70°C for 2 hours. DNA was purified using PCI and overnight ethanol precipitation with glycogen at −20°C. DNA was spun down and washed once with 100% ethanol before being resuspended in 15 μl of buffer EB. Sequencing libraries were generated using NEBNext Ultra II DNA library Prep Kit for Illumina (Index Primer set 2). PCR-amplified libraries were purified using 1.2x ratio of AMPure XP beads and eluted in 15 μl 0.1x TE buffer. Libraries were pooled and sequenced on Illumina NextSeq using 40-bp paired-end reads.

### Analysis of Cut-and-Run sequencing

Forty base pair paired-end reads raw reads were aligned to the mouse reference genome (mm10, GENCODE v.30 annotation) using BWA-MEM in paired-end mode with the -M and -T 10 parameters to mark split alignments and increase mapping stringency. Alignment quality was confirmed for all samples, with mapping rates exceeding 80%. Resulting BAM files were processed using a standardized filtering pipeline. Reads were filtered with SAMtools (-F 1804 -f 2 -q 30) to retain only properly paired reads with a mapping quality score ≥30, while excluding unmapped reads, secondary alignments, optical duplicates, and reads failing vendor or platform quality control. PCR duplicates were marked using Picard and subsequently removed with SAMtools. To focus the analysis on canonical autosomes and the X chromosome, only alignments mapping to chromosomes 1 to 19 and X were retained; reads aligning to nonstandard scaffolds or blacklisted regions were removed using BEDTools intersect with ENCODE blacklist annotations. Signal tracks were generated by computing genome-wide coverage using the coverage function from the rtracklayer package. Coverage values were normalized to CPM to account for differences in sequencing depth across samples. The resulting bigWig files were used as input for DeepTools. Specifically, computeMatrix was run in reference-point mode, centered on regions of interest with a 1000 bp window upstream and downstream. Aggregated signal profiles were visualized using the plotProfile function, enabling direct comparison across conditions and genomic features.

### RNA-seq

Two hundred fifty thousand EpdSCs per condition were FACS-sorted directly into room temperature buffer RLT (QIAGEN) and immediately flash frozen. Two biological replicates were used per condition, with two or three mice pooled per biological replicate. Total RNA was isolated with RNeasy Mini Kit (QIAGEN) as per manufacturer’s instructions. Libraries were then prepared using Illumina TruSeq standard mRNA library kit and sequenced on Illumina NextSeq using 40-bp paired-end reads. Reads were aligned to the mouse exons using the mm10 reference genome (GENCODE v.30), exon positions from TxDb. Mmusculus.UCSC.mm10.knownGene and Rsubread for alignment, Aligned reads were counted using the summarizeOverlaps() function of the GenomicAlignments R package (v1.38.2). Raw counts were used as input into DESeq2 (v1.42.1) (81) for differential gene expression testing with default parameters. To investigate whether genes associated with memory domains showed coordinated transcriptional changes upon rechallenge, we performed GSEA (93) using bulk RNA-seq data from Y1 PIMQ + TPA versus Y1 naïve + TPA conditions. Custom gene sets were derived from the proximity-based annotation of memory domains, including long-term memory genes, short-term memory genes, all memory genes, resolved domain genes, and unchanged domain genes. RNA-seq differential expression results were filtered to include genes with baseMean > 50 to ensure sufficient expression levels for reliable analysis. Genes were ranked by log_2_ fold change, and GSEA was performed using the clusterProfiler R package (94) (v4.10) with custom TERM2GENE mappings. Statistical significance was assessed using 10,000 permutations, with a minimum gene set size of 10 genes and a maximum of 1000 genes. *P* values were adjusted for multiple testing using the Benjamini-Hochberg method.

### Permutation test of memory domain overlap with wound-accelerated genes

Memory (*n* = 934 peaks), resolved (*n* = 934), and all unchanged domains (*n* = 16,987) were annotated to their closest gene as described in the section“ Memory domain–associated genes and gene scores from ATAC-seq.” Wound-accelerated genes were assessed via reanalysis published RNA-seq of 12 hour-wounded EpdSCs from either D30 naïve or D30 PIMQ skins (4). Raw RNA-seq reads were obtained from GSE92697, and DESeq2 was used as above to detect genes accelerated in D30 PIMQ EpdSCs upon secondary wounding (log_2_ fold change > 1, *P-adj* < 0.05; *n* = 62 genes). Five thousand random permutations of 934 unchanged domains were used to derive a background distribution of random genomic domain association with PIMQ-accelerated genes. Permutation tests were then performed using the permTest() function in the regioneR package (v1.30.0) to determine the significance over chance of the observed overlap between wound-accelerated genes and proximity-associated memory or resolved chromatin domains.

### Whole-genome bisulfite sequencing

Two hundred thousand EpdSCs were FACS purified, washed once with PBS, and snap frozen before undergoing DNA extraction. Each biological replicate from each condition consisted of at least two pooled mice. The DNA was sheared in Covaris micro TUBE AFA Fiber Pre-Slit Snap-Cap tubes (SKU: 520045) and cleaned up with the Zymo DNA Clean & Concentrator-5 kit (#D4013) following manufacturer’s guidelines. Sheared gDNA was bisulfite-converted following manufacturer’s guidelines with the EZ DNA Methylation-Gold Kit (Zymo #D5005), and libraries were prepared using the Accel-NGS Methyl-seq DNA library kit (Swift Biosciences, #30024-SWI). Libraries were cleaned using Agencourt AMPure XP beads (Beckman Coulter, #A63881), and the absence of adapters was confirmed on the Agilent TapeStation HS D5000. The final libraries were sequenced on a NovaSeq platform (Illumina) yielding 150 bp paired-end reads.

Raw reads were subjected to adapter and quality trimming using cutadapt (v4.6; parameters: -quality-cutoff 20 –overlap 5 –minimum-length 25; Illumina TruSeq adapter clipped from both reads), followed by trimming of 10 and 5 nucleotides from the 5′ and 3′ end of the first read and 15 and 5 nucleotides from the 5′ and 3′ end of the second read. Trimmed reads were aligned to the mouse genome (mm10) using BSMAP (v2.90; parameters: -v 0.1 -s 16 -q 20 -w 100 -S 1 -u -R). Sorted BAM files were generated and indexed using samtools with the “sort” and “index” commands (v1.10). Duplicates were removed using GATK4 (95) (v4.5.0.0) MarkDuplicates with lenient validation, duplicate removal enabled, and coordinate-sorted input. Methylation rates were called using mcall from the MOABS package (v1.3.9.6; default parameters). All methylation analyses were restricted to autosomes, and only CpGs covered by at least 10 and at most 150 reads were considered for downstream analyses. Replicates were grouped after filtering for coverage by calculating the arithmetic of the remaining positions given their presence in at least one replicate. Region-specific methylation was assessed by calculating average (arithmetic mean) methylation across features using bigWigAverageOverBed from UCSC tools, where a feature was only considered if at least three CpGs were covered within a region.

### Nucleosome affinity prediction

Nucleosome affinities were calculated using the the predNuPoP_chem() function with default parameters within the NuPoP R package (v2.5). NuPoP uses a hidden Markov model trained on profiles of chemically mapped nucleosomes in mouse embryonic stem cells (57) to predict nucleosome affinities from input DNA sequences over 147 bp sliding windows with 1 bp increments (59). Affinity scores were calculated over 4 kb windows from ATAC peak centers.

### Methylation-sensitive DNA shape prediction

Mean methylation rates of each CpG over a ±1 kb window around the peak centers of long-term memory, short-term memory, resolved and unchanged domains were calculated from replicate-merged bisulfite sequencing bigWig files for each condition. Methylation-annotated fasta (fa) files were then derived over each set of domains (each domain extended ±1 kb from its center), marking “CG” sites with > 50% mean methylation rate as “Mg” to denote methylation. DNA shape predictions were derived from these fasta files using the getShape() function in the DNAshapeR R package (v1.37.0) (96), setting methylate = TRUE and default parameters otherwise.

### Analysis of published ATAC-seq datasets

Bulk ATAC-seq FastQ files were pulled from GSE198564 (acinar cells post-caerulein) (8), GSE165312 (EpdSCs post-wounding) (73), or provided upon request from authors [long-term hematopoietic stem cells (LT-HSCs) post-lipopolysaccharide (LPS)] (13). Raw sequencing reads were aligned to the mouse (mm10) genome, called for peaks, counted over nonredundant, replicable consensus peaks, then run through differential testing using DESeq2, as described above.

For acinar cells post-caerulein, a DESeq2 object was prepared using only samples from naïve, day 2 (D2, peak inflammation), week 3 (Wk3, short-term resolution), and week 18 (Wk18, long-term resolution). As above, memory domains were determined as loci having heightened accessibility in both D2 and Wk3 cells over naïve (DESeq2 *P-adj* < 0.05, log_2_ fold change > 0; 3400 domains). Long-term memory domains were then defined as the subset of memory domains which additionally retained heightened accessibility at Wk18 over naïve cells (179 domains). For comparative purposes, short-term memory domains were conversely defined as an equivalently numbered subset (179 domains) of memory domains with DESeq2 Wald-statistic value closest to 0 at Wk18. Resolved domains were defined as the 178 domains that were induced at D2 and resolved by Wk3 (DESeq2 Wald-statistic closest to 0 in Wk3 versus naïve cells). Unchanged domains were derived as peaks with DESeq2 *P-adj* > 0.2 in all comparisons versus naïve, randomly down-sampled to 179 domains.

For LT-HSCs, memory domains were defined as having heightened accessibility in Wk4 post-LPS over naïve cells (DESeq2 *P-adj* < 0.05, log_2_ fold change > 0; 169 domains). Suppressed domains were conversely defined as having lower accessibility in Wk4 post-LPS cells (DESeq2 *P-adj* < 0.05, log_2_ fold change < 0; 236 domains). As reported, the aggregate accessibility of memory domains remains stable through at least Wk12, while aggregate accessibility of suppressed domains returns to baseline by this time (13). However, Wk4 was chosen for analysis owing to availability of sufficient biological replicates (*n* = 2 per condition). Unchanged domains were derived as peaks with DESeq2 *P-adj* > 0.2 in all comparisons versus naïve, randomly down-sampled to 169 domains.

Wound memory domains were defined as peaks having heightened accessibility in D3 wounded over naïve hair follicle stem cells (HFSCs), which directly respond and fate-switch to EpdSCs in the partial thickness model of wounding used, then sustained in D80 post-wounded EpdSCs (wound HFSC-derived) over D80 naïve EpdSCs (45 domains) (71). Wound-resolved domains were defined as having heightened accessibility in D3 Dremel over D0 HFSC but returned closest to baseline levels of accessibility in D80 Dremel over D80 EpdSC (45 domains with DESeq2 Wald statistics closest to 0). Unchanged domains were derived as peaks with DESeq2 *P-adj* > 0.2 in all comparisons versus naïve, randomly down-sampled to 45 domains.

For all datasets, CpG densities and predicted nucleosome affinities were calculated as described above. Nucleosome affinity predictions for post-wound domains were not included owing to an insufficient number of regions for analysis.

### Visualizations

Violin plots were generated using the vioplot (v0.5.1) package and show the kernel density estimation with embedded boxplots indicating the median, interquartile range, and whiskers extending to 1.5x the inter-quartile range. Pairwise genome-wide correlations of methylation rates between replicates were plotted using the smoothScatter R function. PCAs were calculated using the prcomp R function and visualized using ggplot2 (v3.5.2).

### Statistics

The statistical tests used, methods of error representation, and annotations for significance are provided for each figure in the corresponding legend. Group sizes for wounding, epithelial thickness, EpdSC proliferation, and immune characterization experiments were determined by power analysis on the basis of preliminary experimental results. For all genomics experiments, the replicate numbers, read depth, and quality control assessments followed the ENCODE Consortium’s guidelines and best practices (https://www.encodeproject.org/data-standards/).

## Supplementary Material

Supplementary Material

Data s1

Data s2

Data s3

Data s4

Data s5

Data s6

Data s7

Data s8

Data s9

Data s10

Data s11

Data s12

Data s13

Data s14

Data s15

Data s16

Data s17

Data s18

Data s19

Data s20

Data s21

Data s22

Data s23

science.org/doi/10.1126/science.adz6830

Figs. S1 to S11; Tables S1 and S2; MDAR Reproducibility Checklist; Data S1 to S23

## Data Availability

All data needed to evaluate the conclusions in the paper are present in the paper or the supplementary materials. No new materials were made in this study. ATAC-seq, CNR-seq, MINT-ChIP, RNA-seq, scRNA-seq, scATAC-seq, and WGBS from this study have been deposited in the Gene Expression Omnibus under accession codes GSE299177, GSE299178, GSE300003, GSE300005, GSE299457, GSE301537, and GSE299606. Reanalyzed data from Naik *et al.* (4), Larsen *et al*. (22), Falvo *et al*. (8), and de Laval *et al*. (13) can be accessed under accession codes GSE92967, GSE171596, GSE198564, and GSE143654, respectively. Code for data analysis is publicly available in Zenodo (97).

## References

[1] Foster SL, Hargreaves DC, Medzhitov R, Gene-specific control of inflammation by TLR-induced chromatin modifications. Nature 447, 972–978 (2007). doi: 10.1038/nature05836; 17538624

[2] Quintin J , *Candida albicans* infection affords protection against reinfection via functional reprogramming of monocytes. Cell Host Microbe 12, 223–232 (2012). doi: 10.1016/j.chom.2012.06.006; 22901542 PMC3864037

[3] Kleinnijenhuis J , Bacille Calmette-Guérin induces NOD2-dependent nonspecific protection from reinfection via epigenetic reprogramming of monocytes. Proc. Natl. Acad. Sci. U.S.A. 109, 17537–17542 (2012). doi: 10.1073/pnas.1202870109; 22988082 PMC3491454

[4] Naik S , Inflammatory memory sensitizes skin epithelial stem cells to tissue damage. Nature 550, 475–480 (2017). doi: 10.1038/nature24271; 29045388 PMC5808576

[5] Naik S, Fuchs E, Inflammatory memory and tissue adaptation in sickness and in health. Nature 607, 249–255 (2022). doi: 10.1038/s41586-022-04919-3; 35831602 PMC9302602

[6] Ordovas-Montanes J , Allergic inflammatory memory in human respiratory epithelial progenitor cells. Nature 560, 649–654 (2018). doi: 10.1038/s41586-018-0449-8; 30135581 PMC6133715

[7] Lim AI , Prenatal maternal infection promotes tissue-specific immunity and inflammation in offspring. Science 373, eabf3002 (2021). doi: 10.1126/science.abf3002; 34446580

[8] Falvo DJ , A reversible epigenetic memory of inflammatory injury controls lineage plasticity and tumor initiation in the mouse pancreas. Dev. Cell 58, 2959–2973.e7 (2023). doi: 10.1016/j.devcel.2023.11.008; 38056453 PMC10843773

[9] Zhao D , Inflammation-induced epigenetic imprinting regulates intestinal stem cells. Cell Stem Cell 31, 1447–1464.e6 (2024). doi: 10.1016/j.stem.2024.08.006; 39232559 PMC11963838

[10] Lee H-G , Disease-associated astrocyte epigenetic memory promotes CNS pathology. Nature 627, 865–872 (2024). doi: 10.1038/s41586-024-07187-5; 38509377 PMC11016191

[11] Bian X , Epigenetic memory of radiotherapy in dermal fibroblasts impairs wound repair capacity in cancer survivors. Nat. Commun. 15, 9286 (2024). doi: 10.1038/s41467-024-53295-1; 39468077 PMC11519383

[12] Christ A , Western diet triggers NLRP3-dependent innate immune reprogramming. Cell 172, 162–175.e14 (2018). doi: 10.1016/j.cell.2017.12.013; 29328911 PMC6324559

[13] de Laval B , C/EBPβ-dependent epigenetic memory induces trained immunity in hematopoietic stem cells. Cell Stem Cell 26, 657–674.e8 (2020). doi: 10.1016/j.stem.2020.01.017; 32169166

[14] Del Poggetto E , Epithelial memory of inflammation limits tissue damage while promoting pancreatic tumorigenesis. Science 373, eabj0486 (2021). doi: 10.1126/science.abj0486; 34529467 PMC9733946

[15] Levra Levron C , Tissue memory relies on stem cell priming in distal undamaged areas. Nat. Cell Biol. 25, 740–753 (2023). doi: 10.1038/s41556-023-01120-0; 37081165 PMC10185470

[16] Netea MG, Quintin J, van der Meer JWM, Trained immunity: A memory for innate host defense. Cell Host Microbe 9, 355–361 (2011). doi: 10.1016/j.chom.2011.04.006; 21575907

[17] Tian D, Lai Y, The relapse of psoriasis: Mechanisms and mysteries. JID Innov. 2, 100116 (2022). doi: 10.1016/j.xjidi.2022.100116; 35601055 PMC9121322

[18] Kirkegård J, Cronin-Fenton D, Heide-Jørgensen U, Mortensen FV, Acute pancreatitis and pancreatic cancer risk: A nationwide matched-cohort study in Denmark. Gastroenterology 154, 1729–1736 (2018). doi: 10.1053/j.gastro.2018.02.011; 29432727

[19] Benn CS, Netea MG, Selin LK, Aaby P, A small jab – a big effect: Nonspecific immunomodulation by vaccines. Trends Immunol. 34, 431–439 (2013). doi: 10.1016/j.it.2013.04.004; 23680130

[20] Kleinnijenhuis J , Long-lasting effects of BCG vaccination on both heterologous Th1/Th17 responses and innate trained immunity. J. Innate Immun. 6, 152–158 (2014). doi: 10.1159/000355628; 24192057 PMC3944069

[21] Rückert T, Lareau CA, Mashreghi M-F, Ludwig LS, Romagnani C, Clonal expansion and epigenetic inheritance of long-lasting NK cell memory. Nat. Immunol. 23, 1551–1563 (2022). doi: 10.1038/s41590-022-01327-7; 36289449 PMC9663309

[22] Larsen SB , Establishment, maintenance, and recall of inflammatory memory. Cell Stem Cell 28, 1758–1774.e8 (2021). doi: 10.1016/j.stem.2021.07.001; 34320411 PMC8500942

[23] Vierbuchen T , AP-1 transcription factors and the BAF complex mediate signal-dependent enhancer selection. Mol. Cell 68, 1067–1082.e12 (2017). doi: 10.1016/j.molcel.2017.11.026; 29272704 PMC5744881

[24] Barral A, Zaret KS, Pioneer factors: Roles and their regulation in development. Trends Genet. 40, 134–148 (2024). doi: 10.1016/j.tig.2023.10.007; 37940484 PMC10873006

[25] Sada A , Defining the cellular lineage hierarchy in the interfollicular epidermis of adult skin. Nat. Cell Biol. 18, 619–631 (2016). doi: 10.1038/ncb3359; 27183471 PMC4884151

[26] Tamoutounour S , Keratinocyte-intrinsic MHCII expression controls microbiota-induced Th1 cell responses. Proc. Natl. Acad. Sci. U.S.A. 116, 23643–23652 (2019). doi: 10.1073/pnas.1912432116; 31672911 PMC6876208

[27] Chen YE, Fischbach MA, Belkaid Y, Skin microbiota–host interactions. Nature 553, 427–436 (2018). doi: 10.1038/nature25177; 29364286 PMC6075667

[28] van der Fits L , Imiquimod-induced psoriasis-like skin inflammation in mice is mediated via the IL-23/IL-17 axis. J. Immunol. 182, 5836–5845 (2009). doi: 10.4049/jimmunol.0802999; 19380832

[29] Meers MP, Bryson TD, Henikoff JG, Henikoff S, Improved CUT&RUN chromatin profiling tools. eLife 8, e46314 (2019). doi: 10.7554/eLife.46314; 31232687 PMC6598765

[30] van Galen P , A multiplexed system for quantitative comparisons of chromatin landscapes. Mol. Cell 61, 170–180 (2016). doi: 10.1016/j.molcel.2015.11.003; 26687680 PMC4707994

[31] Keyes BE , Impaired epidermal to dendritic T cell signaling slows wound repair in aged skin. Cell 167, 1323–1338.e14 (2016). doi: 10.1016/j.cell.2016.10.052; 27863246 PMC5364946

[32] Ge Y , The aging skin microenvironment dictates stem cell behavior. Proc. Natl. Acad. Sci. U.S.A. 117, 5339–5350 (2020). doi: 10.1073/pnas.1901720117; 32094197 PMC7071859

[33] Solá P , Targeting lymphoid-derived IL-17 signaling to delay skin aging. Nat. Aging 3, 688–704 (2023). doi: 10.1038/s43587-023-00431-z; 37291218 PMC10275755

[34] Holt DR , Effect of age on wound healing in healthy human beings. Surgery 112, 293–297 (1992). 1641768

[35] Mitroulis I , Modulation of myelopoiesis progenitors is an integral component of trained immunity. Cell 172, 147–161.e12 (2018). doi: 10.1016/j.cell.2017.11.034; 29328910 PMC5766828

[36] Gerber PA , Systematic identification and characterization of novel human skin-associated genes encoding membrane and secreted proteins. PLOS ONE 8, e63949 (2013). doi: 10.1371/journal.pone.0063949; 23840300 PMC3688712

[37] Zhang Z , CYP7B1-mediated 25-hydroxycholesterol degradation maintains quiescence-activation balance and improves therapeutic potential of mesenchymal stem cells. Cell Chem. Biol. 31, 1277–1289.e7 (2024). doi: 10.1016/j.chembiol.2024.01.009; 38382532

[38] Gunyuz ZE , SEMA6D differentially regulates proliferation, migration, and invasion of breast cell lines. ACS Omega 7, 15769–15778 (2022). doi: 10.1021/acsomega.2c00840; 35571788 PMC9097209

[39] Thomas HF , Enhancer cooperativity can compensate for loss of activity over large genomic distances. Mol. Cell 85, 362–375.e9 (2025). doi: 10.1016/j.molcel.2024.11.008; 39626663

[40] Koren E , Thy1 marks a distinct population of slow-cycling stem cells in the mouse epidermis. Nat. Commun. 13, 4628 (2022). doi: 10.1038/s41467-022-31629-1; 35941116 PMC9360001

[41] Asare A, Levorse J, Fuchs E, Coupling organelle inheritance with mitosis to balance growth and differentiation. Science 355, eaah4701 (2017). doi: 10.1126/science.aah4701; 28154022 PMC5333555

[42] Persad S , SEACells infers transcriptional and epigenomic cellular states from single-cell genomics data. Nat. Biotechnol. 41, 1746–1757 (2023). doi: 10.1038/s41587-023-01716-9; 36973557 PMC10713451

[43] Schep AN, Wu B, Buenrostro JD, Greenleaf WJ, chromVAR: Inferring transcription-factor-associated accessibility from single-cell epigenomic data. Nat. Methods 14, 975–978 (2017). doi: 10.1038/nmeth.4401; 28825706 PMC5623146

[44] Granja JM , ArchR is a scalable software package for integrative single-cell chromatin accessibility analysis. Nat. Genet. 53, 403–411 (2021). doi: 10.1038/s41588-021-00790-6; 33633365 PMC8012210

[45] Weirauch MT , Determination and inference of eukaryotic transcription factor sequence specificity. Cell 158, 1431–1443 (2014). doi: 10.1016/j.cell.2014.08.009; 25215497 PMC4163041

[46] Pampari A , ChromBPNet: bias factorized, base-resolution deep learning models of chromatin accessibility reveal cis-regulatory sequence syntax, transcription factor footprints and regulatory variants. bioRxiv 2024.12.25.630221 [Preprint] (2025); 10.1101/2024.12.25.630221.

[47] Avsec Ž , Base-resolution models of transcription-factor binding reveal soft motif syntax. Nat. Genet. 53, 354–366 (2021). doi: 10.1038/s41588-021-00782-6; 33603233 PMC8812996

[48] Chodavarapu RK , Relationship between nucleosome positioning and DNA methylation. Nature 466, 388–392 (2010). doi: 10.1038/nature09147; 20512117 PMC2964354

[49] Choy JS , DNA methylation increases nucleosome compaction and rigidity. J. Am. Chem. Soc. 132, 1782–1783 (2010). doi: 10.1021/ja910264z; 20095602 PMC4167393

[50] Yin Y , Impact of cytosine methylation on DNA binding specificities of human transcription factors. Science 356, eaaj2239 (2017). doi: 10.1126/science.aaj2239; 28473536 PMC8009048

[51] Domcke S , Competition between DNA methylation and transcription factors determines binding of NRF1. Nature 528, 575–579 (2015). doi: 10.1038/nature16462; 26675734

[52] Sardina JL , Transcription factors drive Tet2-mediated enhancer demethylation to reprogram cell fate. Cell Stem Cell 23, 727–741.e9 (2018). doi: 10.1016/j.stem.2018.08.016; 30220521

[53] Zilberman D, Coleman-Derr D, Ballinger T, Henikoff S, Histone H2A.Z and DNA methylation are mutually antagonistic chromatin marks. Nature 456, 125–129 (2008). doi: 10.1038/nature07324; 18815594 PMC2877514

[54] Mattei AL, Bailly N, Meissner A, DNA methylation: A historical perspective. Trends Genet. 38, 676–707 (2022). doi: 10.1016/j.tig.2022.03.010; 35504755

[55] Polansky JK , Methylation matters: Binding of Ets-1 to the demethylated *Foxp3* gene contributes to the stabilization of Foxp3 expression in regulatory T cells. J. Mol. Med. 88, 1029–1040 (2010). doi: 10.1007/s00109-010-0642-1; 20574810 PMC2943068

[56] Kaplan N , The DNA-encoded nucleosome organization of a eukaryotic genome. Nature 458, 362–366 (2009). doi: 10.1038/nature07667; 19092803 PMC2658732

[57] Voong LN , Insights into nucleosome organization in mouse embryonic stem cells through chemical mapping. Cell 167, 1555–1570.e15 (2016). doi: 10.1016/j.cell.2016.10.049; 27889238 PMC5135608

[58] Xi Y, Yao J, Chen R, Li W, He X, Nucleosome fragility reveals novel functional states of chromatin and poises genes for activation. Genome Res. 21, 718–724 (2011). doi: 10.1101/gr.117101.110; 21363969 PMC3083088

[59] Xi L , Predicting nucleosome positioning using a duration Hidden Markov Model. BMC Bioinformatics 11, 346–346 (2010). doi: 10.1186/1471-2105-11-346; 20576140 PMC2900280

[60] Ngo TTM, Zhang Q, Zhou R, Yodh JG, Ha T, Asymmetric unwrapping of nucleosomes under tension directed by DNA local flexibility. Cell 160, 1135–1144 (2015). doi: 10.1016/j.cell.2015.02.001; 25768909 PMC4409768

[61] Jin C , H3.3/H2A.Z double variant-containing nucleosomes mark ‘nucleosome-free regions’ of active promoters and other regulatory regions. Nat. Genet. 41, 941–945 (2009). doi: 10.1038/ng.409; 19633671 PMC3125718

[62] Placek BJ, Harrison LN, Villers BM, Gloss LM, The H2A.Z/H2B dimer is unstable compared to the dimer containing the major H2A isoform. Protein Sci. 14, 514–522 (2005). doi: 10.1110/ps.041026405; 15632282 PMC2253418

[63] Hu G , H2A.Z facilitates access of active and repressive complexes to chromatin in embryonic stem cell self-renewal and differentiation. Cell Stem Cell 12, 180–192 (2013). doi: 10.1016/j.stem.2012.11.003; 23260488 PMC3570599

[64] Li S, Wei T, Panchenko AR, Histone variant H2A.Z modulates nucleosome dynamics to promote DNA accessibility. Nat. Commun. 14, 769 (2023). doi: 10.1038/s41467-023-36465-5; 36765119 PMC9918499

[65] Shih RM, Arimura Y, Konishi HA, Funabiki H, Impacts of DNA methylation on H2A.Z deposition and nucleosome stability. bioRxiv 2025.07.31.667981 [Preprint] (2025); 10.1101/2025.07.31.667981. PMC13341117 42411467

[66] Conerly ML , Changes in H2A.Z occupancy and DNA methylation during B-cell lymphomagenesis. Genome Res. 20, 1383–1390 (2010). doi: 10.1101/gr.106542.110; 20709945 PMC2945187

[67] Murphy PJ, Wu SF, James CR, Wike CL, Cairns BR, Placeholder nucleosomes underlie germline-to-embryo DNA methylation reprogramming. Cell 172, 993–1006.e13 (2018). doi: 10.1016/j.cell.2018.01.022; 29456083

[68] Albert I , Translational and rotational settings of H2A.Z nucleosomes across the *Saccharomyces cerevisiae* genome. Nature 446, 572–576 (2007). doi: 10.1038/nature05632; 17392789

[69] Flury V , Recycling of modified H2A-H2B provides short-term memory of chromatin states. Cell 186, 1050–1065.e19 (2023). doi: 10.1016/j.cell.2023.01.007; 36750094 PMC9994263

[70] Kikuchi M , GAS41 promotes H2A.Z deposition through recognition of the N terminus of histone H3 by the YEATS domain. Proc. Natl. Acad. Sci. U.S.A. 120, e2304103120 (2023). doi: 10.1073/pnas.2304103120; 37844223 PMC10614846

[71] Gonzales KAU , Stem cells expand potency and alter tissue fitness by accumulating diverse epigenetic memories. Science 374, eabh2444 (2021). doi: 10.1126/science.abh2444; 34822296 PMC8896201

[72] Cheong J-G , Epigenetic memory of coronavirus infection in innate immune cells and their progenitors. Cell 186, 3882–3902.e24 (2023). doi: 10.1016/j.cell.2023.07.019; 37597510 PMC10638861

[73] Sun SJ , BCG vaccination alters the epigenetic landscape of progenitor cells in human bone marrow to influence innate immune responses. Immunity 57, 2095–2107.e8 (2024). doi: 10.1016/j.immuni.2024.07.021; 39153479 PMC11604037

[74] March-Díaz R , Histone H2A.Z and homologues of components of the SWR1 complex are required to control immunity in Arabidopsis. Plant J. 53, 475–487 (2008). doi: 10.1111/j.1365-313X.2007.03361.x; 17988222

[75] Buenrostro JD, Giresi PG, Zaba LC, Chang HY, Greenleaf WJ, Transposition of native chromatin for fast and sensitive epigenomic profiling of open chromatin, DNA-binding proteins and nucleosome position. Nat. Methods 10, 1213–1218 (2013). doi: 10.1038/nmeth.2688; 24097267 PMC3959825

[76] Frankish A , GENCODE: Reference annotation for the human and mouse genomes in 2023. Nucleic Acids Res. 51, D942–D949 (2023). doi: 10.1093/nar/gkac1071; 36420896 PMC9825462

[77] Li H, Durbin R, Fast and accurate short read alignment with Burrows–Wheeler transform. Bioinformatics 25, 1754–1760 (2009). doi: 10.1093/bioinformatics/btp324; 19451168 PMC2705234

[78] Danecek P , Twelve years of SAMtools and BCFtools. Gigascience 10, giab008 (2021). doi: 10.1093/gigascience/giab008; 33590861 PMC7931819

[79] Ramírez F , deepTools2: A next generation web server for deep-sequencing data analysis. Nucleic Acids Res. 44, W160–W165 (2016). doi: 10.1093/nar/gkw257; 27079975 PMC4987876

[80] Zhang Y , Model-based analysis of ChIP-Seq (MACS). Genome Biol. 9, R137 (2008). doi: 10.1186/gb-2008-9-9-r137; 18798982 PMC2592715

[81] Love MI, Huber W, Anders S, Moderated estimation of fold change and dispersion for RNA-seq data with DESeq2. Genome Biol. 15, 550 (2014). doi: 10.1186/s13059-014-0550-8; 25516281 PMC4302049

[82] Fornes O , JASPAR 2020: Update of the open-access database of transcription factor binding profiles. Nucleic Acids Res. 48, D87–D92 (2020). doi: 10.1093/nar/gkz1001; 31701148 PMC7145627

[83] Schep A, motifmatchr: Fast Motif Matching in R, R package version 1.30.0 (2025); https://bioconductor.org/packages/motifmatchr.

[84] Wolf FA, Angerer P, Theis FJ, SCANPY: Large-scale single-cell gene expression data analysis. Genome Biol. 19, 15 (2018). doi: 10.1186/s13059-017-1382-0; 29409532 PMC5802054

[85] Zheng GXY , Massively parallel digital transcriptional profiling of single cells. Nat. Commun. 8, 14049 (2017). doi: 10.1038/ncomms14049; 28091601 PMC5241818

[86] Fleming SJ , Unsupervised removal of systematic background noise from droplet-based single-cell experiments using CellBender. Nat. Methods 20, 1323–1335 (2023). doi: 10.1038/s41592-023-01943-7; 37550580

[87] Levine JH , Data-driven phenotypic dissection of AML reveals progenitor-like cells that correlate with prognosis. Cell 162, 184–197 (2015). doi: 10.1016/j.cell.2015.05.047; 26095251 PMC4508757

[88] Shrikumar A, Greenside P, Kundaje A, Learning important features through propagating activation differences. arXiv:1704.02685 [cs.CV] (2017).

[89] Shrikumar A , Technical Note on Transcription Factor Motif Discovery from Importance Scores (TF-MoDISco) version 0.5.6.5. arXiv:1811.00416 [cs.LG] (2020).

[90] Sandelin A, Alkema W, Engström P, Wasserman WW, Lenhard B, JASPAR: An open-access database for eukaryotic transcription factor binding profiles. Nucleic Acids Res. 32, D91–D94 (2004). doi: 10.1093/nar/gkh012; 14681366 PMC308747

[91] Kulakovskiy IV , HOCOMOCO: Towards a complete collection of transcription factor binding models for human and mouse via large-scale ChIP-Seq analysis. Nucleic Acids Res. 46, D252–D259 (2018). doi: 10.1093/nar/gkx1106; 29140464 PMC5753240

[92] Gupta S, Stamatoyannopoulos JA, Bailey TL, Noble WS, Quantifying similarity between motifs. Genome Biol. 8, R24 (2007). doi: 10.1186/gb-2007-8-2-r24; 17324271 PMC1852410

[93] Subramanian A , Gene set enrichment analysis: A knowledge-based approach for interpreting genome-wide expression profiles. Proc. Natl. Acad. Sci. U.S.A. 102, 15545–15550 (2005). doi: 10.1073/pnas.0506580102; 16199517 PMC1239896

[94] Yu G, Wang L-G, Han Y, He Q-Y, clusterProfiler: An R package for comparing biological themes among gene clusters. OMICS 16, 284–287 (2012). doi: 10.1089/omi.2011.0118; 22455463 PMC3339379

[95] McKenna A , The Genome Analysis Toolkit: A MapReduce framework for analyzing next-generation DNA sequencing data. Genome Res. 20, 1297–1303 (2010). doi: 10.1101/gr.107524.110; 20644199 PMC2928508

[96] Chiu T-P , DNAshapeR: An R/Bioconductor package for DNA shape prediction and feature encoding. Bioinformatics 32, 1211–1213 (2016). doi: 10.1093/bioinformatics/btv735; 26668005 PMC4824130

[97] Soto Ugaldi LF, dpeerlab/persistent-epigenetic-memory, version 0.1.1, Zenodo (2025); 10.5281/zenodo.18057635.

