# Supplementary material for: Distinctive DNA sequence features define epigenetic longevity of inflammatory memory
Source: Science. Author manuscript; Available in PMC 2026 Jun 26. (PMC13295011; doi:10.1126/science.adz6830)
Supplement: Supplementary Material [file NIHMS2170334-supplement-Supplementary_Material.pdf]

Science
AAAS

Supplementary Materials for

Distinctive DNA sequence features define epigenetic longevity of inflammatory memory

Christopher J. Cowley *et al*.

Corresponding authors: Dana Pe'er, peerd@mskcc.org; Elaine Fuchs, fuchslb@rockefeller.edu

Science **391**, eadz6830 (2026)
DOI: 10.1126/science.adz6830

The PDF file includes:

Figs. S1 to S11
Tables S1 and S2

Other Supplementary Material for this manuscript includes the following:

MDAR Reproducibility Checklist
Data S1 to S23

Sup. Figure 1: Epidermal stem cells maintain epigenetic inflammatory memory throughout murine life.

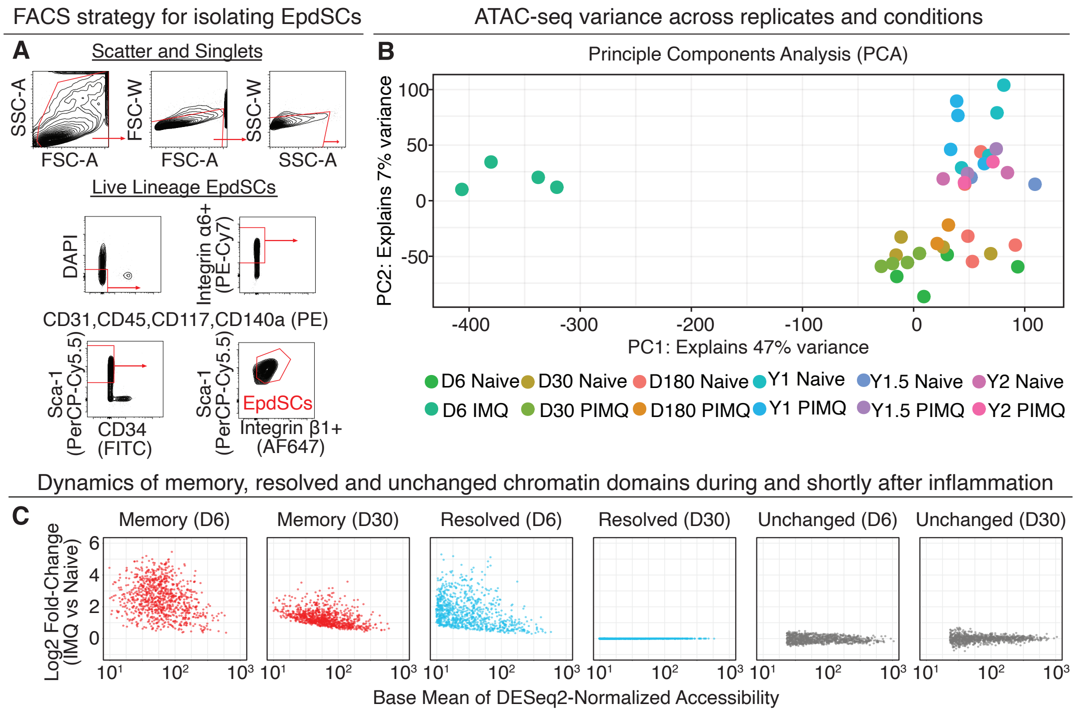

Fig. S1. Epidermal stem cells maintain epigenetic inflammatory memory throughout murine life.

(**A**) Strategy to isolate EpdSCs from mouse skins by fluorescence-activated cell sorting (FACS). Side scatter area (SSC-A), forward scatter area (FSC-A), side scatter width (SSC-W), forward scatter width (FSC-W). (**B**) Principal components analysis (PCA) of inter-sample variance over consensus peak set derived from shared peaks across all ATAC-seq samples in Fig. 1A,B. (**C**) Log_2 fold-change against average intensity (‘Minus-Average’, MA) plot used to visualize differences in accessibility (Log_2 ratio between IMQ over naïve EpdSCs) against mean accessibilities across samples within memory (n = 934 domains), resolved (n = 933 domains) and unchanged chromatin domains (n = 934 domains) from Fig. 1B during the height of inflammation (D6) and upon early resolution (D30) in EpdSCs.

Sup. Figure 2: Inflammatory memory domains are maintained independently of age-related changes to the skin.

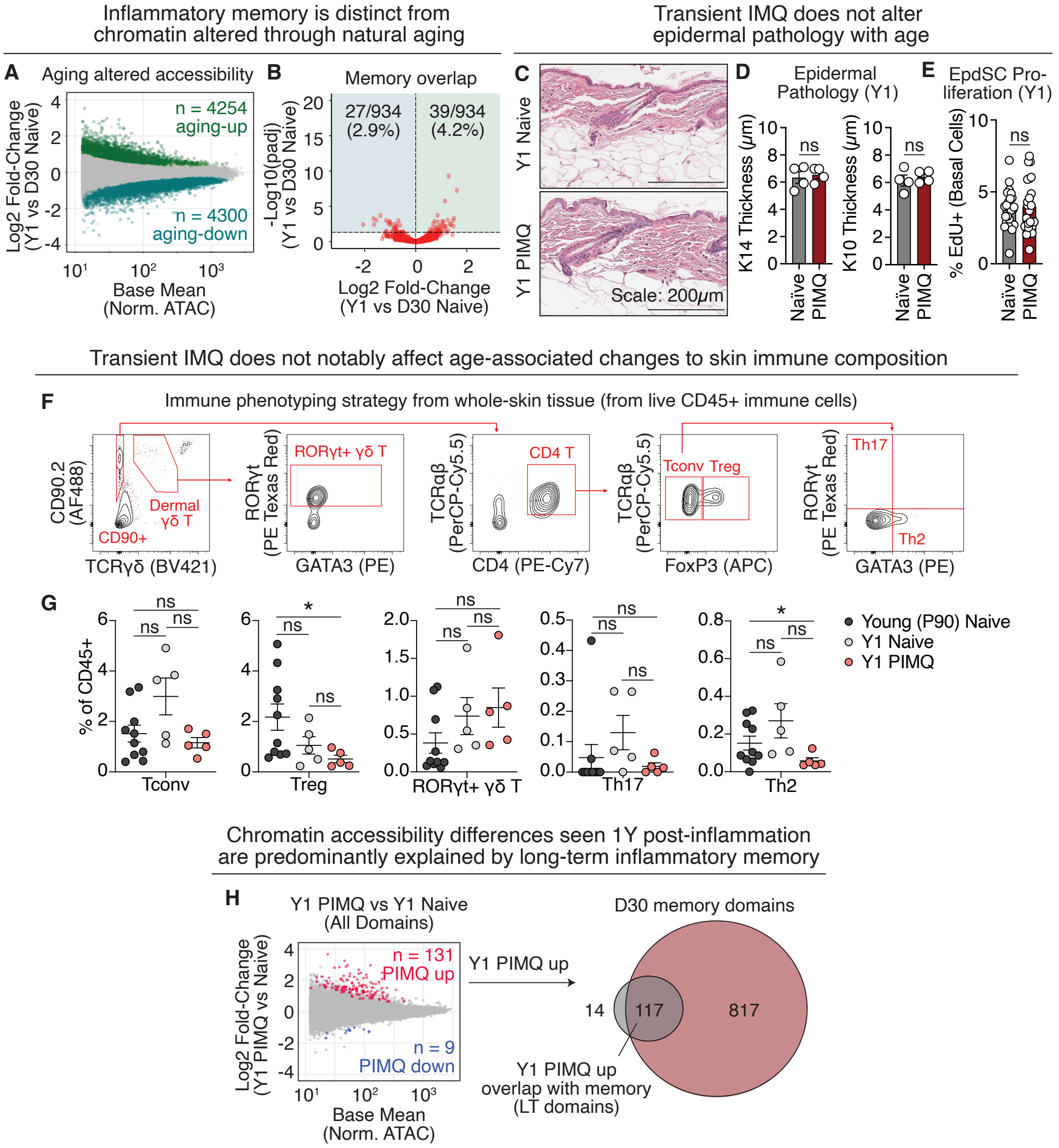

Fig. S2. Inflammatory memory domains are maintained independently of age-related changes to the skin.

(A) Log_2 fold-change against average intensity (MA) plot showing chromatin domains naturally opened in EpdSCs during aging (Y1 over D30 naïve; DESeq2 *P-adj* < 0.05). **(B)** Volcano plot showing age-related dynamics of the 934 inflammatory memory domains from Fig. 1B. Shaded boxes and percentages indicate overlap of memory domains with domains associated with aging. Note that only 4.2% of all memory domains show signs of chromatin accessibility in naïve aged skin, underscoring the specificity of memory domains to prior acute inflammation. **(C)** Hematoxylin and Eosin staining of Y1 naïve and PIMQ sagittal skin sections. Note similar tissue morphologies between Y1 naïve and PIMQ skins. **(D)** Quantification of immunofluorescence imaging depicting epidermal basal (K14) and differentiated (K10) layer thickness between Y1 naïve and PIMQ skins. **(E)** Quantification of EpdSC proliferation (S-phase EdU labeling) in Y1 naïve and PIMQ skins. **(F)** Flow cytometry strategy used to identify skin immune populations. **(G)** Flow cytometric analysis of back skin immune composition in naïve young-adult, naïve aged (Y1) and Y1 post-IMQ mice. *$P < 0.05$; ns, not significant [two-tailed unpaired Student's *t* test]. (D to G) Data represent two independent experiments. **(H)** Left, MA plot showing differential ATAC-seq peaks between Y1 PIMQ over Y1 naïve EpdSC chromatin. Right, Overlap between the 934 memory domains from Fig. 1B and the 131 peaks increased in Y1 PIMQ. Note that the 117 overlapping domains represent the long-term inflammatory memory domains from Fig 1D.

Sup. Figure 3: Genomic context and functionality of inflammatory memory domains.

Inflammatory memory domains predominantly comprise intronic and intergenic cis-regulatory elements

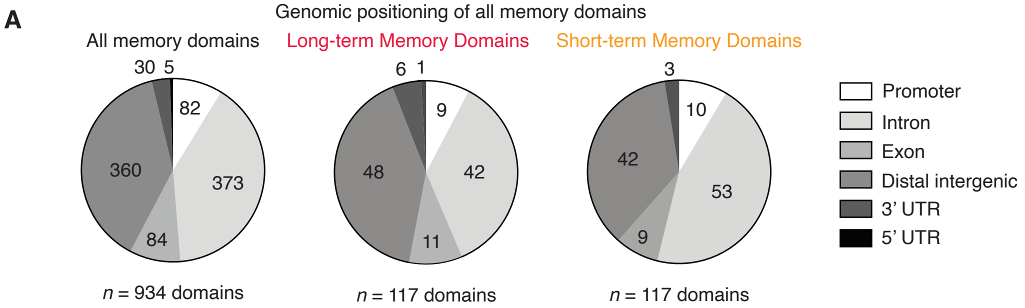

Nearly all memory domains accelerate recruitment of c-FOS upon secondary stress at D30 PIMQ

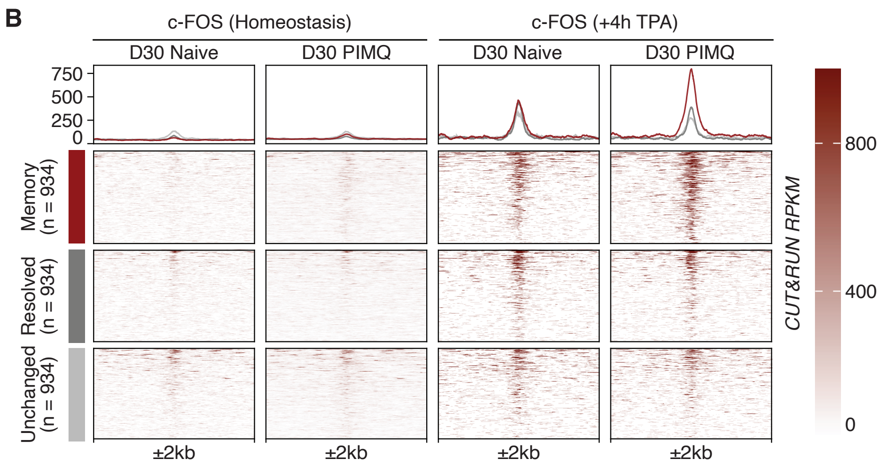

Fig. S3. Genomic context and functionality of inflammatory memory domains.
(A) Genomic annotation of all identified memory domains (n = 934), short-term (ST) (n = 117) and long-term (LT) (n = 117) memory domains, categorized according to their overlap with genomic features including promoters, enhancers, intergenic regions and gene bodies. **(B)** Left, RPKM-normalized signal of c-FOS binding during naïve and PIMQ homeostasis, or upon secondary challenge by TPA at D30 across all memory, resolved and unchanged domains. Signal intensities were averaged across biological replicates. Note the selective reengagement of c-FOS at memory domains upon re-stimulation.

Sup. Figure 4: Single-cell RNA-seq expounds widespread inflammatory resolution across the basal epidermis.

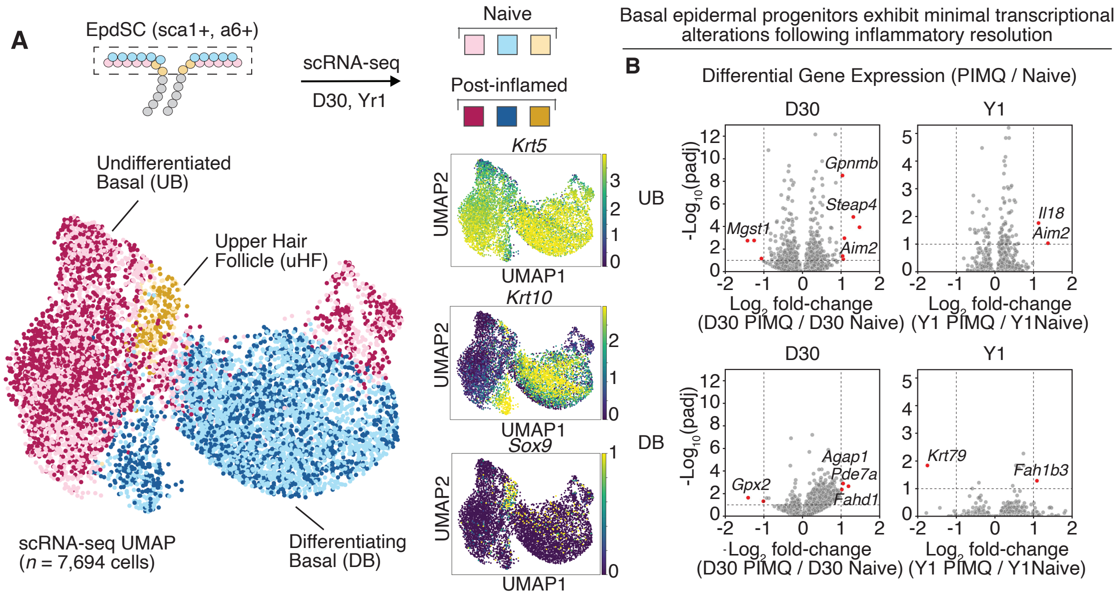

Fig. S4. Single-cell RNA-seq expounds widespread inflammatory resolution across the basal epidermis.

(A) Left, UMAP embedding of scRNA-seq data from EpdSCs collected at D30 or Y1 under PIMQ and naïve conditions. PIMQ cells are represented in darker shades of the corresponding naïve cell colors. Right, single-cell RNA expression of marker genes used to annotate the three main populations within epidermal basal layer: undifferentiated basal (UB, bona fide interfollicular EpdSCs), differentiating basal (DB, interfollicular EpdSC progeny prior to departing the basal layer and undergoing terminal differentiation), and EpdSCs surrounding the upper hair follicle (uHF) orifice. Data represent one experiment. **(B)** Volcano plots showing differentially expressed genes in the UB (top) and DB (bottom) population, as identified using 'diffxpy' comparing D30 PIMQ vs. naïve (left) and Y1 PIMQ vs. naïve (right). Differential genes are indicated as red dots (*P-adj* < 0.10, $|\text{Log}_2$ fold change$| > 1$), with representative genes labeled. uHF was not shown due to insufficient cell number and read coverage for differential gene expression analysis. Note that transcripts largely return to naïve levels post-inflammation.

Sup. Figure 5: Single-cell ATAC-seq characterization of epigenetic inflammatory memory.

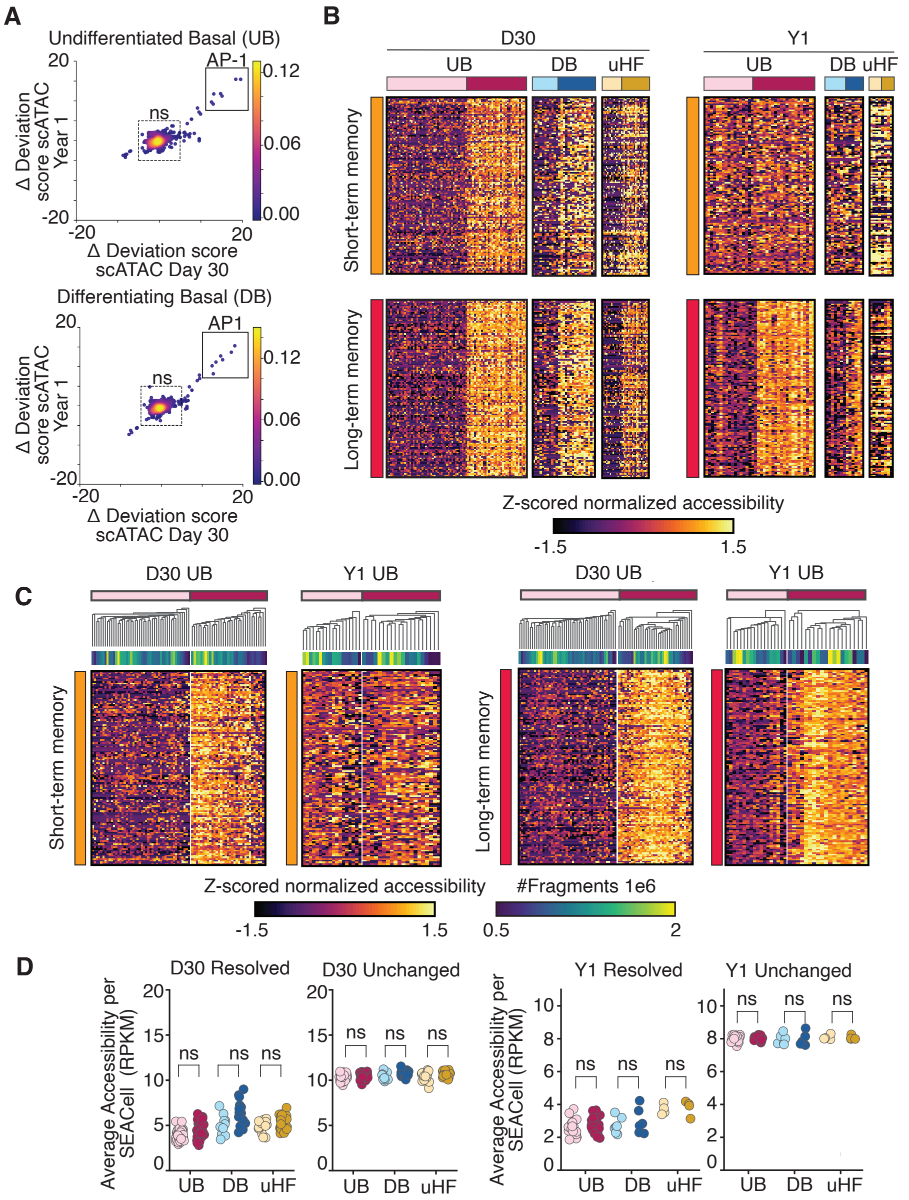

Fig. S5. Single-cell ATAC-seq characterization of epigenetic inflammatory memory.
(A) Scatterplots illustrating differences in the genome-wide accessibilities of canonical transcription factor (TF) motifs between PIMQ and naïve SEACells, calculated using the ChromVAR algorithm *(43)* for D30 (x-axis) and Y1 (y-axis). Colors reflect density of motifs around specific x- and y-coordinates of the plot. Each point represents one TF motif from the CISBP database. Motifs of TFs within the dashed rectangles exhibit non-significant difference between PIMQ and naïve in either time point, while those outside show significant difference at D30 and/or Y1. Note that almost all motifs are comparably accessible between PIMQ and naïve conditions either at D30 or Y1, with the prominent exception of AP1 motifs (non-dashed rectangle). Top, Differential motif accessibility in PIMQ versus naïve UB cells. Bottom, Differential motif accessibility in PIMQ versus naïve DB cells. *P-adj* < 0.001 was used to call significant motifs [two-tailed unpaired Student's *t*-test]. ns, not significant. **(B)** Heatmaps displaying relative (z-scored) intensity of RPKM-normalized chromatin accessibility signals across ST (top, orange) and LT (bottom, red) memory domains at the indicated timepoints. Each column corresponds to a single SEACell, and each row to one memory domain. SEACells are color-coded by basal cell population, with PIMQ in dark and age-matched naïve controls in light colors. *Z*-scores were calculated independently for each time point across memory domains. **(C)** Hierarchical clustering of heatmap depicting ST and LT memory signal in UB (see Fig. 3C), with fragments captured per SEACell annotated as blue-yellow bar. Note that the apparent structure in memory domain accessibility closely follows overall sequencing depth, and thus likely reflects known depth-driven effects in sparse scATAC-seq data. **(D)** Average chromatin accessibility per SEACell, normalized using reads per kilobase per million (RPKM), for resolved and unchanged domains across UB, DB and uHF cells from D30 (left) and Y1 (right).

Sup. Figure 6: Long- and short-term memory domains are not distinguished by known regulators of memory establishment during acute inflammation.

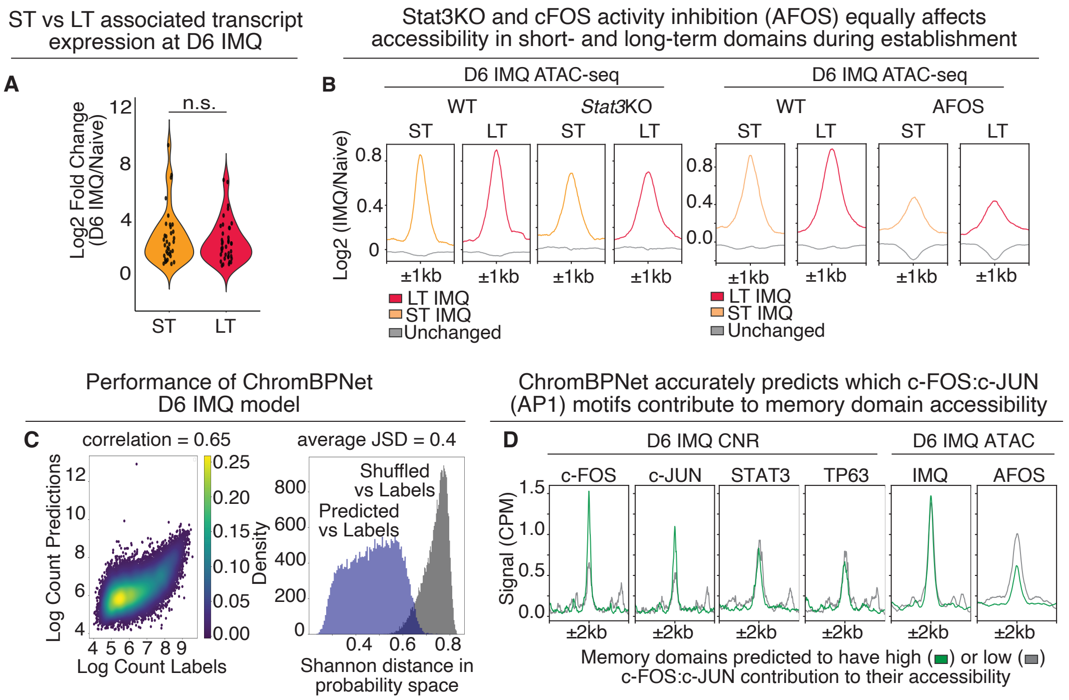

Fig. S6. Long- and short-term memory domains are not distinguished by known regulators of memory establishment during acute inflammation.

(**A**) Log_2 fold-changes in inflammation-induced transcription (D6 IMQ) of closest-proximity genes to ST and LT memory domains. ns, not significant [two-tailed unpaired Student's *t*-test.] (**B**) At the height of inflammation, loss of STAT3 (left) or AP1 (right) comparably impairs establishment of both long- and short-term memory domains. Log_2 ratio of D6 IMQ over naïve ATAC-seq signal within unchanged, ST and LT domains. *Stat3* was conditionally targeted for ablation in EpdSCs prior to IMQ treatment (*Stat3*KO) (*22*). AFOS (dominant-negative c-FOS) was conditionally induced in the basal epidermis prior to IMQ treatment (*22*). **(C)** Performance of the ChromBPNet model trained on D6 IMQ ATAC data. Left, Scatterplot showing the correlation between predicted and observed log-transformed accessibility counts per region. Right, JensenShannon Distance (JSD) between predicted and observed chromatin accessibility profiles shows strong similarity across regions. **(D)** Left, D6 IMQ CPM-normalized CNR signals for c-JUN, cFOS, STAT3 and TP63 plotted over memory domains that exclusively contain either high-contribution AP1 motifs (motifs with top 20% contribution scores across all AP1 instances in memory domains) or low-contribution AP1 motifs (bottom 20%). c-JUN and c-FOS binding is increased in high contributing motifs with no difference observed with STAT3 and TP63. Right, CPM-normalized ATAC-seq signal in D6 IMQ and D6 IMQ with AFOS EpdSCs, plotted over memory domains exclusively containing either high- or low- contribution AP1 motifs. AP1 inhibition though AFOS results in greater loss of accessibility above high- compared to low-contribution motifs. See 'Methods' for full analysis details.

Sup. Figure 7: PersistNet; A BPNet-based model to discover DNA sequence patterns underlying the propagation of epigenetic memory over time.

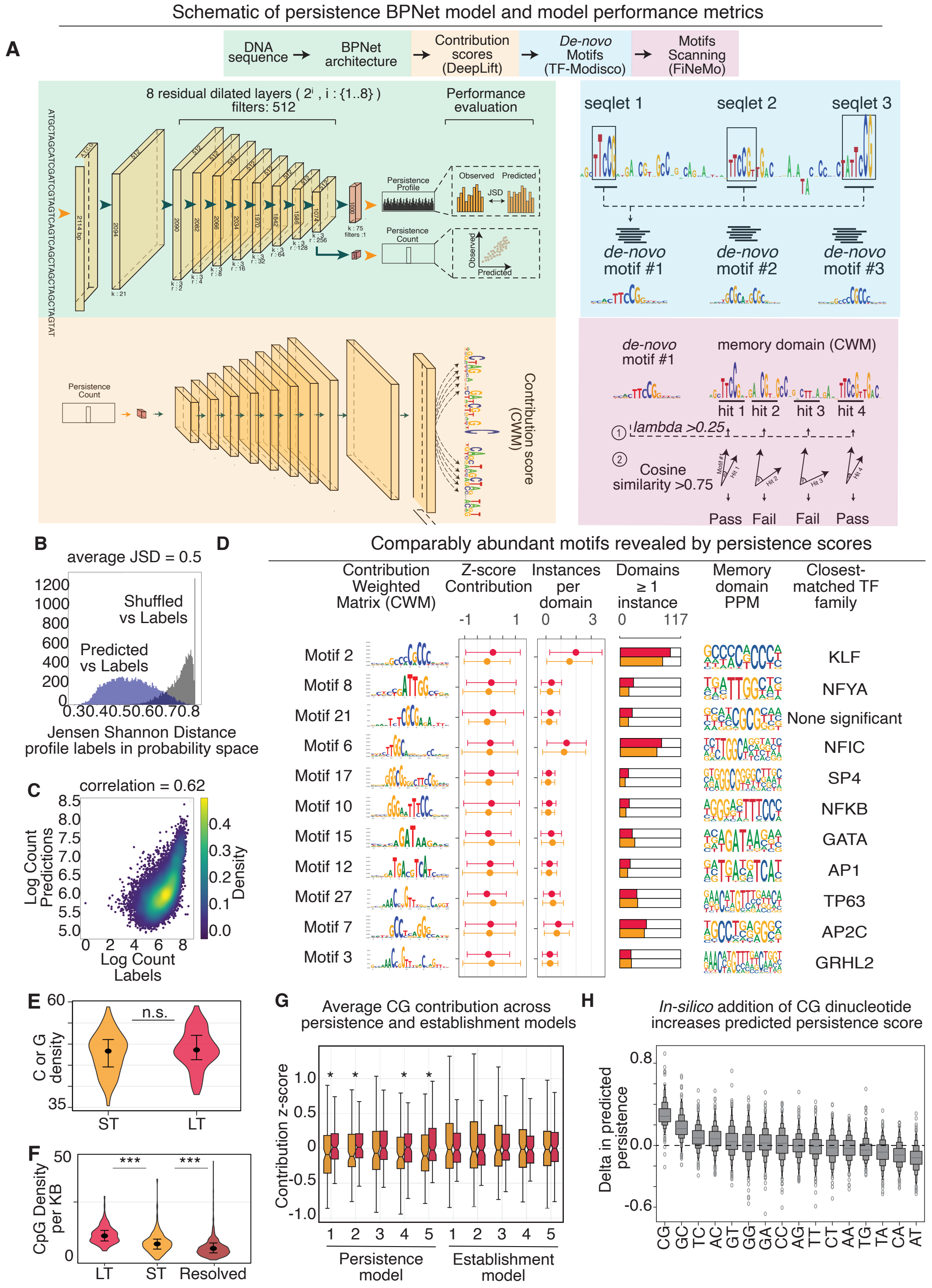

Fig. S7. PersistNet; A BPNet-based model to discover DNA sequence patterns underlying the propagation of epigenetic memory over time.
(A) Schematic of the model used to predict two heads: persistence scores (counts) and persistence distributions (profile) from DNA sequences (green). The PersistNet model was trained on Y1 PIMQ and D30 PIMQ genome-wide datasets to recognize inflammation-induced ATAC peaks that remained in an accessible state over time. A counts head was then used to calculate per-base contribution using the DeepLift algorithm (yellow), from which 'SeqLets' (regions with high contribution scores) were clustered to generate de novo motifs using TF-Modisco (blue). These de novo motifs were then scanned within our memory domains to detect potential hits using two filtering steps: a lambda threshold > 0.25, and cosine similarity > 0.75 (red). **(B, C)** Performance evaluation of the PersistNet model. **(B)** The Jensen-Shannon Distance (JSD) between predicted and observed chromatin accessibility profiles, showing similarity across regions compared to JSD obtained from random shuffling of regions. **(C)** Scatterplot showing the correlation between predicted and observed log-transformed persistence counts per region. Pearson correlation is shown on top. **(D)** De novo motif discovery from PersistNet model. Left to right, Contribution-weighted matrices (CWM) derived from genome-wide motif instances; Z-score normalized contribution score averaged across all instances for each memory domain, number of instances within each memory domain; number of domains with at least one instance; position probability matrices (PPM) created from all instances of the motifs within memory domains; closest match of instances within memory domains with a known TF motif based on positionweighted matrices. Closest TF family for each de novo motif is reported with q-adjusted reported from TOMTOM (see 'Methods' for annotation and statistical testing of de novo sequences to known TF motifs). (**E**) Violin plot of single C or G nucleotide density in ST and LT memory domains. (**F**) Violin plot of CpG density per kilobase (Kb) in ST, LT and resolved domains. **(G)** Average CG contribution to accessibility in LT and ST domains across five independent persistence and establishment models. *$P < 0.05$ [Mann-Whitney U test]. **(H)** Predicted persistence scores for the same wild-type sequences and their corresponding mutants, computed with the PersistNet model used in Fig. 5E.

Sup. Figure 8: Acute IMQ inflammation induces enduring DNA hypomethylation.

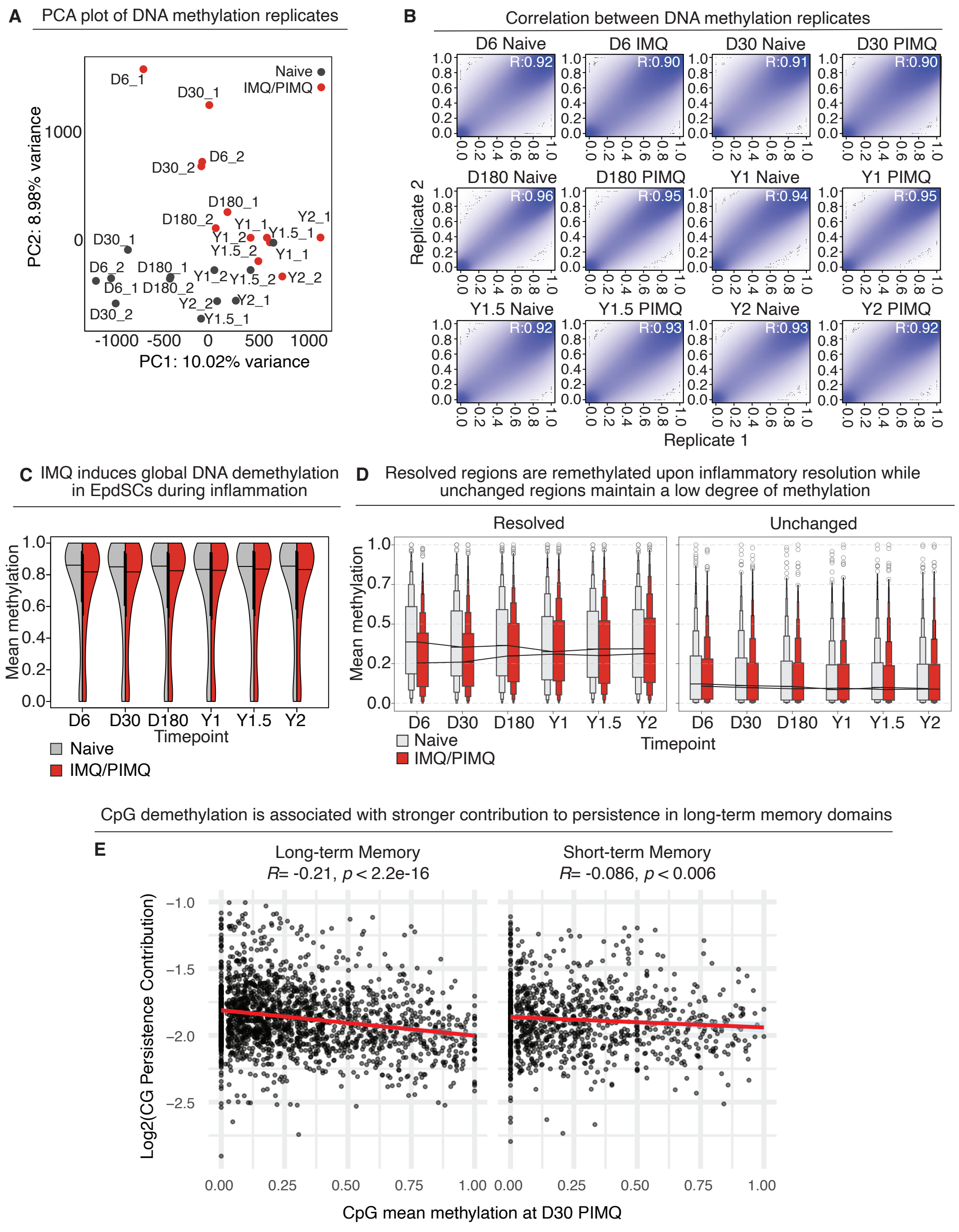

Fig. S8. Acute IMQ inflammation induces enduring DNA hypomethylation.
(**A**) Principal components analysis (PCA) of inter-sample variance between all whole genome bisulfite sequencing (WGBS) samples from isolated EpdSCs from naïve, IMQ and PIMQ conditions. Samples include *n* = 2 biological replicates per condition with 2-3 mice pooled per each biological replicate and timepoint. Data represent one experiment per timepoint. (**B**) Correlation plots between biological samples within each time point and condition. All biological samples display a correlation between replicates above 0.9. (**C**) Mean methylation rate of CpGs genome wide within IMQ-treated and naive conditions across the indicated time points. Note the inflammation-induced global reduction in DNA methylation that is slowly regained by ~Y1 PIMQ. (**D**) Mean CpG methylation rate within resolved (left) or unchanged (right) domains at the indicated timepoints. **(E)** Scatterplots relating CpG methylation levels at D30 PIMQ (x-axis) to Log_2 predicted persistence contribution (y-axis) in LT and ST memory domains. *r*, Pearson correlation coefficient.

Sup. Figure 9: PersistNet-predicted ELK/ETS motifs show ETS1 occupancy during inflammation and sustained demethylation across long-term memory domains.

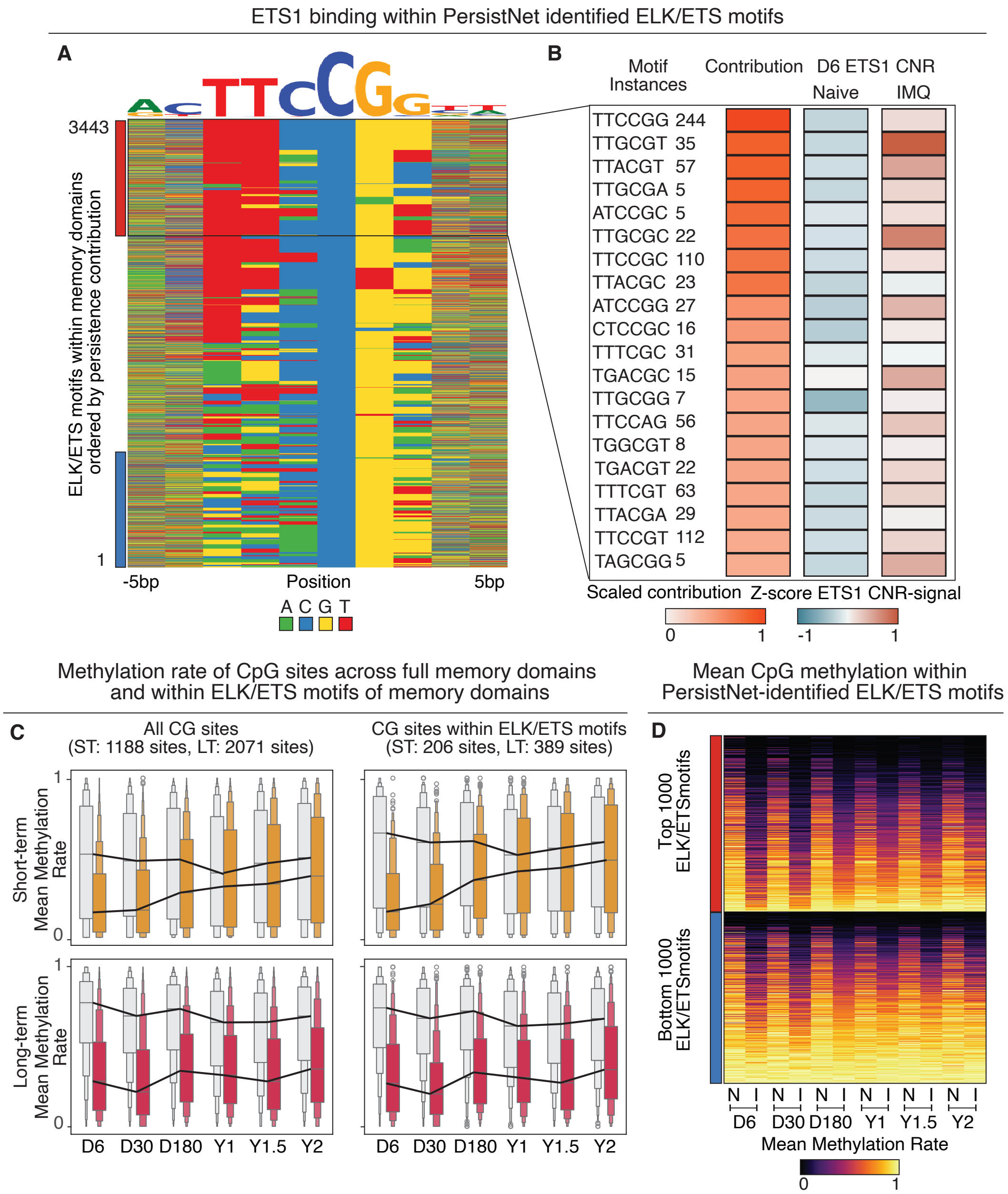

Fig. S9. PersistNet-predicted ELK/ETS motifs show ETS1 occupancy during inflammation and sustained demethylation across long-term memory domains.

(A) Heatmap showing all ELK/ETS motif instances in all 934 memory domains, ordered by their mean persistence contribution. Top, Contribution-weighted sequence logo of the PersistNet-derived ELK/ETS motif. Each row represents a motif instance aligned to its center, and colors denote nucleotide identity. Left bars highlight the top (highest-contributing) and bottom (lowest-contributing) 1000 ELK/ETS instances identified by PersistNet. **(B)** Table summarizing the most frequent ELK/ETS motif instances, their scaled persistence contribution and the corresponding Z-scored ETS1 Cut-and-Run signal in naïve and inflamed (D6 IMQ) conditions. **(C)** CpG methylation rate over time for all CpG sites (left) or those located within ELK/ETS motifs (right), separated into short-term (ST) and long-term (LT) memory domains. Each points represent a CpG site. Number of CpG sites within each memory domain are reported for each panel. **(D)** Heatmap showing mean methylation dynamics over time for the CpG sites within the top 1000 (upper panel) and bottom 1000 (lower panel) ELK/ETS motif instances ranked by persistence contribution. CpGs are ranked based on IMQ methylation signal within each panel. Note that CpGs within top-ranked motifs remain hypomethylated long after inflammatory resolution, whereas those in low-contribution motifs gradually regain methylation.

Sup. Figure 10: CpG-associated DNA sequence features support an unstable nucleosome structure within long-term memory domains.

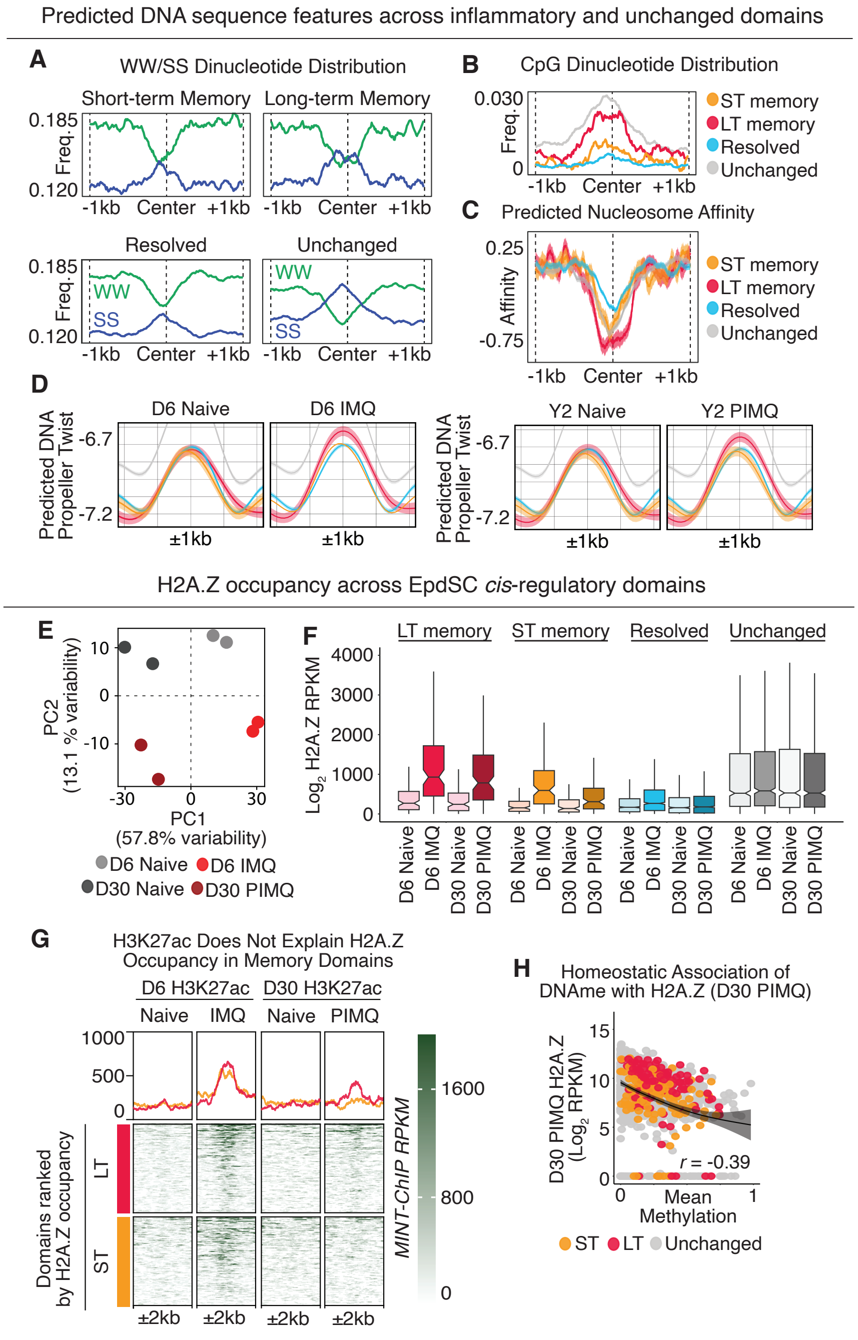

Fig. S10. CpG-associated DNA sequence features support an unstable nucleosome structure within long-term memory domains.
(**A**) Distribution of strong-strong (SS, CC/CG/GC/GG) or weak-weak (WW, AA/TT/AT/TA) dinucleotide motifs across ST memory, LT memory, resolved and unchanged domains. Average dinucleotide frequencies are shown across indicated domains, and centered over ATAC peak centers of ST or LT memory domains. **(B)** Distribution of CpG dinucleotides around domain centers of ST memory, LT memory, resolved and unchanged domains. (**C**) Predicted nucleosome binding affinity of indicated domains based on DNA sequence, assessed by chemical maps from 'NuPoP' as in Fig. 5C (*95*) (see also 'Methods'). Line thicknesses indicate standard errors of predictions. (**D**) Predicted propeller twist shape intensity of indicated domains based on DNA sequence and observed methylation status, using the 'DNAshape' method (*96*, see also 'Methods'). Note the selective relaxation of propeller twists within long-term memory domains upon inflammation-induced demethylation, which is not observed in any other set of domains. (**E**) PCA showing clustering of H2A.Z MINT-ChIP signal (derived from 1kb windows throughout the genome) among biological replicates, with striking variance between naïve and post-IMQ. (**F**) Boxplots of RPKM-normalized H2A.Z binding signal within the indicated domains, conditions and timepoints. Note the sustained increase in H2A.Z signal within LT memory domains (Red, burgundy), but not ST memory (oranges), resolved (blues) or unchanged domains (greys). **(G)** RPKM-normalized H3K27ac signal over LT and ST memory domains in D6 inflamed, D30 post-inflamed and matched naïve states. Domains are ordered top-to-bottom within each set (LT or ST) by overall H2A.Z signal across conditions (see Fig. 5C). **(H)** Scatterplot relating Log_2(RPKM+1)-transformed H2A.Z MINT-ChIP signal (y-axis) at D30 PIMQ to mean CpG methylation. Each point represents one domain (ST memory, LT memory or unchanged). Black lines show smoothed signals across plotted domains using a generalized additive model, with grey shaded ribbons to indicate standard error. *r* value indicates Spearman's rank correlation coefficient.

Sup. Figure 11: CpG enrichment and predicted nucleosome instability mark long-term memory domains of different cells from diverse tissues following exposure to acute inflammation.

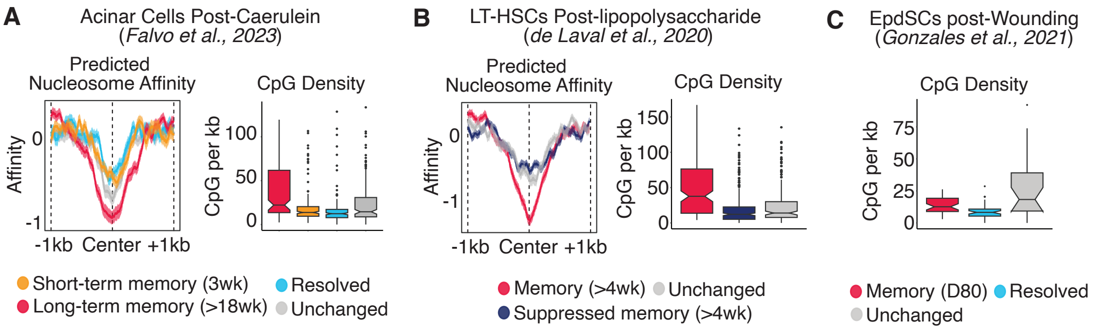

Sup. Figure 11: CpG enrichment and predicted nucleosome instability mark long-term memory domains of different cells from diverse tissues following exposure to acute inflammation.

(**A** to **C**) Re-analyses of publicly available ATAC-seq datasets for CpG enrichment and predicted nucleosome affinity within inflammation-sensitive and unchanged chromatin domains. Note the CpG enrichment and nucleosome disaffinity that marks long-lasting inflammatory memory sequences in diverse contexts, with CpG enrichments and predicted nucleosome affinity shown as in Fig. 5B. For (A), Acinar cells were exposed to pancreatitis via caerulein, and chromatin profiles analyzed at D2 (peak inflammation), week 3 (3wk, short-term memory) and week 18 (18wk, long-term memory) (*8*). For (B), Long-term hematopoietic stem cells (LT-HSCs) were exposed to lipopolysaccharide (LPS) and chromatin profiles analyzed at week 4 (4wk, memory). "*Suppressed memory*" domains represent domains which were less accessible than in post-LPS versus naïve cells at 4wk. Aggregate accessibility of these domains were demonstrated to return to baseline by 12wk post-LPS, in contrast to memory domains, which remained almost fully stable in aggregate accessibility by this time (*13*). For (C), skin was subject to partial thickness wounding, which elicits a wound response in hair follicle stem cells (HFSCs) and ultimate fate conversion to EpdSCs. "*Memory*" domains represent domains that were increased in accessibility over naïve HFSCs at the height of wounding at D3, then retained over naïve EpdSCs following their post-wound fate-switch and resolution at D80. "*Resolved*" domains were conversely induced by wounding at D3, but returned to baseline accessibilities by D80 (*71*). Predicted nucleosome affinity for (J) not shown due to insufficient number of memory domains. See 'Methods' for full reanalysis details and domain definitions.

Table S1: Antibodies

Antibody	Catalog	RRID	Dilution	Assay
anti-CD49f PE/Cyanine7	BioLegend cat. 313622	AB_2561705	1000x	EpdSC
anti-Sca-1 APC/Cyanine7	BioLegend cat. 108126	AB_10645327	500x	EpdSC
anti-CD29 Alexa Fluor 647	BioLegend cat. 102214	AB_492831	100x	EpdSC
anti-CD34 Alexa Fluor 700	ThermoFisher cat. 56-0341-82	AB_493998	50x	EpdSC
biotin anti-CD31	BioLegend cat. 102404	AB_312899	200x	EpdSC
biotin anti-CD45	BioLegend cat. 103104	AB_312969	200x	EpdSC
biotin anti-CD117	BioLegend cat. 105804	AB_313213	200x	EpdSC
biotin anti-CD140a	BioLegend cat. 135910	AB_2043974	200x	EpdSC
anti-CD45 Alexa Fluor 700	BioLegend cat. 103128	AB_493715	200x	Immune
anti-CD90.2 Alexa Fluor 488	BioLegend cat. 105316	AB_492886	200x	Immune
antiTCRγ/δ Brilliant Violet 421	BioLegend cat. 118120	AB_2562566	200x	Immune
anti-TCRβ PerCP/Cyanine5.5	BioLegend cat. 109228	AB_1575173	200x	Immune
anti-FoxP3 APC	ThermoFisher cat. 11-5773-82	AB_465243	200x	Immune
anti-Gata3 PE	ThermoFisher cat. 12-9966-42	AB_1963600	200x	Immune
anti-RORγt PE-CF594	BD Biosciences cat. 562684	AB_2651150	200x	Immune
H2A.Z rabbit	AbCam cat. ab4174	AB_304345	50x	MINT
H3K4me1 rabbit	Cell Signaling cat. 5326T	AB_10695148	33.3x	MINT
H3K27ac rabbit	Active Motif cat. 39134	AB_2561016	100x	MINT
c-FOS rabbit	Cell Signaling cat. 2250T	AB_2247211	50x	CNR
c-JUN rabbit	Cell Signaling cat. 9165T	AB_2130165	50x	CNR
deltaNP63 rabbit	BioLegend cat. 619002	AB_2207170	10x	CNR
ETS1 rabbit	Cell Signaling cat. 14069T	AB_2798383	50x	CNR

Table S2: TotalSeq antibodies and barcodes used in scRNA-seq

Antibody	Catalog	Sequence
TotalSeq™-B0301	BioLegend; cat. 155831	ACCCACCAGTAAGAC
TotalSeq™-B0302	BioLegend; cat. 155833	GGTCGAGAGCATTCA
TotalSeq™-B0303	BioLegend; cat. 155835	CTTGCCGCATGTCAT
TotalSeq™-B0304	BioLegend; cat. 155837	AAAGCATTCTTCACG
TotalSeq™-B0305	BioLegend; cat. 155839	CTTTGTCTTTGTGAG
TotalSeq™-B0306	BioLegend; cat. 155841	TATGCTGCCACGGTA
TotalSeq™-B0307	BioLegend; cat. 155843	GAGTCTGCCAGTATC
TotalSeq™-B0308	BioLegend; cat. 155845	TATAGAACGCCAGGC

Supplementary Data Captions

Data S1.
Bulk sequencing quality control metrics.

Data S2.
Memory, Resolved, and Unchanged domains genomic coordinates.

Data S3.
DESeq2 output from Day 6 IMQ versus Day 6 Control (bulk ATAC-seq).

Data S4.
DESeq2 output from Day 30 PIMQ versus Day 30 Control (bulk ATAC-seq).

Data S5.
DESeq2 output from Year 1 PIMQ versus Year 1 Control (bulk ATAC-seq).

Data S6.
Long-term and Short-term memory domain annotation.

Data S7.
DESeq2 output from Year 1 Control versus Day 30 Control (bulk ATAC-seq).

Data S8.
DESeq2 output from Day 30 PIMQ + 12h wounding versus Day 30 Control + 12h wounding (bulk RNA-seq).

Data S9.
DESeq2 output from Day 30 PIMQ + 6h TPA versus Day 30 Control + 6h TPA (bulk RNA-seq).

Data S10.
DESeq2 output from Year 1 PIMQ + 6h TPA versus Year 1 Control + 6h TPA (bulk RNA-seq).

Data S11.
DESeq2 output from Year 2 PIMQ + 6h TPA versus Year 2 Control + 6h TPA (bulk RNA-seq).

Data S12.
Diffxpy output from Undifferentiated basal (UB) – D30 PIMQ vs D30 Naïve (scRNA-seq).

Data S13.
Diffxpy output from Differentiating basal (DB) – D30 PIMQ vs D30 Naïve (scRNA-seq).

Data S14.
Diffxpy output from Undifferentiated basal (UB) – Y1 PIMQ vs Y1 Naïve (scRNA-seq).

Data S15.
Diffxpy output from Differentiating basal (DB) – Y1 PIMQ vs Y1 Naïve (scRNA-seq).

Data S16.
Z-scored accessibility for short-term domain across SEACells at D30 PIMQ and Y1 PIMQ (scATAC-seq).

Data S17.
Z-scored accessibility for long-term domain across SEACells at D30 PIMQ and Y1 PIMQ (scATAC-seq).

Data S18.
MotifMatchR motif scanning output from chromVARmotifs database across short-term and long-term domains.

Data S19.
MotifMatchR motif scanning output from Jaspar2020 database across short-term and long-term domains.

Data S20.
ChromBPNet (Establishment model) and PersistNet (persistence model) metrics and parameters.

Data S21.
ChromBPNet (Establishment model) TF discovery and scanning with Finemo across short-term and long-term domains.

Data S22.
PersistNet (persistence model) TF discovery and scanning with Finemo across short-term and long-term domains.

Data S23.
CpG Methylation aggregated score per short-term or long-term memory domain (Bisulfite sequencing).
